# Supplementary material for: An LC–MS/MS Method for the Simultaneous Analysis of 380 Pesticides in Soybeans, Kidney Beans, Black Soybeans, and Mung Beans: The Effect of Bean Grinding on Incurred Residues and Partitioning
Source: Foods. 2023 Dec 14;12(24):4477. doi: 10.3390/foods12244477 (PMC10742660; doi:10.3390/foods12244477)
Supplement: Supplementary file 1 [file foods-12-04477-s001.zip › foods-2704363-supplementary.pdf]

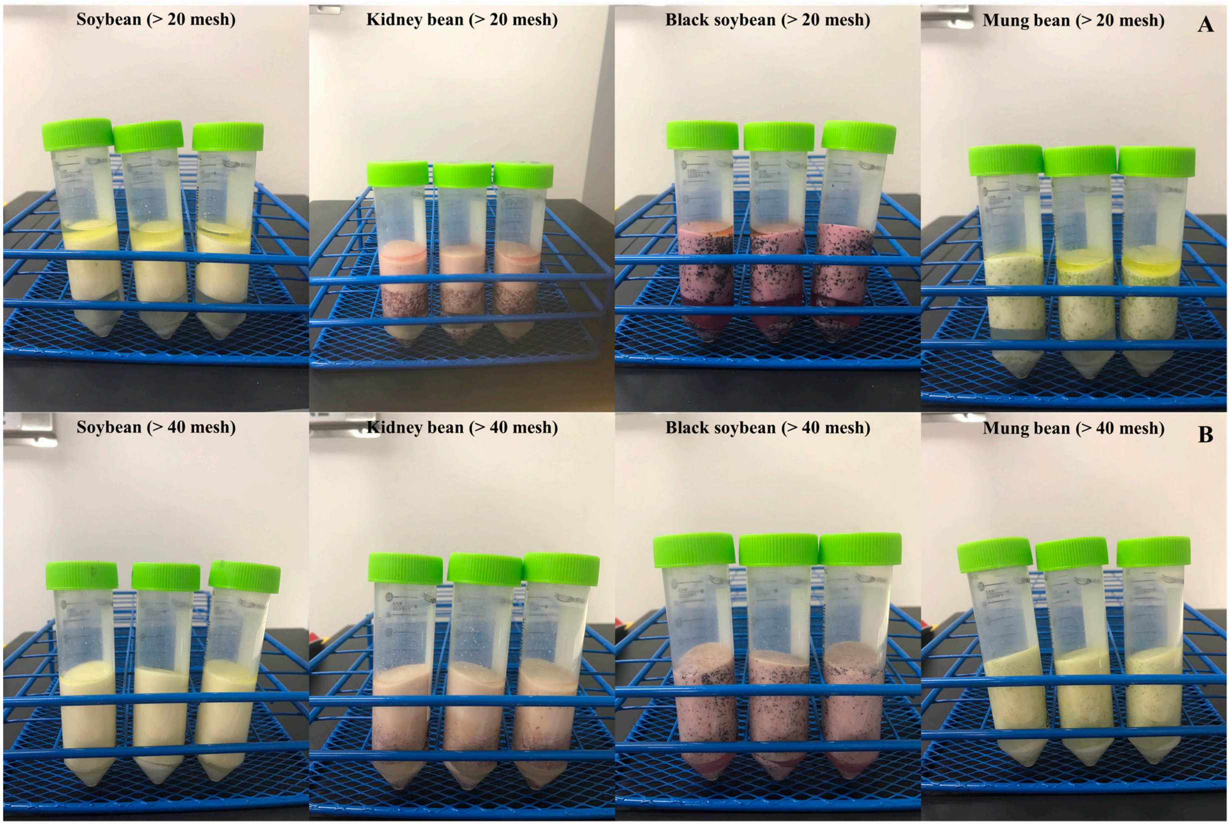

Figure S1. The amount of supernatant using sample preparation method 1 in soybean, kidney bean, black soybean, and mung bean after QuEChERS partitioning. (A) Soybean, kidney bean, black soybean, and mung bean > 20 mesh samples, respectively. (B) Soybean, kidney bean, black soybean, and mung bean > 40 mesh samples, respectively.

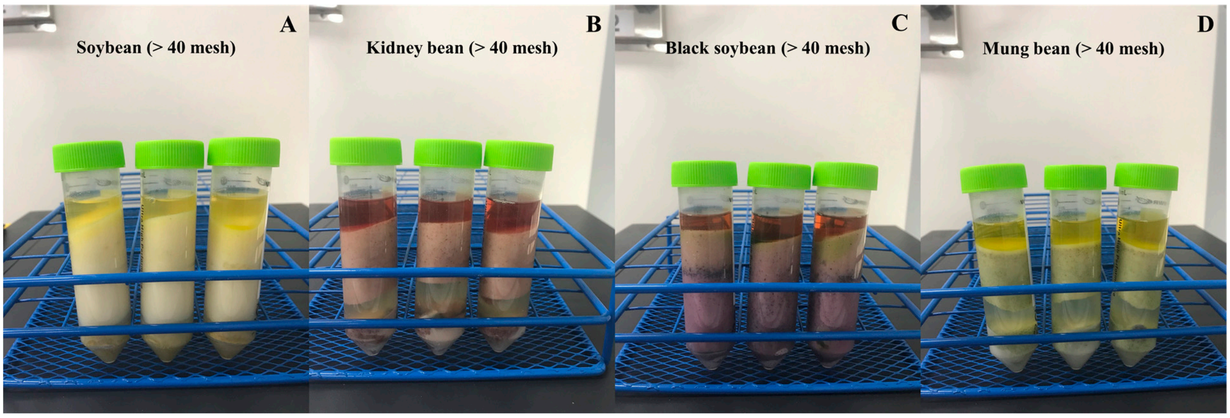

Figure S2. The amount of supernatant obtained using sample preparation method 2 in soybean, kidney bean, black soybean, and mung bean after QuEChERS partitioning. (A) soybean, (B) Kidney bean, (C) black soybean, and (D) mung bean, > 40 mesh samples, respectively.

Table S1. MRM condition (368 compounds for positive mode)

Compound name	Precursor ion	Product ion	Retention Time (min)	DP	EP	CE	CXP
2,3,5-trimethacarb	194.2	137.0	14.4	130	10	20	18
		122.1		130	10	35	14
3,4,5-trimethacarb	194.2	137.1	14.5	130	10	20	18
		122.0		110	10	35	14
3-hydroxycarbofuran	238.1	163.0	10.6	150	10	25	18
		181.1		150	10	15	18
Abamectin_B1a	890.5	305.3	17.6	10	10	35	10
		567.5		10	10	20	10
Acetamiprid	223.1	125.9	10.8	50	10	30	16
		90.1		50	10	45	10
Acetochlor	270.2	224.0	15.6	60	10	15	12
		148.0		50	10	25	20
		133.2		110	10	50	16
Acibenzolar-S-methyl	211.0	135.9	15.2	50	10	45	18
		91.0		60	10	25	10
		140.0		60	10	35	16
Alachlor	270.2	238.1	15.6	40	10	15	12
		148.0		40	10	30	20
		133.3		60	10	45	20
Aldicarb	208.1	116.0	12.2	30	10	15	14
		88.9		30	10	25	10
Aldicarb sulfone	240.0	86.1	7.2	30	10	30	10
		148.0		30	10	20	18
Ametoctradin	276.2	176.2	16.4	100	10	50	16
		149.1		100	10	50	16
Ametryn	228.2	186.2	14.6	60	10	30	16
		95.9		120	10	35	20
		116.1		40	10	35	16
Anilofos	368.0	199.1	15.8	40	10	20	12
		171.0		110	10	30	18
		125.0		40	10	50	12
Aramite	352.2	191.3	16.6	60	10	20	6
		255.2		60	10	15	20
		57.2		20	10	35	6
Aspon	379.0	211.0	17.1	70	10	30	20
		115.1		110	10	45	14
		253.1		70	10	20	20

Atrazine	216.1	174.1	14.4	120	10	25	16
		103.9		130	10	40	14
		176.1		20	10	25	6
Azaconazole	300.0	231.0	14.5	120	10	25	12
		158.9		30	10	40	18
		123.0		60	10	80	18
Azamethiphos	324.9	183.1	13.0	40	10	25	10
		112.0		30	10	50	14
Azimsulfuron	425.2	182.1	14.3	10	10	25	12
		156.2		10	10	55	20
Azinphos-methyl	318.0	132.1	14.7	50	10	25	20
		160.2		110	10	15	12
Azoxystrobin	404.1	372.1	14.8	50	10	20	18
		344.1		120	10	35	16
Benalaxyl	326.3	148.1	16.0	60	10	25	18
		208.1		60	10	25	20
		91.1		70	10	60	10
Bendiocarb	224.1	167.2	13.3	130	10	15	18
		109.1		120	10	25	10
Benfuracarb	411.1	190.1	16.5	60	10	20	6
		102.0		60	10	45	10
Benodanil	324.1	231.0	14.1	100	10	35	10
		203.1		30	10	50	20
		105.0		110	10	25	12
Benoxacor	260.1	149.2	14.7	140	10	25	20
		120.1		110	10	45	10
		149.2		150	10	25	16
Bensulfuron-methyl	411.1	148.9	14.6	10	10	25	18
		119.0		10	10	55	10
Bensulide	398.0	158.0	15.7	10	10	35	18
		218.1		10	10	25	6
Benthiavalicarb-isopropyl	382.2	180.1	15.2	40	10	40	6
		116.1		30	10	35	14
Benzobicyclon	447.0	257.1	15.2	100	10	35	14
		229.1		120	10	55	12
Benzoximate	364.0	199.1	16.2	50	10	30	20
		105.1		70	10	45	12
Benzoylprop-ethyl	366.2	104.9	15.9	70	10	35	12
		77.0		70	10	75	18
		105.1		20	10	30	12

Bixafen	413.9	265.9	15.6	70	10	45	18
		374.1		30	10	35	12
Boscalid	343.0	307.0	15.0	10	10	30	4
		140.0		10	10	30	4
Bromacil	261.0	205.0	13.3	60	10	20	10
		188.0		70	10	40	16
Bromobutide	312.2	194.0	15.6	30	10	20	18
		119.2		110	10	35	12
		196.0		110	10	20	10
Bupirimate	317.2	166.2	15.6	120	10	35	20
		108.0		120	10	35	18
		272.0		90	10	30	8
Buprofezin	306.2	201.1	16.6	70	10	20	12
		116.2		110	10	25	18
Cadusafos	271.0	131.0	16.3	30	10	35	20
		159.0		60	10	20	16
Cafenstrole	351.1	100.1	15.3	140	10	25	12
		72.1		110	10	50	6
Carbaryl	202.1	144.9	13.7	120	10	20	18
		127.1		120	10	40	16
Carbendazim	192.2	160.2	8.5	130	10	25	18
		132.1		130	10	40	16
Carbetamide	237.2	120.0	12.8	40	10	25	18
		72.1		60	10	35	16
Carbofuran	222.1	165.2	13.4	60	10	20	10
		123.0		30	10	30	12
Carbofuran-3-hydroxy	238.0	163.0	10.6	150	10	20	18
		107.0		120	10	40	12
Carboxin	236.1	142.9	13.7	30	10	25	18
		87.0		30	10	35	10
Carfentrazone-ethyl	412.0	366.1	15.8	150	10	25	12
		346.1		150	10	35	18
Carpropamid	334.1	196.1	15.9	110	10	20	20
		139.0		80	10	35	16
		103.0		110	10	55	12
Chlorantraniliprole	484.0	452.9	14.6	30	10	25	14
		285.8		80	10	25	10
Chlorbenzuron	309.2	156.0	15.8	20	10	20	10
		138.9		20	10	50	18
Chlorfenvinphos	359.0	99.1	15.9	120	10	50	12

		170.1		90	10	55	10
		155.1		120	10	20	20
Chlorfluazuron	539.9	383.0	17.1	20	10	30	6
		347.0		20	10	60	6
Chloridazon	222.0	104.0	10.8	50	10	30	12
		92.0		50	10	35	10
Chlorimuron-ethyl	415.0	185.9	15.1	10	10	25	6
		120.9		10	10	60	18
Chlorotoluron	213.0	72.0	14.1	30	10	35	8
		140.0		30	10	30	8
Chloroxuron	291.2	72.2	15.3	30	10	50	12
		164.0		90	10	25	20
		218.2		110	10	40	10
Chlorpyrifos	350.0	198.0	16.9	30	10	30	20
		96.9		30	10	50	12
Chlorpyrifos-methyl	323.8	125.2	16.3	30	10	30	12
		291.8		20	10	25	18
Chlorsulfuron	358.0	141.0	13.7	10	10	25	4
		167.0		10	10	25	20
Chromafenozide	395.2	175.0	15.4	110	10	35	18
		147.1		100	10	60	16
Clethodim	360.1	268.2	16.4	20	10	20	10
		164.0		20	10	25	14
Clofentezine	303.0	138.0	16.2	20	10	25	20
		102.1		20	10	60	6
Clomazone	240.1	125.0	14.8	120	10	30	16
		89.0		40	10	60	10
Clomeprop	324.0	203.0	16.6	20	10	25	6
		120.1		20	10	35	6
Clothianidin	250.0	169.1	10.0	70	10	20	18
		132.0		70	10	25	16
Coumaphos	363.1	227.1	16.0	150	10	35	14
		307.1		150	10	25	6
		335.0		140	10	25	10
Crotoxyphos	332.3	211.0	15.0	80	10	15	6
		127.0		60	10	40	14
		104.9		70	10	40	14
Crufomate	292.0	235.9	15.7	140	10	30	14
		107.9		130	10	40	12
Cyanazine	241.2	214.1	13.0	100	10	25	12

		103.9		100	10	45	12
		216.1		10	10	25	12
Cyantraniliprole	475.0	285.9	13.8	10	10	25	4
		112.1		10	10	80	20
Cyazofamid	325.0	108.0	15.5	80	10	25	12
		261.0		50	10	15	20
Cyclaniliprole	599.9	284.0	15.6	30	10	25	6
		177.0		20	10	70	20
Cycloate	216.1	154.1	16.4	30	10	20	8
		134.1		30	10	20	16
Cyclosulfamuron	422.0	261.0	15.3	20	10	25	8
		218.0		20	10	40	6
Cyenopyrafen	394.2	310.1	17.2	10	10	35	10
		254.0		10	10	45	14
Cyflufenamid	413.1	295.2	16.0	90	10	20	10
		241.0		20	10	35	14
Cyflumetofen	465.1	173.1	16.3	70	10	30	18
		249.0		20	10	20	8
Cymoxanil	199.0	128.2	11.2	130	10	15	16
		111.1		130	10	25	12
Daimuron	269.1	151.1	15.2	110	10	20	18
		119.1		110	10	25	18
Demeton-O	258.9	89.2	14.8	30	10	30	10
		61.2		30	10	50	10
Demeton-S	259.0	89.1	14.8	40	10	25	20
		61.0		30	10	45	8
Demeton-S-methyl	231.0	61.0	13.4	60	10	40	10
		89.0		30	10	25	18
Demeton-S-methyl sulfoxide	247.0	169.0	8.1	20	10	20	20
		109.0		20	10	40	12
Demeton-S-methyl-sulfone	262.9	169.0	8.4	140	10	25	20
		109.0		120	10	40	12
Demeton-S-sulfone	291.0	234.9	11.3	120	10	20	12
		263.0		120	10	20	8
Demeton-S-sulfoxide	275.0	197.0	11.1	40	10	20	20
		141.0		30	10	30	20
Desmetryn	214.1	172.1	13.8	70	10	25	10
		82.2		70	10	45	14
		57.2		40	10	45	16
Diazinon	305.1	169.1	16.0	130	10	25	20

		153.3		60	10	25	14
Dichlorvos	220.9	109.1	13.2	150	10	25	12
		127.2		120	10	25	14
Diclosulam	405.9	160.9	13.9	10	10	35	4
		378.0		10	10	25	4
Dicrotophos	238.0	112.1	9.5	30	10	20	12
		127.0		30	10	25	16
Diethatyl-ethyl	312.3	162.2	15.8	110	10	35	20
		147.1		60	10	55	16
		132.1		40	10	70	18
Diethofencarb	268.1	226.1	14.9	80	10	15	20
		180.0		60	10	25	20
Diflufenican	395.1	266.1	16.3	20	10	35	18
		246.2		60	10	50	20
		237.9		20	10	55	14
Dimepiperate	264.1	145.9	16.4	70	10	25	20
		119.0		50	10	25	20
Dimethachlor	256.2	224.2	14.6	40	10	20	12
		148.2		30	10	35	18
		226.2		40	10	20	12
Dimethametryn	256.2	186.1	15.8	120	10	30	10
		68.0		120	10	55	8
Dimethenamide	276.1	244.1	15.1	80	10	20	20
		168.3		30	10	30	12
Dimethoate	230.0	198.9	10.5	30	10	15	6
		125.1		30	10	30	12
		171.1		30	10	20	18
Dimethylaminosulfotoluidide (DMST)	215.2	106.1	13.5	130	10	20	12
		79.1		10	10	40	4
		77.1		10	10	55	4
Diniconazole	326.0	70.0	16.2	20	10	65	18
		159.0		90	10	45	20
Diphenamid	240.1	134.1	14.6	30	10	30	20
		167.2		10	7.5	30	12
Disulfoton-sulfone	307.0	125.0	14.1	150	10	25	6
		152.9		150	10	20	8
Disulfoton-sulfoxide	291.0	185.0	14.1	20	10	20	18
		128.9		20	10	40	14
Dithiopyr	402.1	353.9	16.3	60	10	25	12
		272.1		110	10	45	20

Diuron	233.1	72.1	14.0	70	10	40	12
		160.0		100	10	35	20
Edifenphos	310.9	109.0	15.9	120	10	45	18
		111.1		120	10	30	18
Emamectin B1a	886.5	158.1	16.3	20	10	40	12
		82.1		20	10	85	10
Epoxiconazole	330.2	121.0	15.5	130	10	35	6
		101.0		130	10	70	12
		121.0		20	10	30	12
Esprocarb	266.1	91.0	16.7	30	10	45	18
		65.0		30	10	80	20
Etaconazole	328.1	159.1	15.6	50	10	45	20
		204.9		50	10	25	12
		161.1		60	10	50	16
Ethaboxam	321.0	183.1	14.0	130	10	35	8
		200.1		80	10	35	20
Ethiofencarb	226.1	164.0	13.9	130	10	15	18
		169.0		130	10	10	18
Ethirimol	210.0	140.1	12.2	90	10	30	16
		98.1		130	10	40	12
Ethoprophos	243.0	131.0	15.6	60	10	30	14
		97.0		60	10	45	12
Ethoxysulfuron	399.1	261.0	15.1	20	10	25	10
		218.2		30	7	35	18
Etoxazole	360.2	141.0	17.1	110	10	45	16
		57.2		110	10	45	10
Etrimfos	293.1	125.0	16.0	30	10	40	12
		265.0		100	10	25	14
Famoxadone	392.0	331.0	15.8	20	10	15	6
		238.0		20	10	25	12
Fenamiphos	304.2	217.1	15.6	60	10	35	10
		202.1		60	10	50	18
Fenamiphos sulfone	337.1	267.1	13.6	120	10	30	20
		109.1		10	10	55	18
Fenamiphos sulfoxide	320.0	171.0	13.5	140	10	30	18
		233.1		140	10	35	12
Fenamiphos-sulfone	336.1	307.9	13.6	150	10	20	10
		265.9		150	10	30	8
Fenamiphos-sulfoxide	320.1	233.0	13.5	140	10	35	12
		171.0		30	10	30	18

Fenarimol	331.0	268.1	15.5	110	10	30	14
		189.0		150	10	70	6
Fenazaquin	307.2	161.2	17.9	20	10	25	18
		147.0		20	10	25	18
Fenbuconazole	337.1	125.1	15.5	20	10	60	18
		70.0		20	10	30	8
Fenfuram	202.1	109.0	13.8	30	10	30	18
		120.1		30	10	25	14
		53.1		30	10	55	6
Fenobucarb	208.1	95.0	14.8	120	10	20	8
		152.0		120	10	15	18
Fenothiocarb	254.1	160.1	15.8	60	10	15	18
		72.0		30	10	35	18
		77.1		60	10	65	18
Fenoxanil	329.1	302.1	15.6	150	10	20	10
		86.1		150	10	25	18
		304.1		150	10	20	10
Fenoxaprop-ethyl	362.1	288.1	16.4	20	10	25	20
		77.1		50	10	80	8
Fenoxycarb	302.2	116.2	15.7	20	10	15	6
		88.0		20	10	25	12
Fenpropimorph	304.2	147.2	14.7	130	10	40	18
		117.2		130	10	85	16
Fenpyroximate	422.2	366.2	17.3	110	10	25	12
		138.0		20	10	45	16
Fenquinotrione_KIH-3635-M-2	331.1	259.1	13.2	10	10	35	4
		216.2		10	10	55	4
Fensulfothion	309.0	281.0	14.4	100	10	20	10
		157.0		120	10	35	20
Fenthion oxon	263.0	231.0	14.7	140	10	20	10
		216.0		140	10	35	12
Fenthion oxon sulfone	295.0	217.0	11.2	150	10	25	12
		104.1		150	10	35	12
Fenthion oxon sulfoxide	279.0	104.0	10.9	70	10	40	12
		109.1		130	10	50	12
Fenthion sulfoxide	295.1	280.0	13.6	150	10	25	18
		109.1		40	10	40	6
Fenthion-sulfone	310.9	124.9	13.8	150	10	35	12
		278.9		10	10	25	14
Ferimzone	255.1	91.1	14.8	50	10	45	14

		65.1		50	10	70	18
Flamprop-isopropyl	364.1	304.1	15.9	70	10	15	20
		105.1		30	10	35	16
		77.1		30	10	85	14
Flonicamid	230.0	203.2	8.2	120	10	25	18
		97.8		120	10	55	12
Fluacrypyrim	427.2	145.0	16.2	60	10	40	20
		205.1		50	10	20	20
Flucetosulfuron	488.0	156.1	14.7	10	10	25	4
		273.0		10	10	35	4
Flufenacet	364.1	152.2	15.4	50	10	25	18
		194.2		60	10	15	20
Flufenoxuron	489.1	158.2	16.8	20	10	25	6
		141.2		20	10	70	6
Fluometuron	233.1	145.1	14.0	30	10	45	16
		168.0		30	10	35	20
Fluopicolide	383.0	172.9	15.2	80	10	30	16
		109.0		80	10	90	12
Fluopyram	397.1	208.0	15.3	60	10	30	18
		173.0		60	10	35	18
		145.0		60	10	75	18
Flupyradifurone	289.0	125.9	10.7	110	10	30	14
		72.9		60	10	90	8
		99.0		40	10	65	12
Fluquinconazole	376.0	307.1	15.4	110	10	40	10
		349.0		100	10	30	12
Fluridone	330.0	309.1	14.7	150	10	45	18
		294.1		110	10	65	10
Flurochloridone	312.2	292.2	15.2	150	10	30	18
		145.1		150	10	75	16
		145.1		30	10	55	18
Flurtamone	334.1	247.0	14.9	120	10	40	12
		178.1		40	10	65	20
		227.2		40	10	50	12
Flusilazole	316.1	247.1	15.6	60	10	25	14
		165.1		60	10	35	20
Fluthiacet-methyl	404.1	274.1	15.8	20	10	40	8
		239.1		70	10	50	8
		187.9		40	10	65	16
Flutianil	427.0	192.2	15.7	90	10	35	12

		411.1		90	10	45	12
		132.1		60	10	70	14
Flutolanil	324.1	262.1	15.1	60	10	25	20
		242.2		120	10	35	16
Flutriafol	302.2	70.1	14.2	80	10	55	18
		95.1		30	10	75	12
Fluxametamide	474.0	399.9	16.7	20	10	25	14
		159.9		20	10	50	16
Fluxapyroxad	382.0	362.0	15.1	70	10	20	10
		342.0		90	10	30	20
Forchlorfenuron	248.2	129.1	14.4	70	10	25	20
		93.1		80	10	45	12
Fosthiazate	284.0	104.0	14.0	30	10	30	12
		228.0		40	10	15	20
Furathiocarb	383.2	252.2	16.6	100	10	20	14
		195.0		100	10	25	6
Halosulfuron-methyl	435.1	182.0	15.4	50	10	30	20
		139.1		30	10	70	16
Heptenophos	251.1	127.0	14.5	120	10	20	18
		125.1		120	10	25	16
		109.0		120	10	45	12
Hexazinone	253.2	171.1	13.4	110	10	25	12
		71.1		110	10	40	20
Hexythiazox	353.1	228.0	16.9	20	10	25	20
		168.1		20	10	35	20
Imazalil	297.1	159.0	13.9	70	10	40	14
		255.0		70	10	30	14
Imazapic	276.1	163.1	11.5	30	10	35	20
		216.3		30	10	35	12
Imazaquin	312.1	199.1	13.4	100	10	40	6
		267.2		130	10	30	16
Imazethapyr	290.1	245.2	12.8	30	10	30	12
		177.0		30	10	40	20
Imicyafos	305.1	201.1	13.0	50	4.5	30	6
		235.2		60	4.5	25	12
Imidacloprid	256.1	175.0	9.9	10	10	30	18
		209.0		10	10	25	18
Inabenfide	339.0	321.1	14.8	80	10	25	10
		80.1		100	10	35	8
Indanofan	341.2	175.0	15.5	60	10	25	18

		187.2		40	10	20	10
		115.2		60	10	75	6
Indoxacarb	527.9	203.0	16.1	30	10	57	22
		149.9		30	10	33	18
Ipconazole	334.2	70.2	16.3	80	10	80	8
		125.0		70	10	60	14
Ipfencarbazone	427.0	198.0	15.8	60	10	20	6
		156.0		60	10	35	20
Iprobenfos	289.1	91.2	15.8	60	10	35	12
		205.0		60	10	15	18
Iprovalicarb	321.3	119.2	15.4	110	10	30	16
		203.2		70	10	15	10
Isoprocarb	194.1	95.1	14.2	130	10	25	14
		137.0		120	10	20	14
Isoprothiolane	291.1	231.1	15.2	60	10	15	12
		189.0		60	3.5	30	10
Isoproturon	207.1	72.1	14.4	50	10	25	12
		165.1		30	10	20	18
Isopyrazam	360.1	244.1	16.2	100	10	35	12
		320.1		20	10	30	10
Isoxaben	333.2	165.2	15.1	30	10	35	18
		107.2		90	10	85	12
Isoxathion	314.2	105.0	16.0	70	10	25	12
		97.1		100	10	55	12
		170.1		70	10	20	20
Lenacil	235.1	153.1	14.4	30	10	25	8
		136.0		80	10	50	18
Linuron	249.0	182.1	14.9	60	10	25	14
		159.9		90	10	25	16
Lufenuron	510.9	158.2	16.5	20	10	30	6
		141.2		20	10	70	6
Malaoxon	315.0	127.0	13.4	100	10	20	16
		99.0		100	10	40	14
Mandestrobin	314.1	192.0	15.9	60	10	20	18
		132.0		60	10	35	18
Mandipropamid	412.1	328.1	15.0	120	10	20	16
		125.1		150	10	55	18
Mefenacet	299.1	148.1	15.3	50	10	20	18
		120.1		70	10	40	12
Mefentrifluconazole	398.0	70.1	15.9	120	10	55	18

		182.0		90	10	45	18
Mepanipyrim	224.1	106.1	15.5	100	10	35	8
		66.1		100	10	55	12
Mephosfolan	269.9	139.9	13.2	60	10	35	16
		195.9		60	10	20	6
Mepronil	270.1	119.0	15.2	70	10	30	12
		228.1		60	10	20	12
Metaflumizone (E)	507.0	287.2	16.1	20	10	35	6
		178.0		10	10	35	6
Metaflumizone (Z)	507.0	178.0	16.1	20	10	35	6
		287.2		20	10	35	6
Metalaxyl	280.1	220.0	14.4	40	10	20	12
		192.3		120	10	25	10
Metamifop	441.1	288.1	16.4	90	10.5	25	16
		180.2		70	10.5	25	6
Metamitron	203.1	175.2	10.4	60	10	25	10
		104.1		70	10	35	12
Metazosulfuron	476.0	182.0	14.8	10	4.5	25	20
		295.2		10	4.5	20	6
Metconazole	320.1	70.1	16.0	60	10	60	18
		125.0		20	10	65	16
Methabenzthiazuron	222.2	165.1	14.3	70	10	30	10
		150.3		100	10	50	16
Methamidophos	142.1	94.0	3.4	20	10	20	12
		112.1		100	10	10	20
Methiocarb	226.1	169.1	13.9	50	10	15	18
		107.0		50	10	50	20
Methiocarb-sulfone	275.0	201.0	11.1	40	10	20	6
		122.2		30	10	30	12
Methiocarb-sulfoxide	242.1	185.0	10.4	60	10	20	10
		122.2		30	10	40	14
Methomyl	163.1	88.1	8.2	40	10	15	10
		106.0		40	10	15	12
Methoprotryne	272.2	240.1	14.7	30	10	25	14
		170.0		30	10	40	18
		198.1		30	10	30	18
Methoxyfenozide	369.2	149.1	15.2	90	10	25	8
		313.2		90	10	15	10
Metobromuron	259.0	170.0	14.2	90	10	25	16
		148.2		110	10	25	10

Metolcarb	166.1	109.1	12.8	130	10	20	12
		91.1		120	10	35	12
Metominostrboin (Z)	285.1	196.1	14.2	50	10	20	10
		77.1		70	10	55	10
Metominostrobin (E)	285.1	194.1	14.5	80	10	30	20
		196.1		70	10	25	16
Metrafenon	409.1	209.1	16.1	60	10	25	12
		226.9		20	10	35	20
Metrafenone	409.0	209.1	16.1	60	10	25	12
		227.0		60	10	30	12
Mevinphos	225.0	127.0	11.7	120	10	25	16
		193.0		120	10	10	20
Molinate	188.1	126.1	15.4	20	10	20	12
		98.2		60	10	25	16
Monocrotophos	224.1	127.0	8.9	130	10	25	16
		98.0		130	10	20	12
Monolinuron	215.1	126.0	13.9	70	10	25	18
		148.1		60	10	25	16
		99.1		60	10	45	12
Napropamide	272.1	129.3	15.5	30	10	20	14
		171.0		120	10	25	18
Neburon	275.0	88.0	15.8	100	10	25	12
		114.1		110	10	20	12
Nicosulfuron	411.1	182.1	14.6	10	10	30	12
		213.0		10	10	25	10
Nitenpyram	271.0	225.1	7.6	70	10	15	12
		126.0		30	10	40	16
Norflurazon	304.1	284.1	14.5	70	10	35	16
		160.0		70	10	40	16
		140.0		60	10	50	16
Noruron (Norea)	223.2	67.1	15.0	80	10	55	10
		93.0		130	10	40	12
Nuarimol	315.1	252.1	14.9	130	10	30	8
		81.1		100	10	55	4
Ofurace	282.1	254.0	13.4	150	10	20	8
		160.0		140	10	35	20
Orysastrobin	392.1	205.0	15.1	80	10	20	18
		116.0		90	10	35	16
Oxadiazon	362.1	303.1	16.7	20	10	25	6
		219.9		20	10	35	20

Oxadixyl	279.2	219.2	12.6	130	10	15	12
		132.1		140	10	50	16
Oxamyl	237.1	90.1	7.6	40	10	10	12
		72.0		40	10	30	8
Oxathiapiprolin	540.1	500.1	15.0	10	10	35	18
		350.0		10	10	45	12
Oxaziclomefone	376.1	190.1	16.5	130	10	25	6
		161.1		130	10	40	18
Oxycarboxin	268.0	175.0	11.2	110	10	25	20
		147.0		110	10	30	14
Oxydemeton-methyl	247.0	169.0	8.1	40	10	20	20
		109.0		20	10	40	12
Paraoxon-methyl	248.0	202.1	12.6	150	10	25	10
		90.1		150	10	35	10
Pebulate	204.1	128.1	16.3	40	10	15	18
		72.0		80	10	20	12
Penconazole	284.1	158.9	15.9	100	10	35	20
		70.0		80	10	25	14
Pencycuron	329.1	125.1	16.1	60	10	35	14
		89.1		40	10	95	10
Penoxsulam	484.0	195.1	13.8	10	10	40	10
		164.1		10	10	45	18
Phenothrin_cis	351.1	183.1	18.0	20	10	35	6
		128.1		10	10	85	16
Phenthoate	321.0	79.0	15.7	80	10	60	12
		163.1		90	10	20	20
Phorate	261.0	75.0	10.7	70	10	25	10
		97.0		70	10	40	12
Phorate-oxon	245.0	75.0	14.6	40	10	25	10
		47.0		40	10	45	12
Phorate-oxon-sulfone	277.0	111.0	11.2	130	10	30	6
		155.0		130	10	20	18
Phorate-oxon-sulfoxide	261.0	111.0	10.7	50	10	35	14
		97.0		50	10	40	12
Phorate-sulfone	293.0	170.8	14.1	140	10	20	16
		114.9		140	10	35	14
Phorate-sulfoxide	277.0	199.0	14.1	70	10	15	18
		143.0		60	10	30	8
Phosalone	368.0	182.1	16.0	140	10	25	6
		138.0		140	10	45	20

		184.1		140	10	25	10
Phosfolan	256.0	139.8	12.1	40	10	35	16
		227.9		40	10	20	12
Phosmet-oxon	302.0	160.1	12.6	90	10	30	18
		77.1		110	10	70	8
Phosphamidon	300.1	174.0	12.9	50	10	20	20
		127.0		50	10	40	16
Phoxim	299.0	77.0	16.0	30	10	55	14
		129.1		60	10	20	16
Picarbutrazox	410.1	310.0	15.5	30	10	20	12
		107.1		20	10	40	12
Picolinafen	377.1	238.1	16.6	20	10	45	20
		359.1		20	10	30	20
		145.1		20	10	70	18
Picoxystrobin	368.1	205.1	15.6	40	10	15	20
		145.1		70	10	35	20
		115.1		70	10	65	6
Pinoxaden	401.1	317.2	16.0	130	10	30	20
		289.2		130	10	50	10
Piperonyl butoxide	356.3	177.1	16.8	40	10	25	10
		119.0		130	10	50	14
		147.2		60	10	45	14
Piperophos	354.1	171.1	16.3	40	10	35	18
		255.0		90	10	20	18
Pirimicarb	239.2	182.2	12.8	50	10	25	10
		72.1		40	10	40	8
Pirimicarb-desmethyl	225.1	72.2	9.8	40	10	40	8
		168.2		40	10	20	18
Pirimiphos-ethyl	334.1	198.1	16.7	40	10	30	18
		182.2		130	10	35	20
Pirimiphos-methyl	306.1	108.0	16.1	60	10	45	16
		164.1		60	10	30	20
Probenazole	224.1	41.0	12.9	120	10	35	10
		184.1		140	10	35	20
Prochloraz	376.1	308.0	16.0	80	10	20	6
		266.0		80	10	25	16
		69.9		80	10	35	12
Profenofos	372.9	302.9	16.5	150	10	30	18
		97.0		150	10	50	20
Promecarb	208.1	151.1	15.1	130	10	15	16

		109.1		80	10	25	14
Prometryn	242.1	200.2	15.3	60	10	25	14
		158.1		60	10	35	14
Pronamide (Propyzamide)	256.1	172.9	15.2	30	10	30	16
		189.9		100	10	25	6
		109.1		130	10	65	12
Propachlor	212.1	170.1	14.4	70	10	35	16
		152.0		30	10	30	16
		94.0		30	10	40	12
Propanil	218.0	162.0	15.0	130	10	25	18
		127.1		70	10	40	16
Propaquizafop	444.1	100.0	16.6	20	10	25	12
		299.0		20	10	35	10
Propargite	368.2	231.1	17.0	20	10	15	20
		175.1		30	10	25	18
Propazine	230.2	146.0	15.1	70	10	35	16
		188.1		70	10	25	10
		104.1		120	10	45	20
Propiconazole	342.2	69.1	16.0	20	10	30	8
		159.0		20	10	40	18
		123.1		70	10	85	12
Propoxur	210.1	111.1	13.3	70	10	20	6
		168.1		70	10	10	10
Proquinazid	373.0	331.0	17.6	20	10	20	18
		330.9		20	10	20	18
		289.1		20	10	35	6
Prosulfocarb	252.1	91.1	16.5	40	10	50	12
		65.1		40	10	80	14
Prothioconazole-desthio	312.1	70.0	15.6	80	10	55	18
		125.0		90	10	50	18
Pydiflumetofen	426.1	192.9	16.2	20	10	60	10
		123.1		90	10	100	18
Pyflubumide	536.2	155.1	16.7	20	10	35	10
		111.1		60	10	85	12
Pyflubumide-NH	466.1	137.0	16.4	20	10	65	16
		382.0		20	10	50	12
Pyracarbolid	218.2	125.1	13.5	40	10	25	14
		55.0		60	10	45	8
		97.2		30	10	40	10
Pyraclofos	361.0	257.0	16.0	20	10	30	8

		138.1		20	10	55	16
Pyraclonil	315.1	169.0	14.0	70	10	50	18
		241.2		30	10	40	20
Pyraclostrobin	388.1	194.0	16.0	40	10	20	6
		163.0		40	10	35	18
Pyraflufen-ethyl	413.0	339.1	15.8	20	10	30	10
		252.9		20	10	45	8
Pyraziflumid	380.0	146.9	15.3	30	10	50	18
		68.9		20	10	95	16
Pyrazolate	439.0	173.1	16.1	100	9	25	18
		155.2		60	9	25	18
Pyrazophos	374.1	222.1	16.1	20	10	30	8
		194.0		20	10	45	6
Pyrazoxyfen	403.0	91.0	15.9	110	10	55	12
		105.0		20	10	25	12
Pyribencarb	362.1	239.0	14.8	80	10	25	12
		207.0		110	10	40	20
Pyributicarb	331.1	181.1	16.8	100	10	25	18
		108.0		130	10	45	18
Pyridaben	365.1	309.1	17.6	20	10	20	6
		147.0		20	10	35	18
Pyridaphenthion	341.0	189.0	15.2	20	10	30	10
		205.0		30	10	30	20
Pyrifluquinazon	465.1	423.1	15.3	70	10	30	20
		92.2		60	10	75	20
Pyriftalid	319.1	139.0	14.8	100	10	40	18
		179.0		30	10	40	20
Pyrimethanil	200.0	107.0	14.9	130	10	35	6
		82.0		130	10	35	20
Pyrimidifen	378.2	184.1	16.9	20	10	35	6
		157.1		20	10	50	18
Pyrimisulfan	420.1	370.1	14.4	10	10	25	16
		255.2		10	10	35	14
Pyriofenone	366.1	184.1	16.2	20	10	35	6
		209.1		100	10	35	10
Pyriproxyfen	322.1	96.2	16.8	130	10	20	10
		185.0		100	10	35	18
Pyroquilon	174.1	132.1	13.2	40	10	35	18
		117.2		70	10	45	14
Quinoclamine	208.1	105.0	13.1	60	10	35	12

		89.0		40	10	50	10
Quizalofop-ethyl	373.1	298.9	16.5	20	10	25	10
		271.0		60	10	35	14
Saflufenacil	501.1	349.1	14.6	10	10	40	4
		197.9		10	10	60	4
Secbumeton	226.1	170.1	14.1	100	10	25	14
		100.0		30	10	40	12
Sedaxane_cis	332.1	159.0	15.2	130	10	35	14
		292.0		70	10	25	18
Sedaxane_trans	332.1	159.0	15.2	130	10	35	18
		292.0		40	10	25	6
Sethoxydim	328.1	178.1	16.7	30	10	25	18
		282.2		30	10	20	16
Simetryn	214.1	124.0	13.7	130	10	30	12
		144.0		30	10	30	20
		166.1		30	10	25	20
Spinetoram (J)	748.4	142.1	16.0	20	10	35	18
		98.2		110	10	85	12
Spinetoram (L)	760.4	142.1	16.3	60	10	35	16
		98.2		20	10	95	18
Spinosyn A	732.4	142.1	15.8	30	10	35	18
		98.2		60	10	95	10
Spinosyn D	746.4	142.2	16.0	60	10	40	16
		98.2		40	10	100	12
Spirodiclofen	411.1	313.0	17.3	20	10	20	6
		71.2		20	10	45	6
Spirotetramat-enol	302.1	216.2	9.9	140	10	40	12
		270.2		140	10	30	6
Spiroxamine	298.2	144.1	14.7	100	10	30	14
		100.1		60	10	45	16
		72.1		60	10	65	20
Sulfotep	323.1	171.1	15.8	60	10	20	20
		143.0		60	10	30	20
		115.0		60	10	45	20
Sulprofos	323.1	218.9	17.0	20	10	25	20
		139.2		20	10	45	16
		113.0		20	10	45	6
Tau-fluvalinate	520.2	208.2	17.5	20	10	25	6
		181.0		20	10	50	6
TCMTB	239.0	180.0	15.0	30	10	20	20

		136.0		50	10	40	18
		109.0		90	10	55	12
Tebuconazole	308.1	70.0	15.8	30	10	50	10
		125.1		60	10	55	16
Tebufenpyrad	334.2	117.0	16.6	20	10	50	6
		145.0		20	10	35	18
Tebufloquin	290.1	248.0	16.0	60	10	25	8
		232.1		30	10	60	12
Tebuthiuron	229.1	172.1	13.6	50	10	25	20
		116.2		30	10	35	18
Tepraloxydim	342.1	250.2	15.4	20	10	20	14
		166.2		110	10	30	10
Terbufos-oxon	273.0	103.0	15.4	30	10	15	12
		57.0		30	10	35	6
Terbufos-oxon-sulfone	305.0	231.0	12.7	120	10	20	12
		203.0		120	10	25	12
Terbufos-oxon-sulfoxide	289.0	171.1	12.4	30	10	20	16
		115.0		40	10	40	16
Terbufos-sulfone	321.0	97.0	14.8	150	10	60	12
		171.0		140	10	20	16
Terbufos-sulfoxide	305.0	187.0	14.8	30	10	20	12
		131.0		30	10	40	16
Terbuthylazine	230.1	174.1	15.2	40	10	25	20
		104.1		50	10	50	12
Terbutryn	242.1	186.2	15.4	50	10	25	12
		96.0		80	10	40	10
Tetrachlorvinphos	367.1	126.9	15.7	140	10	20	18
		241.0		10	10	30	18
		206.0		140	10	55	10
Tetraconazole	372.0	159.0	15.4	20	10	50	14
		70.0		20	10	40	16
Thenylchlor	324.1	127.0	15.5	40	10	25	12
		97.1		100	10	60	10
Thiabendazole	202.0	175.1	9.9	30	10	35	10
		131.1		30	10	45	16
Thiacloprid	253.1	126.1	11.6	110	10	30	16
		90.1		110	10	50	10
Thiamethoxam	292.0	211.0	8.5	50	10	20	12
		181.0		30	10	30	20
Thiazopyr	397.1	377.1	15.8	120	10	35	12

		335.1		70	10	40	12
Thidiazuron	221.0	102.0	13.3	50	10	25	12
		128.0		50	10	25	8
Thiobencarb	258.1	125.0	16.2	30	10	35	14
		89.0		40	10	70	10
Thionazin	249.0	192.9	14.4	120	10	20	6
		124.9		60	10	25	12
		96.9		60	10	40	12
Tolfenpyrad	384.2	197.3	16.6	20	10	35	6
		154.2		20	10	55	18
		197.3		20	10	40	6
Triadimefon	294.2	197.1	15.2	30	10	25	6
		69.1		10	10	30	18
Triafamone	407.0	245.0	13.5	60	10	35	16
		160.0		80	10	60	20
Triazamate	315.1	72.0	15.4	30	10	50	12
		226.0		40	10	20	20
Triazophos	314.0	162.0	15.3	60	10	25	12
		119.1		40	10	45	12
Tribufos	315.1	169.0	17.6	20	10	20	18
		113.0		20	10	35	6
Tricyclazole	190.0	163.1	12.2	40	10	35	20
		136.0		10	10	40	16
Trifloxystrobin	409.1	186.1	16.2	40	10	20	16
		206.0		80	10	20	20
Triflumizole	346.0	278.0	16.3	50	10	15	14
		72.9		30	10	25	20
Triflumuron	359.1	156.2	15.9	20	10	25	10
		139.0		20	10	50	18
Triticonazole	318.1	70.0	15.4	20	10	60	20
		125.1		20	10	55	12
TZ-1E	410.1	310.0	15.5	20	10	20	10
		107.1		20	10	30	6
Valifenalate	399.1	155.0	15.2	70	10	55	18
		116.0		70	10	35	6
Vamidothion	288.0	145.9	10.5	80	10	20	18
		118.0		60	10	35	14
Vernolate	204.1	128.0	16.3	70	10	15	16
		86.0		60	10	20	10
XMC	180.1	123.0	13.9	40	10	20	6

		107.0		110	10	55	14
Zoxamide	336.0	187.0	16.0	20	10	35	10
		159.1		20	10	60	8
		189.0		60	10	35	6

DP: Declustering Potential

EP: Entrance Potential

CE: Collision Energy

CXP: Collision Cell Exit Potential

Table S2. MRM condition (12 compounds for negative mode)

Compound name	Precursor ion	Product ion	Retention Time (min)	DP	EP	CE	CXP
6-Benzyl aminopurine	224.0	133.0	12.3	-10	-10	-30	-15
		132.0		-80	-10	-45	-15
Bistrifluron	444.9	262.0	16.9	-20	-10	-30	-5
		186.0		-20	-10	-80	-5
Fipronil	434.8	329.9	15.5	-20	-10	-25	-5
		249.9		-40	-10	-40	-5
Fipronil-sulfone	450.8	415.0	15.7	-90	-10	-25	-13
		282.0		-130	-10	-40	-9
Fluazinam	462.8	415.9	16.5	-70	-10	-30	-13
		398.1		-140	-10	-25	-13
Haloxyfop	359.9	288.0	15.6	-10	-10	-20	-5
		195.9		-10	-10	-60	-19
Hexaflumuron	458.8	174.9	16.2	-20	-10	-45	-5
		276.0		-20	-10	-25	-5
Imazosulfuron	411.0	230.0	15.1	-80	-10	-25	-15
		154.1		-40	-10	-40	-5
Mesotrione	338.0	291.0	11.5	-10	-10	-15	-9
		212.0		-10	-10	-45	-17
Novaluron	490.8	85.0	16.1	-40	-10	-75	-5
		304.9		-20	-10	-20	-5
Teflubenzuron	378.8	338.9	16.6	-10	-10	-15	-7
		195.9		-110	-10	-30	-5
Thifensulfuron-methyl	385.9	139.0	13.2	-150	-10	-40	-15
		107.0		-70	-10	-70	-15

DP: Declustering Potential

EP: Entrance Potential

CE: Collision Energy

CXP: Collision Cell Exit Potential

Table S3. Method validation data for pesticide residue analysis in soybean

No.	Compound name	r^2	Linear range (μg/kg)	Sample preparation method 3 (type 2 d-SPE sorbent)								
				Low (LOQ) (μg/kg)	Recovery %	SD	RSD %	High (μg/kg)	Recovery %	SD	RSD %	Matrix effect %
1	2,3,5-trimethacarb	0.9975	0.1-50	5	71.1	11.0	15.5	50	88.3	1.7	1.9	-27.3
2	3,4,5-trimethacarb	0.9995	0.1-25	1	72.6	11.5	15.8	50	80.4	1.8	2.2	-8.6
3	3-hydroxycarbofuran	0.9983	0.1-25	5	77.4	1.8	2.4	50	79.6	1.9	2.4	1.6
4	6-Benzyl aminopurine	0.9995	0.1-50	5	70.7	6.7	9.5	50	83.0	5.9	7.1	-39.1
5	Acetamiprid	0.9995	0.1-50	5	80.3	5.7	7.1	50	84.8	3.8	4.5	-24.8
6	Acetochlor	0.9990	0.1-50	5	105.3	9.3	8.8	50	89.7	9.5	10.6	-26.6
7	Alachlor	0.9937	0.25-25	5	85.7	16.6	19.4	50	71.0	4.6	6.5	2.9
8	Aldicarb	0.9986	0.1-50	2.5	97.0	16.3	16.8	50	83.9	9.7	11.6	-21.3
9	Alidcarb sulfone	0.9996	0.1-50	2.5	73.1	2.1	2.8	50	81.3	1.4	1.8	-21.8
10	Ametryn	0.9985	0.1-25	5	73.5	6.3	8.5	50	82.9	5.3	6.4	-25.8
11	Anilofos	0.9909	0.1-25	5	81.0	2.6	3.3	50	75.2	2.4	3.3	0.3
12	Aramite	0.9999	0.1-25	2.5	97.2	8.2	10.3	50	93.2	2.6	3.5	-49.0
13	Atrazine	0.9982	0.1-50	5	80.8	13.8	17.1	50	80.7	1.0	1.2	-24.2
14	Azaconazole	0.9984	0.1-50	5	81.7	4.0	4.9	50	89.6	5.7	6.4	-30.3
15	Azamethiphos	0.9972	0.1-50	10	74.2	6.8	9.1	50	98.8	11.0	11.1	-32.1

16	Azimsulfuron	0.9995	0.1-50	5	84.2	5.6	6.7	50	84.4	4.3	5.1	-14.4
17	Azoxystrobin	0.9988	0.1-50	1	103.4	20.0	19.4	50	89.2	15.1	16.9	-15.3
18	Bendiocarb	0.9966	0.1-50	5	75.4	9.6	12.8	50	93.8	1.4	1.5	-33.1
19	Benodanil	0.9982	0.1-50	1	88.5	14.8	16.8	50	80.0	2.1	2.7	-24.3
20	Bensulfuron-methyl	0.9970	0.1-50	5	114.3	2.3	2.0	50	81.6	7.9	9.6	-13.0
21	Benzobicyclon	0.9945	0.1-50	10	79.9	12.5	15.7	50	108.6	1.3	1.2	-36.3
22	Benzoximate	0.9995	0.25-50	5	102.3	6.4	7.7	50	117.1	6.4	6.7	-25.2
23	Benzoylprop-ethyl	0.9975	0.1-25	10	71.1	3.6	5.1	50	70.4	3.7	5.2	1.5
24	Bistrifluron	0.9992	0.1-50	1	117.7	18.3	19.0	50	104.8	7.7	9.0	-42.3
25	Bixafen	0.9912	0.1-25	2.5	114.8	17.5	15.3	50	87.8	13.9	15.8	3.1
26	Bromacil	0.9998	0.1-50	2.5	91.2	11.2	12.2	50	86.2	5.3	6.1	-27.3
27	Bromobutide	0.9993	0.25-50	10	107.5	9.7	11.1	50	106.0	6.3	7.3	-35.0
28	Bupirimate	0.9964	0.1-50	5	77.1	0.9	1.2	50	86.8	15.8	18.2	-26.0
29	Cadusafos	0.9984	0.1-25	10	71.3	13.8	19.4	50	72.9	3.4	4.7	-17.1
30	Carbaryl	0.9991	0.1-50	5	79.5	7.1	8.9	50	91.3	5.1	5.6	-32.7
31	Carbendazim	0.9989	0.25-50	10	91.8	8.5	11.4	50	108.9	SD	3.8	-36.4
32	Carbetamide	0.9985	0.1-50	2.5	79.6	7.5	9.4	50	89.3	4.6	5.1	-18.8
33	Carbofuran	0.9951	0.1-50	10	71.3	10.0	14.1	50	91.9	3.0	3.2	-35.0
34	Carbofuran-3-hydroxy	0.9959	0.1-50	10	72.3	8.9	12.4	50	88.4	1.5	1.8	-24.3

35	Carboxin	0.9992	0.1-50	5	75.5	0.1	0.1	50	76.7	7.3	9.5	-25.4
36	Carpropamid	0.9964	0.1-50	10	71.2	9.1	12.8	50	75.5	7.8	10.3	-24.3
37	Chlorantraniliprole	0.9991	0.25-50	5	107.0	16.4	15.3	50	81.3	3.4	4.1	-33.3
38	Chlorfenvinphos	0.9937	0.1-25	2.5	95.1	11.4	12.0	50	79.5	5.5	6.9	-7.2
39	Chlorfluazuron	0.9988	0.1-25	2.5	112.8	12.8	13.9	50	89.5	4.6	6.3	-56.0
40	Chloridazon	0.9992	0.1-50	1	74.2	3.3	4.4	50	83.0	1.9	2.3	-27.6
41	Chlorimuron-ethyl	0.9976	0.1-50	2.5	115.9	16.0	13.8	50	76.3	4.9	6.4	-27.8
42	Chlorotoluron	0.9989	0.1-25	2.5	77.7	8.2	10.6	50	91.9	4.7	5.1	-18.5
43	Chloroxuron	0.9977	0.1-25	10	81.1	5.7	7.0	50	82.4	8.3	10.0	-7.5
44	Chlorsulfuron	0.9933	0.1-25	10	78.1	7.8	10.0	50	73.1	14.2	19.5	33.4
45	Chromafenozide	0.9967	0.1-50	5	80.6	10.3	12.8	50	78.6	14.0	17.9	-23.6
46	Clofentezine	0.9992	0.1-25	5	101.1	9.9	12.0	50	79.7	4.7	7.2	-42.0
47	Clomazone	0.9982	0.1-50	5	79.0	4.6	5.8	50	85.4	6.9	8.1	-30.7
48	Clothianidin	0.9989	0.1-50	5	76.4	7.7	10.1	50	84.0	6.3	7.5	-32.8
49	Coumaphos	0.9974	0.1-50	5	108.8	10.1	11.3	50	118.9	11.5	11.9	-35.8
50	Crotoxyphos	0.9968	0.1-25	10	77.5	11.3	14.6	50	87.7	14.6	16.6	-11.5
51	Crufomate	0.9979	0.1-50	10	83.4	11.1	13.4	50	85.2	1.7	2.0	-18.7
52	Cyanazine	0.9996	0.1-50	2.5	86.2	9.2	10.6	50	83.9	3.7	4.5	-14.1
53	Cyantraniliprole	0.9989	0.1-50	2.5	98.5	12.5	12.7	50	75.9	0.6	0.8	-27.4

54	Cyazofamid	0.9953	0.1-50	10	78.8	8.6	10.9	50	82.9	5.8	7.0	-26.0
55	Cyclosulfamuron	0.9912	0.1-50	10	70.7	5.6	7.9	50	96.6	5.3	5.5	-28.2
56	Cyflufenamid	0.9924	0.1-25	5	85.0	8.2	9.6	50	71.9	6.3	8.7	6.7
57	Cymoxanil	0.9979	0.1-50	10	75.6	10.0	13.2	50	90.0	5.2	5.8	-32.4
58	Daimuron	0.9978	0.1-50	10	73.0	7.9	10.8	50	85.0	5.3	6.2	-28.8
59	Demeton-O	0.9905	0.1-50	5	106.8	18.1	16.9	50	87.0	9.1	10.4	-28.1
60	Demeton-S-methyl	0.9982	0.1-25	2.5	95.7	17.3	18.1	50	90.0	9.8	10.9	8.2
61	Demeton-S-methyl sulfoxide	0.9986	0.1-25	5	73.9	0.9	1.2	50	70.1	3.3	4.7	4.5
62	Demeton-S-methyl-sulfone	0.9992	0.1-50	2.5	72.0	5.2	7.2	50	83.2	2.5	3.0	-21.5
63	Demeton-S-sulfone	0.9994	0.1-50	2.5	71.2	1.0	1.4	50	89.0	1.9	2.1	-25.1
64	Demeton-S-sulfoxide	0.9998	0.1-50	1	79.3	12.3	15.5	50	82.6	2.3	2.8	-31.3
65	Desmetryn	0.9972	0.1-50	10	81.3	2.0	2.5	50	83.2	5.2	6.2	-26.9
66	Diazinon	0.9985	0.25-50	10	91.0	10.0	13.4	50	104.6	1.8	2.1	-42.2
67	Dicrotophos	0.9989	0.1-50	5	73.8	2.1	2.9	50	80.3	0.6	0.7	-25.3
68	Diethatyl-ethyl	0.9969	0.1-50	5	72.6	6.1	8.4	50	86.5	4.6	5.3	-29.0
69	Diethofencarb	0.9979	0.1-50	10	87.0	6.2	7.1	50	88.6	11.4	12.8	-34.9
70	Dimepiperate	0.9968	0.1-25	5	81.5	11.1	16.6	50	79.1	11.5	17.8	-49.9
71	Dimethachlor	0.9981	0.1-50	5	71.8	1.7	2.4	50	88.2	2.8	3.2	-34.7
72	Dimethametryn	1.0000	0.25-50	10	105.0	16.0	18.6	50	102.3	4.9	5.9	-35.8

73	Dimethenamide	0.9975	0.1-50	10	84.1	13.5	16.1	50	83.9	3.4	4.1	-25.8
74	Dimethoate	0.9976	0.1-50	5	74.2	4.4	6.0	50	85.5	3.9	4.5	-26.0
75	Dimethylaminosulfotoluidide (DMST)	0.9994	0.1-50	5	91.4	3.9	4.2	50	88.5	5.7	6.4	-12.2
76	Diniconazole	0.9922	0.1-50	5	110.5	18.0	16.3	50	71.0	9.7	13.6	-27.5
77	Diphenamid	0.9973	0.1-50	5	85.4	8.4	9.9	50	86.9	2.8	3.2	-36.9
78	Disulfoton-sulfone	0.9916	0.1-25	5	71.4	6.8	9.5	50	85.6	12.5	14.6	-7.4
79	Disulfoton-sulfoxide	0.9977	0.1-50	10	79.9	15.5	19.4	50	98.6	9.9	10.0	-33.2
80	Dithiopyr	0.9992	0.1-50	2.5	81.1	7.5	9.2	50	70.7	2.4	3.3	-22.7
81	Diuron	0.9987	0.1-50	5	79.9	4.7	5.9	50	89.9	4.9	5.4	-28.6
82	Edifenphos	0.9949	0.1-25	10	94.0	9.6	12.5	50	83.7	SD	1.4	0.0
83	Emamectin B1a	0.9995	0.1-50	1	91.5	13.1	14.3	50	74.3	9.9	13.3	-14.7
84	Ethaboxam	0.9977	0.1-25	5	80.8	8.6	10.7	50	90.9	7.5	8.2	21.9
85	Ethiofencarb	0.9994	0.1-25	10	80.8	9.4	11.7	50	85.8	9.2	10.7	-11.3
86	Ethirimol	0.9993	0.1-50	10	71.3	8.0	11.2	50	75.1	3.7	4.9	-28.8
87	Ethoprophos	0.9997	0.1-50	10	83.6	9.4	11.3	50	81.2	1.6	2.0	-18.6
88	Ethoxysulfuron	0.9986	0.1-50	5	100.4	15.2	15.1	50	80.4	13.3	16.6	-19.3
89	Etoxazole	0.9995	0.1-25	2.5	98.7	4.3	5.3	50	76.9	SD	10.7	-51.1
90	Etrimfos	0.9986	0.25-50	10	118.2	14.3	14.9	50	93.3	6.4	8.5	-39.0

91	Famoxadone	0.9905	0.1-25	5	96.8	19.3	19.9	50	92.1	11.7	12.7	8.9
92	Fenamiphos	0.9958	0.1-50	5	71.8	2.6	3.6	50	86.6	9.4	10.8	-22.6
93	Fenamiphos sulfoxide	0.9993	0.1-50	2.5	99.4	3.6	3.6	50	93.0	2.2	2.4	-22.2
94	Fenamiphos-sulfone	0.9980	0.1-50	5	83.5	9.0	10.8	50	96.1	4.0	4.2	-29.2
95	Fenamiphos-sulfoxide	0.9985	0.1-50	5	72.4	1.4	1.9	50	90.4	5.4	6.0	-24.4
96	Fenfuram	0.9951	0.1-25	1	80.1	8.3	10.4	50	82.9	5.1	6.2	-8.8
97	Fenobucarb	0.9988	0.1-50	10	76.0	6.3	8.3	50	90.7	14.2	15.6	-36.1
98	Fenothiocarb	0.9987	0.1-50	5	112.8	SD	16.0	50	116.0	SD	11.2	-45.4
99	Fenoxanil	0.9954	0.1-50	5	80.8	10.7	13.3	50	72.5	4.6	6.3	-4.5
100	Fenoxaprop-ethyl	0.9996	0.1-25	10	108.2	11.8	13.4	50	107.9	SD	2.1	-45.7
101	Fenpyroximate	0.9995	0.1-25	2.5	115.6	0.7	0.8	50	95.7	SD	12.3	-50.0
102	Fenquinotrione_KIH-3635-M-2	0.9985	0.1-25	10	110.4	18.0	20.0	50	99.9	SD	17.1	-35.8
103	Fensulfothion	0.9965	0.1-50	2.5	91.5	16.8	18.4	50	87.2	5.2	5.9	-15.1
104	Fenthion oxon	0.9953	0.1-50	10	82.3	15.6	19.0	50	94.1	5.4	5.8	-34.9
105	Fenthion oxon sulfone	0.9995	0.1-50	2.5	75.4	2.8	3.7	50	94.5	5.7	6.0	-30.7
106	Fenthion oxon sulfoxide	0.9997	0.1-50	5	81.6	6.2	7.6	50	86.6	4.7	5.4	-26.2
107	Fenthion sulfoxide	0.9983	0.1-50	5	83.5	3.7	4.4	50	94.7	9.6	10.1	-37.5
108	Fenthion-sulfone	0.9924	0.1-25	5	80.9	15.4	19.0	50	81.5	4.6	5.6	-12.3
109	Ferimzone	0.9970	1-50	10	70.7	3.8	5.4	50	91.4	6.9	7.6	-36.6

110	Fipronil	0.9904	0.1-50	10	77.1	11.6	15.1	50	79.9	4.2	5.3	-6.5
111	Fipronil-sulfone	0.9972	0.1-50	10	73.7	9.5	12.9	50	91.6	4.6	5.1	-26.8
112	Flamprop-isopropyl	0.9993	0.1-50	10	80.6	12.6	15.6	50	84.2	1.9	2.3	-19.5
113	Flonicamid	0.9997	0.1-50	5	77.6	2.2	2.8	50	88.4	4.0	4.6	-24.5
114	Fluacrypyrim	0.9971	0.1-25	2.5	98.6	14.2	14.4	50	78.9	9.5	12.0	18.5
115	Flucetosulfuron	0.9979	0.1-50	10	75.7	14.4	19.0	50	87.9	7.9	9.0	-30.6
116	Flufenacet	0.9984	0.1-50	5	76.4	7.3	9.6	50	82.8	3.3	3.9	-1.9
117	Flufenoxuron	0.9908	0.1-50	10	119.4	18.1	18.5	50	95.4	7.3	9.3	-34.7
118	Fluometuron	0.9981	0.1-25	5	108.4	12.5	11.5	50	75.1	9.5	12.7	-0.6
119	Fluopicolide	0.9959	0.1-50	10	74.0	9.1	12.3	50	84.3	7.9	9.4	-32.3
120	Fluopyram	0.9956	0.1-50	10	73.1	5.5	7.5	50	95.8	0.8	0.9	-28.7
121	Flupyradifurone	0.9993	0.1-50	5	85.1	7.8	9.1	50	84.1	2.7	3.2	0.1
122	Fluridone	0.9937	0.1-50	10	94.7	3.2	3.4	50	92.0	1.1	1.1	-37.1
123	Flusilazole	0.9950	0.1-25	5	84.1	7.6	9.0	50	93.4	6.4	6.9	-5.0
124	Flutianil	0.9984	0.1-50	5	71.2	10.3	14.5	50	84.3	1.3	1.5	-30.6
125	Flutolanil	0.9983	0.1-25	10	78.3	7.9	10.1	50	85.6	7.1	8.3	-7.6
126	Flutriafol	0.9982	0.1-50	5	108.4	4.9	4.5	50	79.1	8.1	10.2	-10.2
127	Fluxapyroxad	0.9992	0.1-25	1	76.8	10.0	13.0	50	95.6	6.1	6.3	30.0
128	Forchlorfenuron	0.9966	0.1-50	10	82.5	5.6	6.7	50	88.0	6.0	6.8	-20.2

129	Fosthiazate	0.9980	0.1-50	5	85.8	9.2	10.8	50	83.8	9.5	11.3	-24.2
130	Furathiocarb	0.9985	0.1-25	10	116.5	16.2	17.1	50	113.4	3.6	3.9	-46.8
131	Heptenophos	0.9972	0.1-50	10	81.6	5.5	6.7	50	88.4	7.3	8.2	-27.4
132	Hexazinone	0.9980	0.1-50	5	70.6	4.9	7.0	50	92.2	4.9	5.3	-28.8
133	Imazalil	0.9992	0.1-25	10	106.9	6.1	7.0	50	111.2	5.3	5.8	-36.4
134	Imazapic	0.9996	0.1-25	2.5	112.1	9.6	10.5	50	73.5	5.0	8.3	-31.5
135	Imazaquin	0.9997	0.1-25	5	117.6	SD	10.4	50	104.8	SD	1.5	-42.1
136	Imazethapyr	0.9993	0.1-25	5	111.7	5.8	6.3	50	93.7	5.9	7.7	-31.3
137	Imazosulfuron	0.9951	0.1-25	2.5	84.3	2.5	2.9	50	79.0	15.7	19.8	62.6
138	Imicyafos	0.9980	0.1-50	2.5	75.5	5.4	7.2	50	87.9	7.2	8.2	-28.5
139	Imidacloprid	0.9991	0.1-50	5	70.5	3.4	4.9	50	86.9	5.2	6.0	-7.6
140	Inabenfide	0.9983	0.1-50	5	74.0	7.3	9.9	50	86.4	3.5	4.1	-36.5
141	Indanofan	0.9931	0.1-50	10	74.7	4.1	5.5	50	80.1	11.7	14.6	-29.8
142	Indoxacarb	0.9989	0.1-50	10	73.1	8.6	11.7	50	79.0	11.0	13.9	-15.4
143	Iprobenfos	0.9949	0.5-25	5	73.7	13.8	18.7	50	81.4	3.0	3.7	-3.0
144	Iprovalicarb	0.9999	0.1-50	5	76.6	8.6	11.3	50	94.7	5.4	5.7	-30.3
145	Isoprocarb	0.9995	0.25-50	2.5	89.9	9.8	10.9	50	84.9	3.7	4.3	-25.1
146	Isoproturon	0.9966	0.1-50	5	73.5	11.7	15.9	50	90.7	4.7	5.2	-24.1
147	Isopyrazam	0.9994	0.1-25	2.5	72.2	8.4	11.6	50	72.8	8.0	11.0	-9.3

148	Isoxaben	0.9972	0.1-50	5	72.4	9.6	13.2	50	94.4	1.5	1.6	-24.2
149	Isoxathion	0.9990	0.1-25	5	94.9	8.3	10.7	50	97.7	8.7	11.0	-46.6
150	Malaoxon	0.9995	0.1-50	1	78.0	4.2	5.4	50	87.4	5.8	6.6	-21.4
151	Mandestrobin	0.9931	0.1-50	10	75.5	11.4	15.1	50	87.5	4.2	4.8	-30.0
152	Mandipropamid	0.9979	0.1-50	1	88.8	7.7	8.7	50	90.3	11.9	13.2	-38.1
153	Mefenacet	0.9990	0.1-50	10	70.1	12.9	18.3	50	80.4	4.7	5.8	-28.6
154	Mefentrifluconazole	0.9954	0.1-25	5	75.1	14.1	18.7	50	84.0	11.5	13.7	13.4
155	Mepanipyrim	0.9978	0.1-25	5	118.5	6.6	6.9	50	99.2	9.0	11.1	-33.7
156	Mephosfolan	0.9998	0.1-50	1	75.0	8.5	11.3	50	84.7	3.3	3.8	-27.0
157	Mepronil	0.9985	0.1-25	10	73.8	11.9	16.1	50	86.1	8.5	9.9	-6.5
158	Metaflumizone (E)	0.9741	1-50	10	76.2	5.1	6.7	50	78.1	12.4	15.8	-19.1
159	Metalaxyl	0.9993	0.1-50	2.5	109.5	11.8	10.7	50	92.1	5.4	5.9	-21.6
160	Metamifop	0.9974	0.1-50	2.5	100.0	3.3	3.3	50	77.0	3.5	4.5	-29.1
161	Metamitron	0.9986	0.5-25	5	77.0	8.8	11.4	50	79.8	2.3	2.9	-16.4
162	Metconazole	0.9992	0.1-50	10	70.8	10.9	15.3	50	78.9	4.6	5.8	-28.3
163	Methabenzthiazuron	0.9983	0.1-50	5	82.6	7.5	9.1	50	81.4	6.1	7.5	-25.7
164	Methamidophos	1.0000	0.1-25	2.5	100.5	1.7	2.0	50	76.7	SD	3.9	92.2
165	Methiocarb	0.9962	0.5-50	5	86.2	10.8	12.6	50	76.9	6.3	8.1	-24.6
166	Methiocarb-sulfone	0.9982	0.1-50	5	74.8	5.4	7.3	50	86.2	1.9	2.2	-37.6

167	Methiocarb-sulfoxide	0.9995	0.1-50	10	75.6	7.6	10.1	50	80.0	3.2	4.1	-19.9
168	Methomyl	0.9994	0.1-50	2.5	112.6	3.2	2.9	50	117.0	5.2	4.5	-24.0
169	Methoprotryne	0.9997	0.1-50	5	80.8	1.2	1.5	50	79.9	4.0	4.9	-38.0
170	Methoxyfenozide	0.9853	1-50	10	103.3	18.7	18.1	50	72.4	8.8	12.2	-31.6
171	Metobromuron	0.9983	0.1-25	2.5	77.5	7.8	10.0	50	72.2	3.7	5.2	-15.3
172	Metolcarb	0.9992	0.1-50	1	101.9	15.7	15.4	50	80.4	2.6	3.2	-25.5
173	Metominostrboin (Z)	0.9980	0.1-50	5	106.9	5.6	5.3	50	87.3	5.2	5.9	-21.7
174	Metominostrobin (E)	0.9975	0.1-50	10	78.1	6.6	8.5	50	85.8	10.2	11.9	-25.3
175	Metrafenon	0.9976	0.25-50	10	98.2	7.1	8.9	50	96.0	5.0	6.3	-42.9
176	Metrafenone	0.9986	0.25-50	10	105.6	3.8	4.4	50	103.4	SD	3.1	-40.3
177	Mevinphos	0.9997	0.5-50	10	72.6	5.1	7.0	50	86.6	2.1	2.4	-35.2
178	Monocrotophos	0.9997	0.25-50	2.5	77.1	5.2	6.8	50	73.9	3.7	4.9	-25.6
179	Monolinuron	0.9996	0.1-50	5	71.3	9.5	13.3	50	83.1	4.0	4.8	-24.8
180	Nicosulfuron	0.9991	0.1-25	2.5	101.9	10.5	10.3	50	81.7	3.5	4.2	43.0
181	Nitenpyram	0.9999	0.5-50	10	102.2	8.7	10.4	50	95.9	SD	2.5	-31.9
182	Norflurazon	0.9984	0.1-50	10	88.5	11.0	12.5	50	92.4	4.0	4.3	-29.3
183	Novaluron	0.9925	0.1-25	10	71.1	12.4	17.4	50	79.4	15.8	19.9	26.5
184	Nuarimol	0.9987	0.1-50	5	84.7	14.2	16.8	50	87.9	14.7	16.7	-23.6
185	Ofurace	0.9979	0.1-50	5	70.6	2.4	3.4	50	87.3	4.3	5.0	-31.0

186	Oxadiazon	0.9975	0.1-25	2.5	91.8	6.5	8.7	50	82.4	4.5	6.6	-44.5
187	Oxadixyl	0.9982	0.1-50	5	81.3	5.2	6.4	50	85.0	5.9	6.9	-28.4
188	Oxamyl	0.9997	0.1-50	5	72.5	1.8	2.5	50	80.5	6.2	7.7	-23.1
189	Oxathiapiprolin	0.9996	0.1-50	5	107.8	5.3	4.9	50	89.3	4.4	5.0	-24.0
190	Oxaziclomefone	0.9997	0.1-25	2.5	113.1	8.9	9.7	50	86.5	SD	7.8	-47.1
191	Oxycarboxin	0.9949	0.1-50	10	79.0	7.6	9.6	50	89.2	5.4	6.1	-12.4
192	Oxydemeton-methyl	0.9990	0.1-25	2.5	71.8	2.4	3.4	50	71.9	4.1	5.7	3.4
193	Paraoxon-methyl	0.9996	0.25-50	10	78.9	3.8	4.8	50	91.2	7.2	7.9	-28.5
194	Penconazole	0.9969	0.1-50	10	91.2	14.6	16.0	50	84.7	11.6	13.7	-32.0
195	Penoxsulam	0.9992	0.1-50	10	74.4	13.7	18.4	50	98.7	7.7	7.8	-13.8
196	Phenthoate	0.9989	0.1-25	5	119.9	16.8	17.2	50	91.2	13.3	17.9	-30.0
197	Phorate	0.9994	0.1-50	10	76.9	10.9	14.2	50	81.5	4.9	6.0	-23.9
198	Phorate-oxon	0.9970	0.1-50	10	72.4	6.1	8.4	50	99.8	6.6	6.6	-43.0
199	Phorate-oxon-sulfone	0.9993	0.1-50	5	83.8	3.6	4.3	50	88.9	1.1	1.3	-26.6
200	Phorate-oxon-sulfoxide	0.9993	0.1-50	5	80.8	2.8	3.5	50	79.3	3.9	4.9	-24.1
201	Phorate-sulfoxide	0.9975	0.1-50	10	76.0	14.2	18.7	50	93.6	2.6	2.8	-35.0
202	Phosalone	0.9992	0.1-25	10	101.1	12.0	14.5	50	93.5	SD	2.8	-39.0
203	Phosfolan	0.9991	0.1-50	5	79.3	2.9	3.7	50	84.8	0.9	1.1	-24.5
204	Phosmet-oxon	0.9990	0.1-50	5	72.9	3.0	4.2	50	89.0	4.9	5.5	-25.1

205	Phosphamidon	0.9986	0.1-50	2.5	78.0	10.1	13.0	50	90.0	8.4	9.3	-30.4
206	Phoxim	0.9987	1-25	5	90.8	SD	18.6	50	115.9	SD	4.1	-54.2
207	Picolinafen	0.9999	0.1-25	2.5	104.8	10.1	11.8	50	89.8	SD	2.3	-43.8
208	Picoxystrobin	0.9988	0.1-25	2.5	85.7	11.6	13.6	50	72.3	10.2	14.2	5.9
209	Pinoxaden	0.9987	0.1-25	1	98.2	5.8	5.9	50	71.1	9.7	13.7	27.9
210	Piperonyl butoxide	0.9997	0.1-25	2.5	96.7	7.6	9.6	50	98.0	1.0	1.3	-49.7
211	Pirimicarb	0.9993	0.1-50	5	73.4	2.2	3.0	50	86.5	5.2	6.1	-34.1
212	Pirimicarb-desmethyl	0.9991	0.1-50	5	73.9	3.3	4.5	50	82.1	2.6	3.1	-22.9
213	Pirimiphos-ethyl	0.9999	0.1-25	2.5	104.9	8.7	10.1	50	72.0	3.0	5.1	-43.9
214	Pirimiphos-methyl	0.9992	0.1-25	2.5	113.0	9.8	10.7	50	100.9	SD	13.0	-53.0
215	Probenazole	0.9997	0.5-50	5	83.2	11.2	13.5	50	82.0	2.0	2.5	-23.2
216	Prochloraz	0.9927	0.1-25	2.5	111.5	18.1	16.3	50	73.8	5.1	6.9	8.0
217	Profenofos	0.9991	0.1-25	5	94.9	5.3	6.9	50	80.8	4.5	6.8	-38.0
218	Promecarb	0.9987	0.1-50	5	75.4	12.3	16.4	50	87.0	5.7	6.5	-31.2
219	Prometryn	0.9951	0.1-25	10	106.9	15.0	17.1	50	82.7	6.0	8.9	-19.7
220	Pronamide (Propyzamide)	0.9973	0.1-50	10	70.7	3.4	4.8	50	89.7	7.3	8.2	-25.9
221	Propachlor	0.9971	0.25-25	5	93.0	4.0	4.3	50	70.0	8.1	11.6	24.0
222	Propaquizafop	0.9990	0.1-25	2.5	72.1	3.5	4.9	50	70.3	2.5	3.6	3.1
223	Propargite	0.9994	0.1-25	2.5	95.2	3.9	5.0	50	72.6	4.0	6.7	-47.5

224	Propazine	0.9997	0.1-50	10	74.1	7.8	10.5	50	77.2	1.0	1.3	-27.5
225	Propoxur	0.9984	0.1-50	5	80.0	3.1	3.9	50	89.3	3.6	4.0	-26.2
226	Prosulfocarb	0.9996	0.5-50	5	75.3	7.1	11.5	50	70.9	1.3	2.3	-41.8
227	Prothioconazole-desthio	0.9937	0.1-50	10	72.0	7.4	10.2	50	78.7	7.9	10.0	-26.5
228	Pydiflumetofen	0.9982	0.1-25	2.5	99.0	6.3	6.3	50	79.0	5.4	6.8	-10.1
229	Pyflubumide	0.9925	0.1-50	2.5	91.0	3.7	4.1	50	79.9	3.8	4.7	-24.1
230	Pyflubumide-NH	0.9974	0.1-50	10	87.9	11.8	13.4	50	93.3	0.7	0.8	-24.7
231	Pyracarbolid	0.9994	0.1-50	10	75.1	2.6	3.5	50	86.8	10.6	12.3	-30.9
232	Pyraclofos	0.9957	0.1-25	5	78.2	6.4	8.2	50	78.9	5.0	6.3	-1.9
233	Pyraclonil	0.9973	0.1-50	10	81.2	9.6	11.8	50	91.1	8.0	8.8	-22.8
234	Pyraclostrobin	0.9994	0.1-50	5	82.5	15.4	18.7	50	77.8	4.2	5.5	-27.3
235	Pyraziflumid	0.9990	0.1-50	10	76.6	11.1	14.4	50	70.2	12.4	17.7	-18.7
236	Pyrazophos	0.9990	0.1-25	5	74.4	6.3	8.5	50	80.9	3.0	3.7	9.3
237	Pyribencarb	0.9995	0.1-50	5	71.3	5.7	8.0	50	89.7	4.1	4.6	-38.8
238	Pyridaphenthion	0.9968	0.1-50	2.5	84.2	5.2	6.2	50	93.4	5.7	6.1	-23.9
239	Pyrifluquinazon	0.9946	0.1-50	5	71.1	9.2	12.9	50	102.3	9.1	8.9	-31.3
240	Pyriftalid	0.9944	0.1-50	5	71.7	8.4	11.7	50	91.9	1.1	1.2	-32.8
241	Pyrimisulfan	0.9998	0.1-50	2.5	82.1	3.2	3.9	50	86.8	3.9	4.5	-10.9
242	Pyroquilon	0.9992	0.1-50	5	71.7	1.0	1.4	50	83.9	5.1	6.1	-33.6

243	Quinoclamine	0.9974	1-25	10	99.6	13.2	16.2	50	109.2	SD	2.3	-43.3
244	Quizalofop-ethyl	0.9995	0.1-25	5	110.0	9.2	10.3	50	94.2	2.2	2.9	-51.3
245	Saflufenacil	0.9970	0.1-25	10	101.5	9.0	8.8	50	87.5	5.2	6.0	38.5
246	Secbumeton	0.9935	0.1-50	5	118.9	2.6	2.2	50	70.2	1.3	1.9	-23.2
247	Sedaxane_cis	0.9914	0.1-25	5	84.7	15.9	18.8	50	85.2	2.1	2.5	-0.7
248	Sedaxane_trans	0.9985	0.1-50	5	88.7	6.1	6.9	50	102.5	8.9	8.6	-34.6
249	Simetryn	0.9999	0.1-50	5	77.6	4.5	5.9	50	81.4	5.8	7.1	-32.6
250	Spinetoram (L)	0.9947	0.25-50	5	76.3	14.7	19.3	50	73.0	7.1	9.7	-29.6
251	Spirotetramat-enol	0.9999	2.5-50	10	118.3	18.2	18.9	50	112.1	16.7	18.2	-34.2
252	Spiroxamine	0.9979	0.25-50	10	103.0	2.4	2.9	50	98.9	7.5	9.3	-53.4
253	Tebuconazole	0.9986	0.1-25	10	84.2	16.1	19.1	50	81.8	13.2	16.1	-18.9
254	Tebufenpyrad	0.9993	0.5-50	5	114.3	SD	2.5	50	90.5	SD	11.7	-40.5
255	Tebufloquin	0.9998	0.1-50	1	88.5	11.7	13.2	50	73.4	7.2	9.8	-22.3
256	Tebuthiuron	0.9971	0.1-50	2.5	72.5	6.7	9.2	50	81.3	3.2	4.0	-38.4
257	Terbufos-oxon	0.9922	0.25-25	5	79.6	14.0	17.5	50	80.7	10.8	13.4	7.3
258	Terbufos-oxon-sulfone	0.9985	0.1-50	5	81.0	3.2	4.0	50	90.4	3.1	3.5	-29.3
259	Terbufos-oxon-sulfoxide	0.9985	0.1-50	5	77.5	5.2	6.8	50	89.3	5.6	6.3	-28.8
260	Terbuthylazine	0.9963	0.1-25	5	74.8	9.1	12.1	50	72.5	2.9	4.0	-3.2
261	Terbutryn	0.9994	0.25-50	10	99.6	6.8	8.3	50	102.4	SD	7.1	-43.0

262	Tetrachlorvinphos	0.9983	0.1-50	5	89.9	15.9	17.7	50	86.0	4.2	4.9	-19.6
263	Tetraconazole	0.9950	0.1-50	10	87.3	6.7	7.7	50	86.7	3.9	4.5	-24.5
264	Thenylchlor	0.9982	0.1-50	10	85.4	10.6	12.4	50	84.7	13.0	15.3	-22.9
265	Thiabendazole	0.9995	0.1-50	5	77.2	1.7	2.2	50	75.7	4.4	5.8	-28.8
266	Thiacloprid	0.9983	0.1-50	5	71.0	1.4	2.0	50	87.8	4.6	5.2	-28.8
267	Thiamethoxam	0.9993	0.1-50	5	77.0	1.6	2.0	50	81.7	4.5	5.5	-19.7
268	Thiazopyr	0.9992	0.1-25	2.5	80.5	9.2	11.4	50	74.6	0.6	0.9	0.5
269	Thidiazuron	0.9996	0.1-50	2.5	80.7	5.3	6.5	50	75.1	2.2	2.9	-14.2
270	Thidiazuron	0.9972	0.1-50	5	84.7	0.9	1.1	50	72.5	9.4	13.0	-10.6
271	Thionazin	0.9981	0.1-25	5	108.0	SD	13.0	50	106.6	SD	6.2	-32.4
272	Tolfenpyrad	0.9998	0.5-25	10	102.3	7.3	8.7	50	90.2	SD	4.2	-46.4
273	Triadimefon	0.9971	0.1-50	10	77.6	12.3	15.8	50	89.0	9.6	10.8	-32.6
274	Triafamone	0.9998	0.1-50	2.5	73.4	2.5	3.4	50	90.3	0.6	0.6	-34.2
275	Triazamate	0.9950	0.1-25	2.5	97.3	7.8	8.0	50	73.2	5.1	7.0	1.7
276	Triazophos	0.9960	0.1-25	2.5	88.2	15.1	17.1	50	74.2	4.1	5.5	0.6
277	Trifloxystrobin	0.9980	0.1-50	10	71.7	11.4	15.9	50	76.7	4.0	5.3	-35.2
278	Triticonazole	0.9978	0.1-50	2.5	75.7	1.9	2.5	50	79.9	6.2	7.7	-22.7
279	TZ-1E	0.9954	0.1-25	2.5	84.1	7.5	8.9	50	74.5	7.6	10.2	14.6
280	Valifenalate	0.9972	0.1-50	5	72.9	6.0	8.3	50	91.8	1.3	1.4	-21.1

281	Vamidothion	0.9991	0.1-50	5	73.9	2.3	3.2	50	82.5	4.0	4.9	-23.2
282	Vernolate	0.9991	0.25-50	5	118.8	14.4	14.9	50	75.9	5.3	8.5	-39.3
283	XMC	0.9995	0.1-50	2.5	78.9	5.0	6.3	50	79.6	1.3	1.6	-29.9
284	Zoxamide	0.9994	0.1-25	10	103.8	7.0	8.3	50	102.9	5.4	6.5	-33.9

SD: Standard Deviation

RSD: Relative Standard Deviation

Table S4. Method validation data for pesticide residue analysis in kidney bean

No.	Compound name	r^2	Linear range (μg/kg)	Sample preparation method 3 (type 3 d-SPE sorbent)								
				Low (LOQ) (μg/kg)	Recovery %	SD	RSD %	High (μg/kg)	Recovery %	SD	RSD %	Matrix effect %
1	2,3,5-trimethacarb	0.9992	0.1-25	1	109.4	7.0	6.4	50	80.3	11.7	14.6	-5.3
2	3,4,5-trimethacarb	0.9995	0.1-25	1	96.2	18.2	18.9	50	76.3	7.7	10.1	3.2
3	3-hydroxycarbofuran	0.9997	0.1-25	2.5	86.1	8.3	9.6	50	86.3	3.3	3.9	-11.2
4	6-Benzyl aminopurine	0.9999	0.1-25	1	101.7	8.0	7.9	50	83.1	2.2	2.7	-2.2
5	Acetamiprid	0.9995	0.1-25	2.5	109.2	5.0	4.6	50	97.0	1.4	1.5	-6.6
6	Acetochlor	0.9970	0.1-25	5	98.9	14.4	14.6	50	85.8	4.1	4.8	7.3
7	Alachlor	0.9983	0.1-25	2.5	110.5	19.1	17.3	50	90.3	2.9	3.3	-3.9
8	Aldicarb	0.9995	0.1-25	2.5	95.1	11.2	11.8	50	94.6	4.6	4.8	0.6
9	Alidcarb sulfone	0.9998	0.1-25	2.5	102.2	3.9	3.8	50	90.6	2.6	2.9	0.0
10	Ametoctradin	0.9962	0.1-25	2.5	117.6	12.0	10.2	50	89.8	8.8	9.9	-13.1
11	Ametryn	0.9900	0.1-25	10	97.0	5.3	5.5	50	105.6	6.1	5.8	-30.3
12	Anilofos	0.9994	0.1-25	2.5	103.0	18.5	18.0	50	95.6	7.1	7.4	-0.8
13	Aramite	0.9980	0.1-25	2.5	80.7	13.2	16.4	50	89.1	4.7	5.2	-16.2
14	Aspon	0.9998	0.1-25	2.5	103.4	1.8	1.8	50	82.6	1.7	2.0	-22.8
15	Atrazine	0.9984	0.1-25	1	114.9	13.9	12.1	50	87.7	9.8	11.1	24.5

16	Azaconazole	0.9991	0.1-25	2.5	100.7	12.9	12.8	50	98.7	1.1	1.1	-7.4
17	Azamethiphos	0.9993	0.1-25	2.5	111.0	6.2	5.6	50	91.3	1.6	1.8	0.3
18	Azimsulfuron	0.9986	0.1-25	10	98.7	14.2	14.4	50	112.4	16.3	14.5	-25.6
19	Azinphos-methyl	0.9976	0.1-25	5	100.1	12.5	12.5	50	106.7	16.8	15.8	-14.7
20	Azoxystrobin	0.9997	0.1-25	5	97.3	8.4	8.6	50	98.9	1.6	1.6	-10.6
21	Benalaxyl	0.9999	0.1-25	1	107.2	2.4	2.2	50	100.6	3.0	2.9	-9.7
22	Bendiocarb	0.9991	0.1-25	2.5	111.0	6.6	6.0	50	93.7	2.3	2.5	-7.7
23	Benfuracarb	0.9961	0.25-50	10	115.5	8.8	7.6	50	81.4	15.9	19.5	-10.8
24	Benodanil	0.9990	0.1-25	5	89.5	3.6	4.0	50	99.1	10.3	10.4	-8.6
25	Benoxacor	0.9994	0.1-25	10	95.0	18.6	19.6	50	91.9	10.3	11.2	-5.8
26	Bensulfuron-methyl	0.9979	0.1-25	10	103.4	13.4	13.0	50	97.1	1.7	1.7	8.0
27	Benthiavalicarb-isopropyl	0.9969	0.1-25	5	95.2	15.3	16.1	50	107.7	4.0	3.7	3.0
28	Benzobicyclon	0.9938	0.1-25	10	96.9	7.6	7.8	50	95.6	11.2	11.8	11.9
29	Benzoylprop-ethyl	0.9994	0.1-25	2.5	94.4	10.2	10.8	50	103.5	6.7	6.5	-12.6
30	Bistrifluron	0.9994	0.1-25	2.5	73.0	8.2	11.3	50	82.0	4.4	5.3	-20.1
31	Bromacil	0.9997	0.1-25	2.5	117.3	6.8	5.8	50	100.8	3.2	3.1	-7.8
32	Bromobutide	0.9955	0.1-25	10	111.6	6.7	6.0	50	92.9	9.3	10.0	-7.1
33	Buprofezin	0.9985	0.1-25	2.5	72.4	12.7	17.5	50	80.3	3.4	4.2	-14.5
34	Cadusafos	0.9978	0.1-25	5	81.9	10.1	12.3	50	93.9	3.0	3.2	-16.7

35	Carbaryl	0.9990	0.1-25	1	99.0	17.3	17.5	50	91.7	4.9	5.3	-7.3
36	Carbetamide	0.9995	0.1-25	1	88.9	15.6	17.6	50	99.1	6.1	6.1	-7.1
37	Carbofuran	0.9999	0.1-25	2.5	104.4	1.1	1.1	50	105.4	4.2	3.9	-2.5
38	Carbofuran-3-hydroxy	0.9977	0.1-25	10	73.5	1.8	2.4	50	81.0	2.2	2.7	-4.3
39	Carboxin	0.9991	0.1-25	2.5	89.8	11.4	12.7	50	90.4	9.8	10.8	-2.6
40	Carfentrazone-ethyl	0.9980	0.1-25	10	96.4	18.8	19.5	50	104.0	16.8	16.2	-8.7
41	Carpropamid	0.9994	0.1-25	2.5	91.1	14.0	15.3	50	87.4	5.8	6.6	-6.1
42	Chlorfenvinphos	0.9928	0.1-25	5	83.3	6.9	8.3	50	96.5	13.2	13.7	-3.1
43	Chlorfluazuron	0.9996	0.1-25	2.5	113.9	14.9	13.0	50	82.2	5.5	6.7	-35.3
44	Chloridazon	0.9998	0.1-25	1	104.3	10.8	10.4	50	90.1	4.1	4.6	-8.9
45	Chlorotoluron	0.9995	0.1-25	5	81.1	12.2	15.1	50	92.5	6.9	7.5	1.0
46	Chloroxuron	0.9913	0.1-25	2.5	117.7	16.7	14.2	50	84.1	5.5	6.6	16.4
47	Chlorpyrifos	0.9985	0.1-25	5	97.4	7.0	7.2	50	78.2	0.7	0.9	-18.7
48	Chlorsulfuron	0.9928	0.1-25	10	79.6	10.6	13.3	50	97.3	9.6	9.9	-4.0
49	Chromafenozide	0.9971	0.1-25	5	111.6	7.1	6.4	50	92.3	2.9	3.2	4.8
50	Clethodim	0.9969	0.1-25	5	89.5	13.1	14.7	50	93.1	4.9	5.2	-16.6
51	Clofentezine	0.9947	0.1-25	10	81.9	5.9	7.2	50	86.1	5.4	6.2	-16.4
52	Clothianidin	0.9999	0.1-25	2.5	89.4	12.5	14.0	50	88.0	3.1	3.5	1.1
53	Coumaphos	0.9997	0.1-25	2.5	106.4	1.8	1.7	50	94.0	4.4	4.7	-6.7

54	Crotoxyphos	0.9962	0.25-50	5	118.6	18.2	15.4	50	106.3	3.6	3.4	-13.8
55	Crufomate	0.9994	0.1-25	1	106.2	19.6	18.5	50	106.0	5.3	5.0	-5.9
56	Cyanazine	0.9996	0.1-25	2.5	109.8	11.0	10.0	50	89.5	3.0	3.3	-2.1
57	Cyantraniliprole	0.9983	0.1-25	10	103.5	9.6	9.3	50	103.2	8.2	8.0	-20.3
58	Cyazofamid	0.9994	0.1-25	5	98.1	14.3	14.6	50	94.9	2.8	3.0	-34.5
59	Cycloate	0.9972	0.1-25	10	83.1	7.8	9.4	50	79.5	9.4	11.8	-12.0
60	Cyclosulfamuron	0.9982	0.1-25	2.5	90.4	3.2	3.5	50	105.0	8.0	7.7	-3.0
61	Cyenopyrafen	0.9993	0.1-25	2.5	118.3	5.1	4.3	50	89.7	3.2	3.6	-38.1
62	Cyflufenamid	0.9992	0.1-25	2.5	86.3	8.4	9.7	50	103.2	8.3	8.1	-22.9
63	Cyflumetofen	0.9959	0.1-25	2.5	105.0	20.8	19.8	50	94.3	8.5	9.0	-2.4
64	Cymoxanil	0.9988	0.1-25	2.5	81.2	14.3	17.7	50	85.0	4.5	5.3	-14.8
65	Daimuron	0.9995	0.1-25	5	100.2	2.1	2.1	50	85.7	3.6	4.2	19.1
66	Demeton-O	0.9987	0.1-25	5	95.6	2.9	3.0	50	86.7	3.9	4.5	17.2
67	Demeton-S	0.9997	0.1-25	2.5	103.8	20.5	19.8	50	83.3	6.8	8.2	0.4
68	Demeton-S-methyl	0.9966	0.1-25	5	91.0	9.2	10.1	50	95.1	13.3	14.0	17.7
69	Demeton-S-methyl sulfoxide	0.9999	0.1-25	1	93.8	0.2	0.2	50	78.2	2.8	3.6	-2.2
70	Demeton-S-methyl-sulfone	0.9994	0.1-25	2.5	101.5	1.3	1.3	50	88.7	2.8	3.1	-1.5
71	Demeton-S-sulfone	1.0000	0.1-25	1	110.5	12.9	11.7	50	96.4	3.0	3.2	-3.9
72	Demeton-S-sulfoxide	1.0000	0.1-25	2.5	100.6	8.8	8.7	50	93.8	2.4	2.6	3.6

73	Desmetryn	0.9989	0.1-25	2.5	107.2	10.2	9.5	50	95.9	2.3	2.4	5.5
74	Diazinon	0.9995	0.1-25	5	97.3	17.5	18.0	50	82.7	9.8	11.9	-6.4
75	Dicrotophos	0.9998	0.1-25	2.5	102.9	1.6	1.6	50	88.8	0.3	0.3	-3.4
76	Diethatyl-ethyl	0.9984	0.1-25	5	98.6	7.7	7.8	50	98.3	7.3	7.4	0.5
77	Diethofencarb	0.9775	0.25-50	10	100.2	15.5	15.5	50	118.6	0.9	0.8	0.2
78	Diflufenican	0.9997	0.1-25	2.5	74.6	1.8	2.4	50	98.6	9.9	10.0	-7.9
79	Dimethachlor	0.9928	0.1-25	10	106.9	9.6	9.0	50	95.9	3.0	3.1	-16.1
80	Dimethametryn	0.9986	0.1-25	2.5	87.9	10.0	11.3	50	102.6	4.8	4.7	-10.1
81	Dimethenamide	0.9974	0.1-25	2.5	70.6	13.3	18.8	50	104.8	6.1	5.9	0.6
82	Dimethoate	0.9998	0.1-25	1	86.6	4.4	5.0	50	94.6	1.1	1.2	-3.5
83	Dimethylaminosulfotoluidide (DMST)	0.9981	0.1-25	10	100.9	11.3	11.2	50	80.2	4.8	6.0	2.6
84	Dimethylvinphos (Z)	0.9982	0.1-25	10	101.0	2.4	2.4	50	99.3	3.1	3.2	-6.2
85	Diniconazole	0.9957	0.1-25	2.5	76.5	12.7	16.6	50	102.1	11.6	11.4	-14.0
86	Diphenamid	0.9975	0.1-25	2.5	116.1	4.8	4.2	50	97.2	10.7	11.0	4.4
87	Disulfoton-sulfone	0.9991	0.1-25	1	119.4	12.4	10.4	50	105.1	3.6	3.4	-19.0
88	Disulfoton-sulfoxide	0.9981	0.1-25	5	92.9	11.1	11.9	50	90.9	2.6	2.9	14.4
89	Dithiopyr	0.9995	0.1-25	5	97.4	14.8	15.2	50	88.9	6.5	7.3	-8.9
90	Diuron	0.9994	0.1-25	1	94.2	2.8	3.0	50	97.0	7.6	7.8	-3.5

91	Edifenphos	0.9981	0.1-25	10	96.3	9.3	9.7	50	99.7	4.9	5.0	-8.7
92	Emamectin B1a	0.9927	0.1-25	5	99.8	7.3	7.3	50	89.5	9.6	10.8	20.8
93	Epoxiconazole	0.9980	0.1-25	10	107.0	14.3	13.4	50	102.2	11.9	11.6	-9.1
94	Esprocarb	0.9991	0.1-25	2.5	98.2	6.0	6.2	50	83.2	2.9	3.4	-7.2
95	Etaconazole	0.9947	0.1-25	10	91.6	12.9	14.1	50	99.4	9.7	9.8	-7.5
96	Ethaboxam	0.9978	0.1-25	2.5	108.0	16.6	15.3	50	105.4	7.4	7.1	-10.2
97	Ethiofencarb	0.9937	0.1-25	1	117.2	22.6	19.3	50	89.9	11.1	12.3	-13.7
98	Ethirimol	0.9997	0.1-25	2.5	102.6	2.6	2.5	50	84.4	0.9	1.1	-0.7
99	Ethoprophos	0.9993	0.1-25	2.5	84.7	15.0	17.7	50	96.1	2.0	2.0	-3.8
100	Ethoxysulfuron	0.9963	0.1-25	10	88.5	3.4	3.8	50	101.1	7.6	7.5	-0.1
101	Etoxazole	0.9997	0.1-25	2.5	98.8	5.6	5.7	50	83.6	2.6	3.2	-22.9
102	Etrimfos	0.9987	0.1-25	10	85.7	2.2	2.5	50	89.6	0.4	0.4	-2.0
103	Fenamiphos	0.9970	0.1-25	2.5	83.4	9.1	10.9	50	100.5	5.3	5.3	-12.1
104	Fenamiphos sulfone	0.9951	2.5-50	10	107.0	15.8	14.8	50	104.1	16.7	16.1	17.1
105	Fenamiphos sulfoxide	0.9992	0.1-25	1	119.7	22.3	18.7	50	100.8	9.6	9.5	5.6
106	Fenamiphos-sulfone	0.9985	0.1-25	10	111.2	12.0	10.8	50	113.4	4.4	3.9	-13.6
107	Fenamiphos-sulfoxide	0.9995	0.1-25	2.5	115.3	8.5	7.3	50	98.9	3.2	3.3	6.2
108	Fenazaquin	0.9995	0.1-25	5	72.9	3.5	4.8	50	85.6	5.8	6.8	-28.5
109	Fenfuram	0.9991	0.1-25	1	90.3	10.9	12.1	50	97.1	2.1	2.1	-3.1

110	Fenobucarb	0.9976	0.1-25	10	76.2	2.1	2.8	50	95.2	3.9	4.1	4.3
111	Fenoxanil	0.9987	0.1-25	2.5	97.3	10.0	10.3	50	104.9	8.5	8.1	-2.8
112	Fenoxaprop-ethyl	0.9999	0.1-25	2.5	89.7	3.6	4.0	50	93.0	6.0	6.4	-14.8
113	Fenoxycarb	0.9978	0.1-25	2.5	75.6	3.9	5.2	50	93.8	3.3	3.5	-8.2
114	Fenpropimorph	0.9988	0.1-25	2.5	99.6	14.6	14.6	50	80.7	7.6	9.4	-2.5
115	Fenpyroximate	0.9998	0.1-25	2.5	107.9	2.7	2.5	50	87.3	6.5	7.4	-17.5
116	Fenquinotrione_KIH-3635-M-2	0.9978	0.1-25	2.5	114.6	9.3	8.1	50	74.4	4.7	6.3	8.5
117	Fensulfothion	0.9973	0.1-25	2.5	115.4	9.6	8.3	50	107.0	3.7	3.4	5.1
118	Fenthion oxon	0.9940	0.1-25	5	78.9	14.5	18.3	50	101.6	4.3	4.2	-1.5
119	Fenthion oxon sulfone	0.9995	0.1-25	2.5	105.0	5.1	4.9	50	96.0	2.5	2.6	-1.6
120	Fenthion oxon sulfoxide	0.9998	0.1-25	1	104.1	5.0	4.8	50	91.6	1.6	1.8	-5.7
121	Fenthion sulfoxide	0.9969	0.1-25	5	96.5	2.3	2.3	50	94.7	3.0	3.2	-1.5
122	Fenthion-sulfone	0.9995	0.1-25	10	111.8	5.6	5.0	50	98.1	3.1	3.2	-5.8
123	Ferimzone	0.9966	1-25	10	96.5	11.5	11.9	50	96.2	3.3	3.4	35.9
124	Fipronil	0.9953	0.1-25	5	90.6	4.7	5.2	50	93.4	3.3	3.6	-8.0
125	Fipronil-sulfone	0.9999	0.1-25	2.5	115.3	21.2	18.4	50	94.7	5.6	5.9	-1.0
126	Flamprop-isopropyl	0.9961	0.1-25	5	92.6	4.3	4.6	50	119.3	18.9	15.9	-1.6
127	Flonicamid	0.9995	0.1-25	1	106.1	14.1	13.3	50	97.5	3.2	3.3	-11.4
128	Fluazinam	0.9858	0.25-50	2.5	87.9	10.5	12.0	50	78.5	0.6	0.7	-3.7

129	Flucetosulfuron	0.9967	0.1-25	5	112.3	13.6	12.1	50	96.1	8.9	9.2	51.7
130	Flufenacet	0.9982	0.1-25	2.5	82.0	6.2	7.6	50	97.3	1.3	1.4	-8.9
131	Flufenoxuron	0.9981	0.1-25	5	102.7	7.9	7.7	50	77.1	5.2	6.7	-0.8
132	Fluopicolide	0.9988	0.1-25	1	109.3	15.5	14.2	50	113.8	19.5	17.2	-2.4
133	Fluopyram	0.9975	0.1-25	2.5	97.4	9.5	9.8	50	96.7	1.9	1.9	16.6
134	Flupyradifurone	0.9996	0.1-25	1	96.3	5.7	6.0	50	96.6	3.3	3.4	-12.9
135	Fluridone	0.9989	0.1-25	2.5	115.7	3.4	3.0	50	103.7	5.5	5.3	-6.6
136	Flurtamone	0.9997	0.1-25	5	82.1	7.5	9.1	50	91.3	6.5	7.1	-1.5
137	Flusilazole	0.9937	0.1-25	5	117.7	13.6	11.6	50	75.4	9.3	12.3	27.3
138	Flutianil	0.9955	0.1-25	10	90.0	6.2	6.9	50	96.8	3.9	4.0	-14.8
139	Flutolanil	0.9979	0.1-25	5	96.1	14.1	14.7	50	90.2	13.0	14.4	4.1
140	Flutriafol	0.9975	0.1-25	5	93.1	4.8	5.1	50	88.4	4.4	5.0	17.1
141	Fluxametamide	0.9979	0.1-25	5	71.0	8.3	11.7	50	82.4	10.7	13.0	-12.8
142	Fluxapyroxad	0.9999	0.1-25	5	103.0	12.2	11.8	50	105.1	9.0	8.5	-7.3
143	Forchlorfenuron	0.9952	0.1-25	5	98.5	16.1	16.3	50	84.9	8.9	10.5	16.3
144	Fosthiazate	0.9981	0.1-25	2.5	95.7	3.3	3.5	50	103.0	11.4	11.1	-5.1
145	Furathiocarb	0.9999	0.1-25	10	82.1	10.7	13.0	50	76.8	3.4	4.5	-15.3
146	Heptenophos	0.9996	0.1-25	1	109.5	15.9	14.5	50	92.7	2.8	3.0	0.0
147	Hexaflumuron	0.9999	0.1-25	10	95.2	3.3	3.4	50	103.7	8.8	8.5	-15.1

148	Hexazinone	0.9998	0.1-25	1	92.5	14.3	15.4	50	94.1	10.8	11.5	-5.0
149	Hexythiazox	0.9999	0.1-25	2.5	80.8	3.1	3.8	50	79.0	3.3	4.2	-20.8
150	Imazalil	0.9959	0.1-25	5	115.8	17.0	14.7	50	87.1	4.6	5.3	23.7
151	Imazaquin	0.9968	0.1-25	2.5	102.1	15.6	15.3	50	72.0	4.7	6.5	12.2
152	Imazethapyr	0.9999	0.1-25	2.5	70.8	8.9	12.6	50	71.3	1.9	2.6	1.3
153	Imazosulfuron	0.9981	0.1-25	10	75.8	3.5	4.7	50	92.9	9.1	9.8	32.9
154	Imicyafos	0.9990	0.1-25	2.5	83.4	3.7	4.5	50	103.6	5.1	4.9	-11.3
155	Imidacloprid	1.0000	0.1-25	2.5	87.2	9.8	11.3	50	93.2	1.5	1.6	13.5
156	Indanofan	0.9974	0.1-25	5	99.4	9.1	9.2	50	83.4	5.6	6.8	21.2
157	Indoxacarb	0.9968	0.1-25	10	106.0	8.7	8.2	50	101.4	10.5	10.4	22.6
158	Ipfencarbazone	0.9755	0.25-50	10	96.5	8.0	8.3	50	98.1	2.5	2.5	-2.6
159	Iprobenfos	0.9990	0.5-25	5	104.0	15.3	14.7	50	95.1	16.8	17.7	-7.0
160	Iprovalicarb	0.9991	0.1-25	5	81.6	7.0	8.5	50	100.8	5.7	5.6	-6.5
161	Isoprocarb	0.9967	0.25-25	10	95.7	13.6	14.2	50	99.0	7.6	7.7	-15.0
162	Isoprothiolane	0.9942	0.25-25	10	104.8	2.6	2.5	50	94.8	1.3	1.3	-4.6
163	Isoproturon	0.9988	0.1-25	5	84.2	11.3	13.4	50	89.0	12.9	14.5	-8.6
164	Isopyrazam	0.9961	0.1-25	2.5	74.0	13.4	18.1	50	96.1	5.3	5.5	-24.8
165	Isoxaben	0.9966	0.1-25	2.5	70.5	8.7	12.4	50	110.9	4.5	4.0	7.0
166	Isoxathion	0.9997	0.1-25	1	116.5	19.2	16.5	50	90.8	5.8	6.4	-18.3

167	Lenacil	0.9992	1-25	5	98.6	5.1	5.2	50	92.5	5.3	5.7	-0.4
168	Linuron	0.9911	0.1-25	10	87.3	6.7	7.7	50	89.0	2.0	2.2	-5.9
169	Lufenuron	0.9985	0.1-25	2.5	109.5	13.0	11.8	50	88.8	16.6	18.7	3.6
170	Lufenuron	0.9923	0.1-25	10	84.8	16.8	19.8	50	99.6	6.5	6.5	4.2
171	Malaoxon	0.9976	0.1-25	1	111.2	10.1	9.1	50	101.4	1.0	1.0	7.9
172	Mandestrobin	0.9981	0.1-25	1	103.7	13.8	13.3	50	88.5	12.2	13.8	-9.8
173	Mandipropamid	0.9994	0.1-25	2.5	85.3	10.1	11.9	50	107.0	5.9	5.6	-4.6
174	Mefenacet	0.9958	0.1-25	1	71.9	11.2	15.5	50	87.9	2.2	2.5	3.8
175	Mefentrifluconazole	0.9944	0.1-25	10	99.8	18.2	18.2	50	87.4	10.5	12.0	4.1
176	Mepanipyrim	0.9988	0.1-25	10	105.5	19.7	18.6	50	90.7	9.9	10.9	2.2
177	Mephosfolan	0.9995	0.1-25	1	87.0	14.2	16.3	50	104.6	1.2	1.1	-0.7
178	Mepronil	0.9997	0.1-25	1	96.7	6.8	7.1	50	102.4	6.2	6.0	13.8
179	Metalaxyl	0.9939	0.1-25	5	115.5	5.4	4.7	50	94.7	6.3	6.7	7.9
180	Metamitron	0.9993	0.1-25	5	70.8	3.6	5.1	50	89.7	1.6	1.8	-6.6
181	Metazosulfuron	0.9960	0.1-25	10	113.2	20.0	17.7	50	115.2	21.9	19.0	3.2
182	Metconazole	0.9994	0.1-25	10	91.0	11.7	12.8	50	99.4	10.3	10.4	-0.1
183	Methabenzthiazuron	0.9926	0.1-25	2.5	112.1	12.8	11.4	50	92.7	3.1	3.3	-3.2
184	Methiocarb-sulfone	0.9999	0.1-25	1	118.8	13.3	11.2	50	89.7	4.4	4.9	-6.0
185	Methiocarb-sulfoxide	0.9997	0.1-25	2.5	80.8	3.5	4.4	50	95.7	2.9	3.0	-9.4

186	Methoprotryne	0.9999	0.1-25	1	80.8	9.8	12.2	50	112.2	9.0	8.0	-16.3
187	Methoxyfenozide	0.9993	0.1-25	2.5	107.3	19.7	18.4	50	92.6	15.9	17.1	3.3
188	Metobromuron	0.9997	0.1-25	1	97.1	11.8	12.2	50	97.4	3.9	4.0	-18.5
189	Metolcarb	0.9998	0.1-25	2.5	87.7	8.3	9.5	50	83.0	1.0	1.2	-6.9
190	Metominostrboin (Z)	0.9968	0.1-25	5	78.5	6.5	8.2	50	93.7	5.6	6.0	-0.8
191	Metominostrobin (E)	0.9971	0.1-25	2.5	80.9	3.2	4.0	50	103.9	0.7	0.7	-13.6
192	Metrafenon	0.9994	0.1-25	5	94.6	2.0	2.1	50	95.0	2.7	2.8	-11.0
193	Metrafenone	0.9995	0.1-25	2.5	81.6	11.5	14.1	50	95.7	7.0	7.3	-12.6
194	Mevinphos	0.9998	0.5-25	2.5	73.8	8.1	11.0	50	81.8	3.7	4.5	-6.2
195	Molinate	0.9943	0.1-25	5	117.0	6.8	5.8	50	88.6	6.7	7.5	5.9
196	Monocrotophos	0.9999	0.1-25	1	90.5	10.7	11.9	50	72.0	1.7	2.3	-8.1
197	Monolinuron	0.9985	0.1-25	2.5	85.1	6.6	7.7	50	95.2	5.0	5.2	2.5
198	Napropamide	0.9993	0.1-25	1	85.1	5.3	6.2	50	89.2	4.7	5.3	1.1
199	Neburon	0.9989	0.1-25	5	98.1	11.4	11.6	50	95.8	9.7	10.1	-2.7
200	Nicosulfuron	0.9989	0.1-25	10	95.7	5.8	6.0	50	88.4	7.0	8.0	-4.1
201	Nitenpyram	0.9994	0.1-25	1	119.7	6.4	5.3	50	71.8	2.4	3.4	1.8
202	Norflurazon	0.9931	0.1-25	5	82.1	3.4	4.1	50	94.5	5.4	5.8	6.2
203	Noruron (Norea)	0.9983	0.1-25	5	109.6	11.9	10.8	50	96.7	6.5	6.7	-8.2
204	Ofurace	0.9986	0.1-25	2.5	101.1	2.3	2.3	50	87.7	9.0	10.3	-1.4

205	Orysastrobin	0.9986	0.1-25	2.5	117.8	9.4	8.0	50	90.9	15.0	16.5	39.5
206	Oxadiazon	0.9954	0.1-25	10	85.8	13.6	15.8	50	86.3	1.5	1.7	-22.7
207	Oxadixyl	0.9999	0.1-25	1	103.9	2.3	2.2	50	89.2	1.3	1.4	-8.0
208	Oxamyl	0.9995	0.1-25	2.5	101.3	1.6	1.6	50	87.6	3.0	3.4	-4.8
209	Oxaziclomefone	0.9984	0.1-25	2.5	108.9	19.5	17.9	50	82.6	5.3	6.4	-14.9
210	Oxycarboxin	1.0000	0.1-25	1	116.8	11.3	9.7	50	93.4	7.0	7.5	2.2
211	Oxydemeton-methyl	0.9999	0.1-25	1	94.6	3.0	3.1	50	80.2	2.6	3.3	-1.0
212	Paraoxon-methyl	0.9995	0.1-25	1	118.2	20.8	17.6	50	86.9	6.1	7.0	-5.2
213	Pebulate	0.9927	0.25-50	10	107.9	19.4	18.0	50	96.7	18.6	19.2	-10.6
214	Penconazole	0.9989	0.1-25	5	97.6	19.2	19.7	50	98.2	12.9	13.1	-4.5
215	Pencycuron	0.9992	0.1-25	2.5	87.6	5.4	6.2	50	86.8	4.5	5.2	-7.8
216	Penoxsulam	0.9915	0.1-25	5	89.6	7.2	8.0	50	112.9	12.2	10.8	-16.0
217	Phenothrin_cis	0.9963	0.1-25	5	74.8	7.6	10.2	50	77.8	3.1	4.0	-29.3
218	Phenthoate	0.9961	0.1-25	2.5	102.3	20.1	19.6	50	91.3	8.8	9.6	-7.8
219	Phorate	0.9993	0.1-25	2.5	82.7	9.9	12.0	50	92.3	3.3	3.6	-3.9
220	Phorate-oxon	0.9935	0.1-25	5	112.9	8.4	7.4	50	88.5	4.9	5.6	34.0
221	Phorate-oxon-sulfone	0.9999	0.1-25	2.5	87.2	6.4	7.3	50	85.7	3.8	4.5	-3.5
222	Phorate-oxon-sulfoxide	0.9999	0.1-25	1	113.3	21.3	18.8	50	93.0	5.4	5.8	1.3
223	Phorate-sulfone	0.9994	1-25	5	95.8	17.1	17.9	50	91.1	7.2	7.9	-15.6

224	Phorate-sulfoxide	0.9992	0.1-25	5	89.6	14.7	16.4	50	91.9	3.1	3.4	2.1
225	Phosalone	0.9993	0.1-25	10	92.5	5.4	5.8	50	82.3	8.4	10.2	2.5
226	Phosfolan	0.9999	0.1-25	1	100.1	3.7	3.7	50	94.6	1.5	1.6	-3.6
227	Phosmet-oxon	0.9999	0.1-25	1	110.6	4.9	4.4	50	95.0	2.8	3.0	-2.2
228	Phosphamidon	0.9993	0.1-25	5	109.4	4.9	4.5	50	97.2	2.1	2.2	-2.1
229	Phoxim	0.9992	1-50	10	94.0	18.4	19.6	50	81.5	0.7	0.9	-22.2
230	Picarbutrazox	0.9980	0.1-25	2.5	71.7	7.9	11.1	50	101.7	12.4	12.2	-19.3
231	Picolinafen	0.9994	0.1-25	5	78.5	9.4	12.0	50	87.3	4.0	4.5	-12.1
232	Picoxystrobin	0.9992	0.1-25	2.5	98.9	10.1	10.2	50	93.1	16.8	18.0	-9.7
233	Pinoxaden	0.9996	0.1-25	2.5	94.9	7.5	7.9	50	74.8	5.8	7.7	-3.1
234	Piperonyl butoxide	0.9999	0.1-25	2.5	109.7	9.6	8.8	50	88.3	1.3	1.5	-18.6
235	Piperophos	0.9977	0.1-25	5	100.5	15.3	15.2	50	96.5	3.8	3.9	-7.2
236	Pirimicarb	0.9996	0.1-25	1	91.3	15.0	16.4	50	100.8	2.5	2.5	-2.9
237	Pirimicarb-desmethyl	0.9999	0.1-25	1	118.2	5.3	4.5	50	92.7	0.1	0.2	-3.4
238	Pirimiphos-ethyl	0.9999	0.1-25	2.5	84.3	16.6	19.8	50	83.1	2.2	2.6	-14.4
239	Pirimiphos-methyl	0.9998	0.1-25	2.5	73.8	4.5	6.1	50	85.1	7.5	8.8	-8.4
240	Probenazole	0.9990	0.5-25	5	100.4	15.3	15.3	50	93.7	6.2	6.6	-5.7
241	Prochloraz	0.9949	0.1-25	5	103.3	19.1	18.5	50	94.5	9.2	9.7	-13.3
242	Profenofos	0.9980	0.1-25	2.5	93.4	10.8	11.6	50	84.5	4.8	5.7	-17.4

243	Promecarb	0.9991	0.1-25	10	72.2	13.3	18.4	50	77.9	6.5	8.3	6.4
244	Prometryn	0.9989	0.1-25	2.5	110.6	9.8	8.8	50	87.6	2.3	2.6	8.8
245	Pronamide (Propyzamide)	0.9984	0.1-25	5	87.2	10.9	12.5	50	91.6	3.8	4.2	15.1
246	Propachlor	0.9955	0.1-25	10	100.1	15.6	15.5	50	92.8	13.9	15.0	2.4
247	Propanil	0.9965	0.5-25	10	81.4	14.6	18.0	50	100.1	12.9	12.9	12.0
248	Propaquizafop	0.9994	0.1-25	2.5	79.2	9.3	11.7	50	92.5	5.1	5.5	-10.8
249	Propargite	0.9997	0.1-25	2.5	97.1	1.7	1.7	50	87.0	1.6	1.8	-16.1
250	Propazine	0.9946	0.1-25	2.5	110.4	16.4	14.9	50	93.5	1.8	2.0	-4.3
251	Propiconazole	0.9854	0.25-50	10	117.7	8.5	7.2	50	107.6	10.4	9.7	-6.2
252	Propoxur	0.9997	0.1-25	1	94.1	12.3	13.1	50	92.6	5.3	5.7	0.3
253	Proquinazid	0.9994	0.1-25	2.5	93.0	0.9	1.0	50	74.3	0.3	0.4	-39.8
254	Prosulfocarb	0.9991	0.5-25	2.5	105.1	18.7	17.8	50	83.4	1.8	2.1	-7.8
255	Prothioconazole-desthio	0.9984	0.1-25	2.5	103.2	17.9	17.3	50	90.7	1.9	2.1	-8.6
256	Pydiflumetofen	0.9997	0.1-25	5	95.4	2.8	3.0	50	95.0	6.0	6.3	-14.6
257	Pyflubumide	0.9995	0.1-25	2.5	108.5	16.2	14.9	50	90.9	3.2	3.5	-21.9
258	Pyflubumide-NH	0.9939	0.1-25	10	93.2	16.7	18.0	50	93.4	9.2	9.8	-13.8
259	Pyracarbolid	0.9991	0.1-25	1	80.3	13.1	16.3	50	92.3	2.7	2.9	-12.0
260	Pyraclofos	0.9972	0.1-25	5	89.5	13.5	15.0	50	98.2	9.2	9.4	-5.6
261	Pyraclonil	0.9998	0.1-25	5	89.3	11.0	12.3	50	104.9	10.6	10.1	-4.3

262	Pyraclostrobin	0.9991	0.1-25	10	97.6	11.4	11.7	50	98.7	9.9	10.0	-16.7
263	Pyraflufen-ethyl	0.9990	0.1-25	5	75.1	13.6	18.2	50	85.5	15.7	18.3	2.7
264	Pyraziflumid	0.9990	0.1-25	10	99.1	9.7	9.8	50	109.6	1.6	1.5	0.8
265	Pyrazolate	0.9999	0.1-25	2.5	73.9	12.0	16.3	50	88.6	14.8	16.7	-2.8
266	Pyrazophos	0.9841	0.25-50	10	110.5	9.1	8.3	50	99.0	12.0	12.1	-4.1
267	Pyrazoxyfen	0.9987	0.5-25	5	84.0	14.2	16.9	50	93.9	15.7	16.7	4.1
268	Pyribencarb	0.9980	0.1-25	5	101.7	2.9	2.8	50	101.5	7.5	7.4	-6.4
269	Pyributicarb	0.9976	0.1-25	5	104.0	3.0	2.9	50	82.1	6.0	7.3	-15.5
270	Pyridaben	0.9977	0.1-25	5	101.8	6.0	5.9	50	80.6	4.2	5.3	-37.1
271	Pyridaphenthion	0.9990	0.1-25	2.5	74.0	11.6	15.6	50	98.7	6.9	7.0	-4.0
272	Pyrifluquinazon	0.9983	0.1-25	2.5	119.4	13.7	11.5	50	83.9	9.4	11.2	22.2
273	Pyriftalid	0.9996	0.1-25	2.5	99.3	5.8	5.8	50	99.8	6.7	6.7	-9.9
274	Pyrimethanil	0.9969	0.5-50	5	70.9	9.0	12.7	50	79.7	0.7	0.9	4.4
275	Pyrimidifen	0.9995	0.1-25	2.5	98.3	7.1	7.2	50	86.6	2.0	2.4	-31.8
276	Pyrimisulfan	0.9930	0.1-25	5	72.6	7.8	10.8	50	96.3	15.4	15.9	-9.9
277	Pyriofenone	0.9977	0.1-25	5	103.3	7.7	7.4	50	90.7	7.1	7.9	-5.0
278	Pyriproxyfen	0.9974	0.1-25	2.5	107.7	18.5	17.2	50	79.4	3.9	4.9	-21.7
279	Pyroquilon	0.9990	0.1-25	2.5	79.2	12.9	16.3	50	93.7	6.9	7.3	-8.4
280	Quinoclamine	0.9959	1-50	5	98.7	16.5	16.7	50	91.7	5.8	6.4	-13.7

281	Quizalofop-ethyl	0.9979	0.1-25	5	109.9	10.7	9.7	50	91.9	8.7	9.5	-6.3
282	Secbumeton	0.9997	0.1-25	1	101.6	14.9	14.6	50	103.6	8.4	8.1	-3.6
283	Sedaxane_cis	0.9770	0.25-50	10	105.2	4.7	4.5	50	114.4	10.2	8.9	-20.2
284	Sedaxane_trans	0.9917	0.1-25	5	82.6	9.1	11.0	50	103.5	5.5	5.3	-18.8
285	Sethoxydim	0.9991	0.5-25	5	112.3	15.3	13.7	50	88.3	1.4	1.6	-8.9
286	Simetryn	0.9957	0.1-25	5	76.0	4.3	5.6	50	103.7	7.4	7.1	-18.8
287	Spinetoram (J)	0.9971	0.1-25	5	111.3	3.9	3.5	50	93.3	6.4	6.9	-11.4
288	Spinosyn A	0.9962	0.1-25	5	114.0	10.6	9.3	50	94.6	5.2	5.5	-15.7
289	Spinosyn D	0.9953	0.1-25	2.5	110.5	16.4	14.8	50	97.0	9.6	9.9	-19.3
290	Spirodiclofen	0.9987	0.1-25	5	97.0	8.4	8.7	50	80.0	3.6	4.5	-8.9
291	Spiroxamine	0.9969	0.1-25	5	95.7	7.6	7.9	50	71.4	4.3	6.1	15.5
292	Sulfotep	0.9959	0.1-25	2.5	112.2	11.3	10.1	50	93.2	11.3	12.1	-10.3
293	Sulprofos	0.9991	0.1-25	2.5	85.3	13.2	15.4	50	76.1	3.1	4.1	-17.7
294	Tau-fluvalinate	0.9949	0.25-25	10	91.1	3.7	4.1	50	81.2	9.0	11.1	9.6
295	Tebuconazole	0.9991	0.1-25	2.5	96.1	6.3	6.5	50	90.7	3.4	3.8	0.0
296	Tebufenpyrad	0.9992	0.5-25	10	80.7	6.2	7.7	50	93.9	1.4	1.5	-6.9
297	Tebufloquin	0.9999	0.1-25	5	90.2	7.3	8.1	50	93.7	4.4	4.7	-11.7
298	Tebuthiuron	0.9998	0.1-25	1	94.0	18.4	19.5	50	99.7	10.5	10.5	-15.5
299	Teflubenzuron	0.9994	0.1-25	1	96.0	15.2	15.8	50	89.7	7.3	8.2	-23.1

300	Terbufos-oxon	0.9957	0.25-25	10	96.5	4.4	4.6	50	93.9	4.4	4.7	-0.8
301	Terbufos-oxon-sulfone	0.9997	0.1-25	1	83.6	4.2	5.1	50	91.7	6.3	6.9	-1.9
302	Terbufos-oxon-sulfoxide	0.9999	0.1-25	1	90.1	5.1	5.6	50	97.3	1.3	1.3	-3.4
303	Terbufos-sulfone	0.9997	1-25	5	97.6	17.5	18.0	50	93.2	1.6	1.7	-3.7
304	Terbufos-sulfoxide	0.9988	0.1-25	2.5	70.6	10.5	14.9	50	87.7	5.7	6.5	5.3
305	Terbuthylazine	0.9998	0.1-25	2.5	104.3	7.4	7.1	50	99.1	10.5	10.6	6.1
306	Terbutryn	0.9995	0.1-25	1	79.7	13.1	16.4	50	98.3	8.3	8.5	-14.4
307	Tetraconazole	0.9977	0.1-25	5	96.0	9.0	9.4	50	108.0	8.2	7.6	-40.3
308	Thenylchlor	0.9770	0.25-50	2.5	88.0	9.3	10.5	50	95.1	5.5	5.8	1.7
309	Thiabendazole	0.9999	0.1-25	2.5	94.9	3.2	3.4	50	81.1	2.0	2.4	-5.1
310	Thiacloprid	0.9999	0.1-25	1	117.8	8.8	7.4	50	93.1	1.8	1.9	-8.2
311	Thiamethoxam	1.0000	0.1-25	1	102.9	11.5	11.2	50	89.4	2.0	2.2	-6.5
312	Thiazopyr	0.9974	0.1-25	2.5	119.5	5.6	4.7	50	87.6	1.1	1.2	7.4
313	Thidiazuron	0.9997	0.1-25	1	116.1	12.4	10.7	50	89.4	10.2	11.4	-12.9
314	Thidiazuron	0.9980	0.1-25	10	74.7	2.6	3.4	50	93.0	10.5	11.3	-4.8
315	Thifensulfuron-methyl	0.9957	2.5-50	10	104.5	9.4	9.0	50	87.4	5.4	6.2	16.5
316	Thiobencarb	0.9980	0.1-25	2.5	96.5	18.7	19.4	50	86.4	3.0	3.5	-7.9
317	Thionazin	0.9930	0.1-25	10	82.9	5.0	6.1	50	100.6	15.0	14.9	-10.0
318	Tolfenpyrad	0.9990	0.5-25	5	102.5	14.4	14.1	50	86.6	2.3	2.7	-6.7

319	Triadimefon	0.9944	0.1-25	10	91.0	3.1	3.4	50	91.0	7.7	8.5	-18.2
320	Triafamone	0.9993	0.1-25	2.5	107.0	7.8	7.3	50	104.3	10.7	10.2	0.6
321	Triazamate	0.9989	0.1-25	2.5	116.2	12.9	11.1	50	94.2	1.6	1.7	13.9
322	Tribufos	0.9991	0.1-25	2.5	105.6	2.3	2.2	50	82.0	0.9	1.1	-37.8
323	Trifloxystrobin	0.9970	0.1-25	1	114.0	15.3	13.5	50	96.8	3.6	3.7	-22.7
324	Triflumizole	0.9987	0.1-25	2.5	88.9	8.8	9.9	50	101.1	2.7	2.7	-28.1
325	Triflumuron	0.9981	0.1-25	10	78.6	5.8	7.4	50	88.5	4.0	4.5	1.8
326	TZ-1E	0.9940	0.1-25	10	104.3	7.3	7.0	50	114.2	3.1	2.7	-15.0
327	Valifenalate	0.9977	0.1-25	10	102.3	7.6	7.5	50	98.0	8.4	8.6	-9.1
328	Vamidothion	1.0000	0.1-25	1	98.7	12.4	12.6	50	93.8	0.9	0.9	-3.8
329	Vernolate	0.9969	0.1-25	2.5	89.5	2.1	2.4	50	81.6	6.7	8.3	1.3
330	XMC	0.9934	0.1-25	5	107.0	6.1	5.7	50	84.1	8.1	9.6	18.9
331	Zoxamide	0.9942	0.1-25	5	89.2	10.2	11.4	50	88.9	2.7	3.1	40.9

SD: Standard Deviation

RSD: Relative Standard Deviation

Table S5. Method validation data for pesticide residue analysis in black soybean

No.	Compound name	r^2	Linear range (µg/kg)	Sample preparation method 3 (type 3 d-SPE sorbent)								
				Low (LOQ) (µg/kg)	Recovery %	SD	RSD %	High (µg/kg)	Recovery %	SD	RSD %	Matrix effect %
1	2,3,5-trimethacarb	0.9992	0.1-50	10	81.2	10.4	12.8	50	79.7	8.5	10.7	-22.8
2	3,4,5-trimethacarb	0.9997	0.1-50	5	71.1	10.6	14.9	50	82.3	13.7	16.7	-26.1
3	3-hydroxycarbofuran	0.9996	0.1-50	5	70.8	6.1	8.6	50	80.4	7.8	9.7	-24.3
4	6-Benzyl aminopurine	0.9994	0.1-50	5	71.7	5.6	7.8	50	77.9	7.1	9.2	-39.9
5	Abamectin_B1a	0.9915	0.1-50	5	76.1	8.2	10.8	50	73.6	7.9	10.7	-65.8
6	Acetamiprid	0.9955	0.1-50	10	80.2	1.5	1.9	50	90.0	4.4	4.9	-32.8
7	Alachlor	0.9990	0.1-50	10	74.8	12.9	17.3	50	80.1	4.9	6.1	-19.4
8	Aldicarb	0.9983	0.1-50	5	82.9	6.2	7.5	50	87.6	8.2	9.3	-26.9
9	Alidcarb sulfone	0.9994	0.1-50	10	81.9	0.3	0.3	50	82.2	5.5	6.7	-23.2
10	Ametoctradin	0.9977	0.1-50	2.5	101.4	18.1	17.9	50	80.3	4.2	5.2	-26.1
11	Anilofos	0.9977	0.1-25	5	73.6	14.1	19.2	50	80.4	10.0	12.5	-2.8
12	Aramite	0.9971	1-50	10	109.0	2.6	2.4	50	70.3	3.8	5.5	-12.5
13	Atrazine	0.9945	0.1-50	10	73.7	13.4	18.2	50	79.5	6.6	8.3	-27.0
14	Azaconazole	0.9943	0.1-50	2.5	80.0	7.8	9.7	50	79.5	11.5	14.5	-13.9
15	Azamethiphos	0.9992	0.1-50	5	70.3	5.4	7.7	50	91.9	5.9	6.4	-26.2

16	Azimsulfuron	0.9982	0.1-25	2.5	86.9	8.0	9.2	50	96.8	19.3	20.0	14.4
17	Azinphos-methyl	0.9960	0.25-25	2.5	99.1	15.6	15.7	50	76.2	11.2	14.7	-15.4
18	Azoxystrobin	0.9996	0.1-50	2.5	80.0	12.5	15.6	50	83.6	7.9	9.5	-31.9
19	Benalaxyl	0.9994	0.1-50	1	117.3	20.3	17.3	50	81.4	0.4	0.4	-19.4
20	Bendiocarb	0.9966	0.1-50	10	79.9	10.0	12.5	50	86.4	7.8	9.1	-23.6
21	Benfuracarb	0.9960	2.5-50	10	118.9	7.4	6.2	50	70.3	4.0	5.6	-2.2
22	Benodanil	0.9978	0.1-50	10	70.6	3.2	4.6	50	76.4	11.6	15.2	-12.0
23	Benoxacor	0.9979	0.1-50	10	76.8	4.4	5.7	50	70.7	11.9	16.8	-26.4
24	Bensulfuron-methyl	0.9987	0.1-50	5	99.4	15.2	15.3	50	89.9	5.6	6.2	-13.4
25	Bensulide	0.9934	0.1-50	10	109.4	13.3	12.1	50	89.5	10.6	11.9	7.7
26	Benthiavalicarb-isopropyl	0.9987	0.1-50	10	84.2	16.0	19.0	50	79.9	9.3	11.7	-22.6
27	Benzobicyclon	0.9946	0.1-50	10	71.1	2.3	3.2	50	88.5	13.8	15.5	-12.9
28	Benzoximate	0.9993	0.1-50	2.5	71.7	9.3	13.0	50	77.4	4.1	5.3	-23.8
29	Benzoylprop-ethyl	0.9983	0.1-50	10	79.1	15.6	19.7	50	76.1	7.6	10.0	-18.4
30	Bistrifluron	0.9972	0.25-50	5	119.9	3.4	2.8	50	73.3	6.6	9.0	-5.9
31	Bixafen	0.9991	0.1-50	10	78.5	13.4	17.0	50	83.2	14.8	17.8	-3.7
32	Boscalid	0.9988	0.1-25	10	71.8	3.4	4.8	50	86.2	11.9	13.8	-6.1
33	Bromacil	0.9972	0.1-50	10	75.9	2.5	3.4	50	87.3	4.6	5.3	-32.7
34	Bromobutide	0.9956	0.1-25	10	72.9	2.4	3.3	50	76.3	4.9	6.4	6.9

35	Bupirimate	0.9996	0.1-50	5	84.7	13.5	15.9	50	80.6	9.3	11.5	-5.5
36	Cadusafos	0.9985	0.1-25	10	77.2	6.6	8.5	50	72.0	6.5	9.1	-4.7
37	Cafenstrole	0.9977	0.1-50	10	104.3	8.1	7.8	50	104.1	7.0	6.7	-8.8
38	Carbaryl	0.9992	0.1-50	10	76.5	13.8	18.1	50	78.8	6.9	8.7	-24.1
39	Carbendazim	0.9963	0.25-50	1	112.5	10.2	9.1	50	87.9	4.0	4.6	-16.1
40	Carbetamide	0.9995	0.1-50	5	74.6	3.9	5.2	50	86.7	4.8	5.6	-21.1
41	Carbofuran	0.9995	0.1-50	10	86.8	7.0	8.0	50	82.1	5.0	6.0	-23.4
42	Carbofuran-3-hydroxy	0.9996	0.1-50	10	77.0	4.4	5.8	50	85.8	5.7	6.6	-23.5
43	Carboxin	0.9985	0.1-50	10	77.4	5.0	6.4	50	85.5	3.0	3.5	-33.3
44	Carfentrazone-ethyl	0.9998	0.1-25	10	73.7	11.1	15.1	50	75.2	11.1	14.7	14.0
45	Chlorantraniliprole	0.9967	0.25-25	10	95.0	8.2	8.7	50	70.6	5.9	8.4	-7.8
46	Chlorbenzuron	0.9975	0.25-50	10	113.5	10.9	9.6	50	81.1	15.1	18.7	0.9
47	Chlorfenvinphos	0.9955	0.1-50	5	71.1	9.0	12.7	50	89.7	7.6	8.5	-23.5
48	Chloridazon	0.9977	0.1-50	10	78.9	4.8	6.0	50	85.5	4.2	4.9	-29.9
49	Chlorimuron-ethyl	0.9981	0.1-50	2.5	95.4	10.5	11.0	50	75.0	3.6	4.8	-19.9
50	Chlorotoluron	0.9989	0.1-50	2.5	74.2	2.9	3.9	50	89.0	4.3	4.9	-23.2
51	Chloroxuron	0.9949	0.1-50	10	85.4	13.0	15.2	50	91.4	8.4	9.1	-23.8
52	Chlorsulfuron	0.9948	0.1-50	5	111.0	13.4	12.1	50	71.6	8.7	12.2	19.4
53	Chromafenozide	0.9940	0.1-50	10	86.4	13.5	15.6	50	80.1	3.1	3.8	-7.9

54	Clomazone	0.9957	0.25-50	10	115.0	13.0	11.3	50	99.1	14.7	14.8	-29.5
55	Clothianidin	0.9945	0.1-50	10	76.9	7.8	10.1	50	87.7	5.0	5.7	-37.5
56	Coumaphos	0.9906	0.1-25	10	77.7	13.1	16.8	50	72.2	9.0	12.4	19.0
57	Crotoxyphos	0.9961	0.25-25	2.5	79.6	15.7	19.8	50	79.3	1.1	1.4	-12.5
58	Crufomate	0.9946	0.1-50	10	80.0	12.0	15.0	50	82.2	2.5	3.1	-15.2
59	Cyanazine	0.9981	0.1-50	10	79.4	10.9	13.8	50	87.3	5.0	5.7	-11.7
60	Cyantraniliprole	0.9997	0.1-50	5	78.1	12.0	15.3	50	83.7	10.8	12.9	-37.3
61	Cyazofamid	0.9981	0.1-50	10	89.8	11.7	13.0	50	83.0	1.2	1.5	-18.6
62	Cyclaniliprole	0.9959	0.25-50	10	101.7	16.0	15.7	50	91.2	12.5	13.7	-41.3
63	Cyclosulfamuron	0.9991	0.1-50	5	75.6	13.3	17.6	50	89.0	11.2	12.6	-23.0
64	Cyflufenamid	0.9997	0.1-50	5	84.5	6.8	8.1	50	73.9	3.7	5.0	-23.4
65	Cyflumetofen	0.9983	0.1-50	10	72.6	13.5	18.6	50	71.9	12.5	17.3	-10.6
66	Cymoxanil	0.9993	0.1-25	10	84.1	10.5	12.4	50	80.4	1.3	1.6	-7.1
67	Daimuron	0.9998	0.1-50	2.5	102.1	10.8	10.5	50	81.2	8.7	10.7	-18.9
68	Demeton-S	0.9967	0.1-25	2.5	78.4	13.5	17.2	50	88.0	6.6	7.5	-21.9
69	Demeton-S-methyl	0.9902	0.1-25	5	79.6	14.1	17.8	50	85.5	7.5	8.8	-0.7
70	Demeton-S-methyl sulfoxide	0.9999	0.1-50	10	74.2	0.2	0.3	50	73.7	3.5	4.8	-23.2
71	Demeton-S-methyl-sulfone	0.9999	0.1-50	2.5	73.2	3.2	4.4	50	83.0	4.7	5.7	-22.6
72	Demeton-S-sulfone	0.9962	0.1-50	10	79.6	5.3	6.7	50	92.8	4.2	4.6	-21.9

73	Demeton-S-sulfoxide	0.9996	0.1-50	5	76.8	4.1	5.3	50	83.3	3.7	4.5	-34.4
74	Desmetryn	0.9990	0.1-50	10	77.8	5.0	6.5	50	79.0	5.7	7.2	-12.3
75	Diazinon	0.9934	0.25-50	5	120.0	13.6	11.3	50	88.9	3.2	3.6	-19.9
76	Dichlorvos	0.9938	2.5-50	10	74.0	7.2	9.7	50	90.9	3.2	3.5	-27.2
77	Diclosulam	0.9957	0.25-25	5	99.7	19.3	19.3	50	80.7	8.2	10.1	42.0
78	Dicrotophos	0.9996	0.1-50	5	77.3	1.8	2.4	50	80.3	4.2	5.2	-25.0
79	Diethatyl-ethyl	0.9996	0.1-50	10	88.7	7.0	7.8	50	85.2	3.3	3.9	-18.0
80	Diethofencarb	0.9965	0.1-50	10	71.5	6.3	8.7	50	82.3	9.9	12.1	-29.0
81	Diflufenican	0.9954	0.25-50	2.5	89.0	1.5	1.6	50	70.4	8.0	11.4	-21.7
82	Dimethachlor	0.9987	0.1-50	10	76.9	3.9	5.0	50	83.8	6.2	7.4	-30.0
83	Dimethametryn	0.9968	0.25-50	5	113.7	7.3	6.4	50	92.1	15.4	16.7	-15.8
84	Dimethenamide	0.9993	0.1-50	10	78.8	4.3	5.5	50	74.5	14.6	19.6	-20.2
85	Dimethoate	0.9974	0.1-50	10	81.2	4.6	5.6	50	91.3	4.0	4.4	-26.5
86	Dimethylaminosulfotoluidide (DMST)	0.9978	0.1-50	5	72.2	13.2	18.2	50	91.9	3.9	4.2	-12.8
87	Diniconazole	0.9920	0.1-50	10	85.8	9.4	11.0	50	78.7	6.8	8.6	-33.4
88	Diphenamid	0.9992	0.1-50	5	74.1	11.0	14.8	50	90.2	5.3	5.9	-30.4
89	Disulfoton-sulfone	0.9985	0.1-25	5	78.8	13.1	16.7	50	86.7	3.7	4.3	-9.0
90	Disulfoton-sulfoxide	0.9951	0.1-50	5	73.6	2.4	3.3	50	86.0	7.3	8.5	-19.5

91	Diuron	0.9979	0.1-50	5	106.1	9.6	9.1	50	75.5	5.1	6.8	-3.5
92	Edifenphos	0.9980	0.1-25	10	72.1	4.7	6.5	50	74.4	4.3	5.7	2.1
93	Emamectin B1a	0.9997	0.1-50	5	83.2	12.1	14.5	50	79.4	5.3	6.7	-6.9
94	Epoxiconazole	0.9991	0.1-50	10	80.4	8.5	10.6	50	85.3	6.6	7.7	-22.1
95	Etaconazole	0.9947	0.1-50	10	77.7	3.6	4.6	50	81.1	7.7	9.4	-19.2
96	Ethaboxam	0.9984	0.1-50	5	71.0	1.6	2.3	50	81.9	5.2	6.3	1.8
97	Ethiofencarb	0.9933	0.1-25	10	75.5	1.5	2.0	50	77.6	5.1	6.6	-11.9
98	Ethirimol	0.9994	0.1-50	5	71.6	2.7	3.7	50	75.3	5.3	7.0	-28.7
99	Ethoprophos	0.9960	0.1-50	10	72.2	9.1	12.6	50	87.8	14.7	16.7	-21.9
100	Ethoxysulfuron	0.9994	0.1-50	10	77.7	8.0	10.3	50	79.9	4.5	5.7	-13.0
101	Etrimfos	0.9936	0.25-50	5	102.8	11.9	11.6	50	82.4	11.7	14.2	-14.4
102	Fenamiphos	0.9959	0.1-50	10	82.3	13.7	16.7	50	84.8	8.5	10.0	-14.8
103	Fenamiphos sulfoxide	0.9958	0.1-50	10	81.0	9.1	11.3	50	98.4	7.9	8.1	-10.2
104	Fenamiphos-sulfone	0.9985	0.1-50	10	75.5	2.2	3.0	50	85.3	4.0	4.7	-0.6
105	Fenamiphos-sulfoxide	0.9961	0.1-50	5	74.3	7.1	9.5	50	90.7	6.7	7.4	-20.1
106	Fenbuconazole	0.9987	0.5-25	2.5	96.4	16.1	16.7	50	81.8	13.4	16.3	18.5
107	Fenfuram	0.9999	0.1-50	1	82.3	6.4	7.8	50	80.3	5.1	6.3	-25.7
108	Fenobucarb	0.9989	0.1-50	5	89.8	15.7	17.4	50	78.7	10.3	13.1	-31.5
109	Fenoxanil	0.9928	0.25-50	5	116.5	9.4	8.1	50	105.5	1.9	1.8	1.1

110	Fenoxaprop-ethyl	0.9964	0.25-50	2.5	97.8	8.4	8.6	50	73.1	2.0	2.8	-18.3
111	Fensulfothion	0.9987	0.1-25	2.5	91.2	6.1	6.7	50	80.6	4.5	5.6	12.9
112	Fenthion oxon	0.9955	0.1-50	5	73.8	4.5	6.0	50	91.9	7.9	8.6	-32.7
113	Fenthion oxon sulfone	0.9994	0.1-50	5	72.2	8.1	11.2	50	88.5	2.8	3.2	-25.9
114	Fenthion oxon sulfoxide	0.9991	0.1-50	2.5	70.8	10.8	15.3	50	81.8	4.7	5.7	-24.3
115	Fenthion sulfoxide	1.0000	0.1-50	2.5	85.6	4.9	5.7	50	93.9	4.8	5.1	-19.8
116	Fenthion-sulfone	0.9960	0.1-50	5	81.0	4.0	4.9	50	91.2	7.1	7.7	-12.3
117	Fipronil-sulfone	0.9964	0.1-50	10	76.1	6.1	8.0	50	91.3	7.0	7.6	-17.2
118	Flamprop-isopropyl	0.9908	0.1-50	10	70.1	12.2	17.4	50	78.6	13.4	17.1	-11.6
119	Flonicamid	0.9998	0.1-50	2.5	70.2	9.9	14.1	50	87.0	5.5	6.3	-25.0
120	Fluazinam	0.9959	0.25-50	2.5	114.3	13.6	11.9	50	76.3	5.0	6.5	-17.2
121	Flucetosulfuron	0.9804	0.25-25	2.5	112.5	7.6	6.7	50	70.6	10.0	14.2	12.6
122	Flufenacet	0.9980	0.1-50	5	82.7	12.9	15.6	50	83.9	4.8	5.7	-20.2
123	Flufenoxuron	0.9939	0.25-50	10	98.5	9.3	9.4	50	79.9	6.7	8.4	17.3
124	Fluometuron	0.9957	0.1-50	10	92.1	18.1	19.7	50	91.6	2.0	2.2	-23.4
125	Fluopicolide	0.9978	0.1-25	5	71.7	9.8	13.7	50	78.1	12.5	16.0	2.1
126	Fluopyram	0.9917	0.25-50	10	119.1	12.3	10.3	50	109.7	0.7	0.7	-18.0
127	Flupyradifurone	0.9951	0.1-50	10	77.9	2.8	3.5	50	92.0	1.0	1.1	-13.2
128	Fluquinconazole	0.9932	0.1-25	10	76.0	5.6	7.3	50	70.6	4.2	6.0	2.2

129	Fluridone	0.9996	0.1-50	2.5	80.1	4.4	5.5	50	85.4	2.1	2.4	-30.4
130	Flurochloridone	0.9929	0.25-25	10	83.7	8.1	9.6	50	78.2	10.9	13.9	-10.5
131	Flurtamone	0.9991	0.1-50	10	80.7	7.1	8.8	50	86.2	6.9	8.0	-45.1
132	Flusilazole	0.9957	0.1-50	10	84.0	14.0	16.6	50	90.1	4.4	4.8	-19.6
133	Fluthiacet-methyl	0.9992	0.1-50	10	85.2	7.7	9.0	50	74.4	3.0	4.0	-6.7
134	Flutianil	0.9995	0.1-25	5	72.1	15.4	21.4	50	82.0	7.7	9.4	9.2
135	Flutolanil	0.9993	0.1-25	5	84.2	3.9	4.6	50	77.3	2.7	3.5	5.9
136	Flutriafol	0.9971	0.1-25	5	98.0	6.4	6.6	50	92.4	3.9	4.2	0.2
137	Fluxapyroxad	0.9951	0.1-50	10	73.4	1.7	2.4	50	99.9	14.7	14.7	-17.8
138	Forchlorfenuron	0.9997	0.1-50	5	90.4	9.3	10.3	50	81.2	13.3	16.4	-25.6
139	Fosthiazate	0.9974	0.1-50	5	81.0	10.7	13.2	50	89.1	8.9	10.0	-22.6
140	Halosulfuron-methyl	0.9988	0.1-50	5	79.0	12.0	15.2	50	82.8	4.6	5.5	-7.6
141	Heptenophos	0.9952	0.1-50	2.5	105.8	5.3	5.0	50	88.1	11.1	12.6	-19.5
142	Hexazinone	0.9912	0.1-50	10	75.5	8.8	11.6	50	90.6	2.3	2.5	-28.0
143	Imazalil	0.9996	0.1-25	2.5	96.7	3.0	3.1	50	74.0	14.0	19.0	8.6
144	Imazethapyr	0.9968	0.25-50	2.5	96.4	5.7	5.9	50	75.6	2.4	3.2	-10.7
145	Imazosulfuron	0.9943	0.1-50	10	108.3	4.5	4.1	50	88.6	14.9	16.8	45.5
146	Imicyafos	0.9966	0.1-50	10	80.8	4.0	4.9	50	85.4	2.6	3.0	-27.2
147	Imidacloprid	0.9969	0.1-50	10	72.7	1.0	1.4	50	86.0	3.2	3.7	-5.8

148	Inabenfide	0.9920	0.25-50	10	95.1	18.7	19.7	50	101.3	8.4	8.3	11.1
149	Inabenfide	0.9976	0.1-50	10	72.6	9.0	12.4	50	83.1	7.8	9.4	-20.0
150	Indoxacarb	0.9988	0.1-50	10	78.6	10.1	12.8	50	73.5	4.0	5.5	-21.3
151	Ipconazole	0.9987	0.1-25	10	70.0	11.5	16.4	50	78.3	6.9	8.8	-14.8
152	Iprobenfos	0.9986	0.5-25	5	91.9	16.2	17.6	50	76.7	5.8	7.5	3.5
153	Iprovalicarb	0.9982	0.1-50	10	90.0	2.8	3.1	50	91.4	2.0	2.1	-25.2
154	Isoprocarb	0.9995	0.25-25	2.5	98.1	15.0	15.3	50	81.9	5.7	6.9	-10.4
155	Isoprothiolane	0.9985	0.25-25	10	83.7	3.8	4.6	50	74.8	2.0	2.7	-2.4
156	Isoproturon	0.9980	0.1-25	10	91.4	5.7	6.3	50	74.7	10.6	14.2	25.6
157	Isopyrazam	0.9986	0.1-50	10	75.9	4.8	6.4	50	83.7	7.9	9.4	-27.9
158	Isoxaben	0.9924	0.1-50	10	77.5	11.5	14.8	50	91.1	4.8	5.3	-27.5
159	Isoxathion	0.9974	0.25-50	10	100.6	19.3	19.2	50	78.7	7.1	9.0	-13.0
160	Linuron	0.9989	0.1-50	5	74.8	12.4	16.5	50	77.7	7.6	9.8	-38.1
161	Lufenuron	0.9959	0.25-50	5	81.3	4.9	6.0	50	75.5	2.6	3.5	-29.8
162	Malaoxon	0.9978	0.1-50	10	83.2	3.5	4.2	50	89.8	5.5	6.1	-20.1
163	Mandestrobin	0.9996	0.1-50	5	86.6	16.7	19.3	50	82.5	5.8	7.0	-23.8
164	Mandipropamid	0.9975	0.1-50	10	79.8	2.0	2.6	50	90.0	2.1	2.3	-33.7
165	Mefenacet	0.9991	0.1-50	10	79.2	6.1	7.7	50	73.7	7.0	9.6	-23.6
166	Mefentrifluconazole	0.9825	0.25-25	10	71.4	13.6	19.1	50	114.6	15.6	13.6	-31.1

167	Mephosfolan	0.9989	0.1-50	10	79.7	3.9	4.9	50	89.7	7.3	8.2	-26.9
168	Mepronil	0.9982	0.1-25	5	76.2	7.5	9.9	50	76.0	5.3	6.9	-18.8
169	Mesotrione	0.9950	0.25-50	2.5	118.4	9.5	8.0	50	76.3	0.7	0.9	250.5
170	Metaflumizone (E)	0.9988	0.5-50	2.5	110.9	9.3	8.4	50	81.6	6.9	8.4	-27.6
171	Metaflumizone (Z)	0.9978	0.1-50	5	81.1	15.3	18.9	50	73.9	3.1	4.2	-11.5
172	Metalaxyl	0.9974	0.1-50	10	79.4	10.9	13.8	50	84.9	4.4	5.1	-15.3
173	Metamifop	0.9995	0.1-25	10	73.8	3.9	5.4	50	72.9	4.0	5.5	-12.3
174	Metamitron	0.9990	0.5-25	10	90.7	2.0	2.2	50	79.1	3.0	3.8	-9.8
175	Metazosulfuron	0.9924	0.1-50	10	75.7	7.1	9.4	50	87.3	11.0	12.6	-15.8
176	Metconazole	0.9979	0.1-50	5	72.7	1.2	1.7	50	76.7	10.3	13.4	-17.2
177	Methabenzthiazuron	0.9989	0.1-50	10	71.0	6.5	9.2	50	72.1	9.1	12.6	-23.1
178	Methiocarb-sulfone	0.9997	0.1-50	5	76.3	2.8	3.7	50	88.6	2.8	3.2	-31.1
179	Methiocarb-sulfoxide	0.9968	0.1-50	10	75.5	1.9	2.6	50	84.3	2.3	2.7	-19.8
180	Methoprotryne	0.9997	0.1-50	2.5	75.0	6.2	8.3	50	76.9	13.7	17.9	-38.8
181	Methoxyfenozide	0.9923	0.1-25	5	70.0	12.6	18.0	50	77.3	12.0	15.5	15.3
182	Metobromuron	0.9962	0.1-50	5	116.8	11.7	10.0	50	76.0	6.0	7.9	-3.8
183	Metolcarb	0.9993	0.1-50	10	77.7	2.8	3.6	50	85.6	5.7	6.7	-29.1
184	Metominostrboin (Z)	0.9995	0.1-50	1	83.2	8.3	10.0	50	83.1	7.7	9.2	-24.8
185	Metominostrobin (E)	0.9992	0.1-50	5	78.9	3.3	4.2	50	92.0	7.9	8.6	-29.3

186	Metrafenon	0.9966	0.25-50	2.5	95.9	8.9	9.2	50	78.1	7.6	9.7	-17.3
187	Metrafenone	0.9948	0.25-50	2.5	86.9	11.3	13.0	50	76.7	2.5	3.3	-15.0
188	Mevinphos	0.9959	0.5-50	5	114.6	3.6	3.1	50	94.8	2.6	2.8	-12.1
189	Molinate	0.9961	1-50	10	112.3	21.6	19.2	50	76.7	11.1	14.4	-10.4
190	Monocrotophos	0.9989	0.25-25	2.5	72.6	0.7	1.0	50	77.0	3.6	4.7	-19.3
191	Monolinuron	0.9995	0.1-50	2.5	99.2	2.0	2.0	50	79.5	5.6	7.1	-22.2
192	Napropamide	0.9963	0.1-50	10	73.0	7.4	10.1	50	90.3	5.0	5.6	-27.0
193	Neburon	0.9940	0.25-50	10	93.4	15.1	16.2	50	105.5	4.6	4.4	-13.5
194	Nicosulfuron	0.9997	0.1-50	5	93.9	1.9	2.1	50	79.8	10.0	12.6	-15.1
195	Nitenpyram	0.9964	0.5-50	5	102.1	2.9	2.8	50	75.8	1.7	2.2	-3.6
196	Norflurazon	0.9991	0.1-50	5	75.5	1.3	1.8	50	90.2	7.0	7.8	-23.7
197	Nuarimol	0.9991	0.1-50	5	72.7	13.5	18.5	50	85.1	7.3	8.6	-12.0
198	Ofurace	0.9985	0.1-50	5	71.4	2.8	3.9	50	91.8	2.9	3.2	-31.5
199	Orysastrobin	0.9992	0.25-25	1	99.8	10.1	10.2	50	86.7	10.2	11.7	-2.1
200	Oxadixyl	0.9998	0.1-50	2.5	80.1	5.3	6.7	50	88.7	3.4	3.8	-25.1
201	Oxamyl	0.9996	0.1-50	1	95.9	7.1	7.5	50	79.0	3.8	4.8	-22.1
202	Oxathiapiprolin	0.9980	0.1-50	10	88.0	12.0	13.6	50	87.2	5.9	6.8	-22.2
203	Oxaziclomefone	0.9960	1-50	10	104.3	7.5	7.2	50	71.1	5.0	7.0	-22.3
204	Oxycarboxin	0.9972	0.1-50	10	83.3	1.6	1.9	50	89.6	1.1	1.2	-18.4

205	Oxydemeton-methyl	0.9999	0.1-50	10	75.8	1.2	1.6	50	74.5	3.3	4.4	-23.8
206	Paraoxon-methyl	0.9996	0.25-25	2.5	72.3	2.1	2.9	50	77.6	2.6	3.3	4.5
207	Pencycuron	0.9958	0.25-50	2.5	111.7	10.1	9.1	50	79.9	4.2	5.3	-25.6
208	Penoxsulam	0.9984	0.1-50	10	73.4	13.9	18.9	50	81.9	6.3	7.7	3.8
209	Phorate	0.9987	0.1-50	10	75.8	9.0	11.9	50	80.3	3.4	4.3	-26.6
210	Phorate-oxon	0.9959	0.1-25	10	88.7	11.7	13.1	50	91.1	3.8	4.2	-28.7
211	Phorate-oxon-sulfone	0.9981	0.1-50	10	74.2	3.8	5.1	50	85.7	4.4	5.2	-28.2
212	Phorate-oxon-sulfoxide	0.9997	0.1-50	1	89.6	6.0	6.7	50	83.4	5.2	6.3	-24.0
213	Phorate-sulfone	0.9963	1-50	5	101.9	8.3	8.1	50	85.3	0.8	1.0	-14.0
214	Phorate-sulfoxide	0.9957	0.1-50	10	78.0	1.8	2.4	50	95.6	10.1	10.6	-27.7
215	Phosalone	0.9949	0.25-50	5	84.1	4.4	5.3	50	89.4	7.2	8.1	-3.3
216	Phosfolan	0.9994	0.1-50	5	72.7	1.1	1.5	50	85.1	3.1	3.6	-21.1
217	Phosmet-oxon	0.9992	0.1-50	5	73.5	4.6	6.3	50	89.1	2.7	3.1	-23.2
218	Phosphamidon	0.9998	0.1-50	5	83.2	4.7	5.6	50	87.3	6.7	7.7	-31.2
219	Picarbutrazox	0.9995	0.1-25	5	86.6	7.4	8.5	50	74.8	9.5	12.7	3.5
220	Picoxystrobin	0.9998	0.1-25	1	96.2	9.7	10.1	50	83.0	3.0	3.6	-0.5
221	Pinoxaden	0.9941	1-50	5	79.2	5.2	6.5	50	70.1	0.3	0.4	7.3
222	Piperonyl butoxide	0.9925	0.25-50	10	107.3	3.4	3.1	50	75.1	3.9	5.2	-23.6
223	Piperophos	0.9995	0.1-25	5	70.0	6.5	9.4	50	70.0	6.1	8.7	-16.0

224	Pirimicarb	0.9975	0.1-50	10	76.8	4.7	6.2	50	85.6	8.1	9.5	-32.9
225	Pirimicarb-desmethyl	0.9988	0.1-50	5	73.0	3.9	5.4	50	84.7	4.3	5.1	-26.5
226	Pirimiphos-methyl	0.9961	0.25-50	5	96.6	14.7	15.2	50	70.5	9.2	13.1	-21.1
227	Probenazole	0.9994	0.5-25	5	74.6	3.7	5.0	50	89.2	7.6	8.5	-8.5
228	Prochloraz	0.9930	0.1-50	10	86.1	5.1	6.0	50	79.3	3.0	3.7	-28.5
229	Promecarb	0.9994	0.1-50	10	83.0	11.5	13.9	50	81.6	5.1	6.2	-10.7
230	Prometryn	0.9970	0.1-50	5	92.7	17.7	19.0	50	73.2	13.7	18.7	-6.0
231	Pronamide (Propyzamide)	0.9979	0.1-25	10	78.9	8.1	10.2	50	78.5	9.5	12.0	-9.1
232	Propachlor	0.9962	0.1-25	5	73.2	13.8	18.8	50	77.5	3.9	5.1	-0.3
233	Propanil	0.9923	0.25-50	10	88.1	10.7	12.2	50	90.2	8.3	9.2	-26.3
234	Propazine	0.9988	0.1-50	10	90.3	15.2	16.9	50	76.7	7.1	9.2	-30.3
235	Propoxur	0.9950	0.1-50	10	77.4	4.0	5.2	50	88.1	1.0	1.1	-27.0
236	Prosulfocarb	0.9985	1-50	10	108.1	5.7	5.3	50	70.1	6.2	8.8	-16.1
237	Prothioconazole-desthio	0.9980	0.25-25	10	72.7	14.4	19.8	50	79.6	3.6	4.6	6.4
238	Pydiflumetofen	0.9995	0.1-25	5	83.6	8.1	9.7	50	78.8	7.8	9.9	-9.3
239	Pyflubumide	0.9959	0.1-50	10	71.1	8.1	11.4	50	80.9	5.9	7.3	-33.3
240	Pyflubumide-NH	0.9984	0.1-50	10	83.6	15.9	19.0	50	89.6	4.9	5.5	-22.4
241	Pyracarbolid	0.9940	0.1-50	10	75.7	6.2	8.1	50	86.3	5.0	5.8	-20.2
242	Pyraclofos	0.9995	0.1-25	1	79.1	13.4	16.9	50	71.8	5.0	7.0	6.0

243	Pyraclonil	0.9974	0.1-50	10	82.4	13.2	16.1	50	96.3	8.1	8.4	-15.4
244	Pyraclostrobin	0.9981	0.1-25	1	95.8	7.8	8.2	50	75.8	4.9	6.5	-15.1
245	Pyraflufen-ethyl	0.9986	0.1-25	5	71.9	5.2	7.2	50	87.1	13.7	15.8	3.3
246	Pyrazolate	0.9977	0.1-25	5	70.8	1.8	2.5	50	70.4	7.0	10.0	91.5
247	Pyrazophos	0.9986	0.1-50	5	71.0	2.3	3.2	50	74.1	3.8	5.1	-9.3
248	Pyrazoxyfen	0.9989	0.5-25	2.5	75.9	13.8	18.1	50	77.7	9.6	12.3	8.9
249	Pyribencarb	0.9983	0.1-50	10	78.2	11.8	15.1	50	83.9	11.5	13.8	-32.3
250	Pyridaphenthion	0.9995	0.25-25	5	95.9	9.8	10.2	50	84.6	4.6	5.4	0.6
251	Pyriftalid	0.9993	0.1-50	5	72.3	9.2	12.7	50	77.4	8.0	10.3	-25.9
252	Pyrimethanil	0.9967	1-50	5	110.9	14.0	12.6	50	70.9	9.7	13.7	-27.5
253	Pyrimisulfan	0.9975	0.1-50	10	80.9	14.9	18.4	50	90.9	2.5	2.8	-6.3
254	Pyriofenone	0.9963	0.25-50	5	115.6	6.6	5.7	50	77.8	3.0	3.8	-17.2
255	Pyroquilon	0.9977	0.1-50	10	76.2	9.8	12.9	50	87.8	2.8	3.2	-36.3
256	Quinoclamine	0.9997	1-50	10	71.9	4.5	6.2	50	75.5	6.8	9.0	-30.2
257	Quizalofop-ethyl	0.9967	0.25-50	5	114.0	1.0	0.9	50	73.3	4.4	6.0	-16.1
258	Saflufenacil	0.9985	0.1-50	10	85.2	11.4	13.4	50	76.6	11.9	15.6	14.5
259	Secbumeton	0.9985	0.1-50	10	77.7	2.4	3.1	50	88.4	9.0	10.1	-24.9
260	Sedaxane_cis	0.9940	0.1-25	10	83.0	8.3	10.0	50	86.4	5.2	6.0	-4.5
261	Sedaxane_trans	0.9919	0.1-50	10	88.9	4.6	5.2	50	100.3	11.2	11.1	-31.1

262	Sethoxydim	0.9976	0.5-50	5	107.0	13.0	12.2	50	71.7	6.0	8.4	-18.9
263	Simetryn	0.9995	0.1-25	2.5	71.8	2.6	3.6	50	78.9	4.4	5.6	-0.1
264	Spinetoram (J)	0.9985	0.1-50	2.5	97.9	11.9	12.2	50	80.7	5.1	6.4	-28.9
265	Tebuconazole	0.9950	0.1-50	10	86.1	10.4	12.1	50	81.0	9.7	11.9	-21.4
266	Tebufenpyrad	0.9970	0.5-50	5	101.0	9.5	9.4	50	76.2	10.4	13.7	-21.9
267	Tebufloquin	0.9982	0.1-50	10	70.8	13.1	18.5	50	78.3	5.6	7.1	-25.2
268	Tebuthiuron	0.9988	0.1-50	1	77.5	7.3	9.4	50	96.8	8.3	8.6	-34.6
269	Teflubenzuron	0.9964	0.25-50	2.5	114.5	20.4	17.8	50	72.4	6.2	8.5	-17.0
270	Tepraloxydim	0.9917	0.5-25	10	104.5	10.1	9.6	50	75.5	10.7	14.2	5.0
271	Terbufos-oxon	0.9941	0.25-50	5	101.1	11.4	11.3	50	84.1	5.7	6.8	-12.6
272	Terbufos-oxon-sulfone	0.9986	0.1-50	5	75.4	4.6	6.2	50	89.3	3.4	3.8	-23.8
273	Terbufos-oxon-sulfoxide	0.9996	0.1-50	5	76.2	1.2	1.6	50	83.7	4.1	4.9	-22.6
274	Terbufos-sulfoxide	0.9991	0.1-50	10	92.5	5.6	6.0	50	90.0	6.7	7.5	-36.8
275	Terbuthylazine	0.9986	0.1-50	10	72.1	7.5	10.3	50	80.3	6.8	8.4	-24.2
276	Terbutryn	0.9973	0.1-25	10	70.0	6.0	8.5	50	74.9	10.5	14.0	-8.0
277	Tetrachlorvinphos	0.9993	0.1-50	10	72.4	3.0	4.2	50	87.9	17.3	19.7	-28.1
278	Tetraconazole	0.9977	0.1-25	5	93.2	11.0	11.8	50	80.2	6.8	8.4	-0.3
279	Thenylchlor	0.9973	0.1-50	2.5	107.8	15.6	14.5	50	76.5	5.3	6.9	-8.0
280	Thiabendazole	0.9993	0.1-50	5	72.0	4.4	6.1	50	75.4	5.0	6.6	-34.5

281	Thiacloprid	0.9988	0.1-50	5	75.7	4.8	6.3	50	88.0	6.8	7.7	-23.9
282	Thiamethoxam	0.9985	0.1-50	10	78.2	2.7	3.4	50	81.7	3.3	4.0	-28.9
283	Thiazopyr	0.9974	0.1-50	10	73.4	7.4	10.1	50	82.4	3.1	3.8	-18.9
284	Thidiazuron	0.9962	0.25-50	5	109.5	6.4	5.9	50	93.5	1.5	1.6	-18.4
285	Thidiazuron	0.9983	0.25-50	10	90.3	15.5	17.1	50	83.7	5.7	6.8	-12.1
286	Thifensulfuron-methyl	0.9991	2.5-50	10	70.7	9.0	12.8	50	78.4	3.2	4.1	-20.5
287	Thionazin	0.9909	0.25-50	10	96.1	14.3	14.9	50	102.4	4.7	4.6	-14.5
288	Tolfenpyrad	0.9981	1-50	10	118.6	9.9	8.4	50	70.4	8.8	12.5	-8.2
289	Triadimefon	0.9995	0.1-25	10	89.2	11.1	12.4	50	71.3	2.1	3.0	9.7
290	Triafamone	0.9996	0.1-50	2.5	74.8	7.1	9.6	50	89.5	0.9	1.1	-24.1
291	Triazamate	0.9998	0.1-50	5	70.7	7.8	11.0	50	77.3	2.6	3.3	-14.2
292	Triazophos	0.9970	0.1-25	5	70.8	2.2	3.1	50	80.1	3.3	4.1	-17.6
293	Tricyclazole	0.9945	0.1-50	10	79.4	13.8	17.4	50	80.6	9.8	12.1	-27.6
294	Trifloxystrobin	0.9980	0.1-50	10	74.6	12.9	17.3	50	77.7	5.9	7.5	-31.7
295	Triflumizole	0.9985	0.1-25	2.5	72.4	13.8	19.1	50	73.1	4.9	6.6	-9.1
296	Triflumuron	0.9972	0.25-50	5	113.0	22.2	19.6	50	80.9	3.8	4.8	-9.4
297	Triticonazole	0.9989	0.1-25	2.5	92.5	5.1	5.6	50	81.3	12.5	15.3	-11.6
298	TZ-1E	0.9972	0.1-25	10	72.7	6.4	8.8	50	86.6	9.8	11.3	-15.6
299	Vamidothion	0.9990	0.1-50	5	71.4	3.2	4.4	50	84.7	4.0	4.7	-22.7

300	XMC	0.9999	0.1-50	2.5	99.6	17.0	17.0	50	79.5	6.2	7.9	-21.2
301	Zoxamide	0.9914	0.25-50	2.5	84.3	6.3	7.4	50	86.1	7.3	8.5	-12.6

SD: Standard Deviation

RSD: Relative Standard Deviation

Table S6. Method validation data for pesticide residue analysis in mung bean

No.	Compound name	r^2	Linear range (μg/kg)	Sample preparation method 3 (type 2 d-SPE sorbent)								
				Low (LOQ) (μg/kg)	Recovery %		RSD %	High (μg/kg)	Recovery %		RSD %	Matrix effect %
1	2,3,5-trimethacarb	0.9977	0.1-50	2.5	111.0	16.8	15.2	50	93.7	8.5	9.0	-17.1
2	3,4,5-trimethacarb	0.9977	0.1-50	5	75.0	5.2	6.9	50	96.3	8.0	8.3	-13.7
3	3-hydroxycarbofuran	0.9972	0.1-50	2.5	112.9	10.2	9.1	50	83.8	6.7	8.0	-19.9
4	6-Benzyl aminopurine	0.9977	0.1-50	2.5	92.3	8.8	9.6	50	75.7	7.6	10.1	-19.3
5	Acetamiprid	0.9974	0.1-50	1	119.1	9.8	8.2	50	82.8	5.7	6.8	-17.8
6	Acetochlor	0.9906	0.1-25	5	94.1	5.6	6.0	50	90.5	18.1	20.0	-3.1
7	Acibenzolar-S-methyl	0.9933	1-50	10	85.1	6.7	7.9	50	74.1	4.8	6.4	-13.5
8	Alachlor	0.9963	0.1-50	10	85.2	9.3	10.9	50	99.3	11.5	11.5	-21.8
9	Aldicarb	0.9970	0.1-50	2.5	102.4	4.8	4.7	50	88.6	7.7	8.7	-18.4
10	Alidcarb sulfone	0.9974	0.1-50	2.5	110.0	2.3	2.1	50	83.0	9.0	10.9	-23.6
11	Ametoctradin	0.9972	0.1-50	10	89.0	6.2	7.0	50	97.3	9.8	10.1	-25.1
12	Anilofos	0.9963	0.1-50	2.5	108.4	6.0	5.6	50	96.8	4.5	4.7	-20.1
13	Aramite	0.9977	0.1-50	2.5	92.9	12.4	13.4	50	87.4	5.2	6.0	-11.7
14	Aspon	0.9979	0.1-50	2.5	81.6	3.7	4.5	50	81.4	6.0	7.3	-24.6
15	Atrazine	0.9950	0.1-50	2.5	114.9	8.8	7.6	50	88.5	6.5	7.3	-11.9

16	Azaconazole	0.9969	0.1-50	5	113.6	17.8	15.6	50	81.4	6.4	7.9	-5.9
17	Azamethiphos	0.9961	0.1-50	2.5	111.5	2.1	1.9	50	86.8	7.5	8.6	-22.0
18	Azimsulfuron	0.9942	0.1-50	10	110.0	3.8	3.5	50	90.6	7.6	8.4	-13.7
19	Azoxystrobin	0.9916	0.1-50	5	106.4	6.8	6.4	50	91.3	9.0	9.9	-2.1
20	Benalaxyl	0.9941	0.1-50	5	74.8	2.1	2.9	50	95.2	12.0	12.6	-23.7
21	Bendiocarb	0.9977	0.1-50	2.5	103.8	5.8	5.6	50	96.0	8.5	8.9	-23.1
22	Benfuracarb	0.9928	0.1-50	10	74.6	12.5	16.8	50	87.2	16.3	18.7	-28.4
23	Benodanil	0.9974	0.1-50	5	96.3	6.7	6.9	50	97.4	8.2	8.4	-16.8
24	Bensulfuron-methyl	0.9974	0.1-50	1	85.6	11.0	12.9	50	87.0	8.3	9.5	-9.1
25	Benzobicyclon	0.9967	0.1-50	10	106.1	20.6	19.4	50	91.3	5.0	5.5	-20.7
26	Benzoximate	0.9955	0.1-50	2.5	84.7	11.4	13.5	50	96.3	11.4	11.9	-35.5
27	Benzoylprop-ethyl	0.9956	0.1-50	5	74.3	9.0	12.1	50	93.2	11.1	12.0	-16.8
28	Bistrifluron	0.9967	0.1-50	2.5	109.6	9.5	8.6	50	85.8	6.8	7.9	-19.5
29	Bixafen	0.9942	0.1-50	10	113.5	17.4	15.3	50	117.9	6.2	5.2	-25.8
30	Boscalid	0.9930	0.1-50	10	118.6	2.5	2.1	50	89.2	4.9	5.5	-9.1
31	Bromacil	0.9964	0.1-50	5	114.1	4.7	4.2	50	93.2	7.7	8.2	-23.2
32	Bromobutide	0.9956	0.1-50	5	102.7	16.5	16.1	50	88.9	13.4	15.1	-5.9
33	Bupirimate	0.9983	0.1-50	2.5	107.5	13.7	12.8	50	85.9	11.3	13.1	-8.6
34	Buprofezin	0.9976	0.1-50	1	102.4	3.4	3.3	50	78.9	6.5	8.2	-29.5

35	Cadusafos	0.9976	0.1-50	2.5	80.0	10.0	12.5	50	83.8	4.6	5.5	-15.4
36	Cafenstrole	0.9906	0.1-50	10	98.2	11.7	11.9	50	91.2	9.7	10.6	-8.1
37	Carbaryl	0.9968	0.1-50	2.5	95.4	7.6	8.0	50	91.9	11.5	12.6	-17.7
38	Carbendazim	0.9957	0.1-50	10	77.7	1.1	1.4	50	74.7	11.2	14.9	-34.3
39	Carbetamide	0.9979	0.1-50	2.5	112.5	8.2	7.3	50	92.5	9.6	10.3	-23.2
40	Carbofuran	0.9944	0.1-50	5	78.1	3.0	3.9	50	103.9	14.3	13.8	-22.9
41	Carbofuran-3-hydroxy	0.9977	0.1-50	2.5	103.5	8.5	8.2	50	87.2	7.4	8.4	-23.8
42	Carboxin	0.9923	0.1-50	5	114.0	7.5	6.6	50	82.6	9.3	11.3	-14.7
43	Carpropamid	0.9935	0.1-50	10	90.9	13.7	15.1	50	99.1	14.3	14.4	-16.9
44	Chlorbenzuron	0.9945	0.1-50	10	98.9	3.1	3.1	50	100.8	16.4	16.3	-24.2
45	Chlorfluazuron	0.9950	0.1-50	10	107.0	4.9	4.6	50	86.1	7.9	9.1	-20.5
46	Chloridazon	0.9969	0.1-50	5	105.0	10.3	9.8	50	84.7	6.9	8.2	-25.1
47	Chlorimuron-ethyl	0.9957	0.1-50	5	116.7	9.3	8.0	50	92.4	11.8	12.8	-14.7
48	Chlorotoluron	0.9972	0.1-50	1	74.1	7.4	10.0	50	95.0	7.5	7.9	-21.4
49	Chloroxuron	0.9959	0.1-50	10	93.2	1.7	1.8	50	99.0	12.4	12.5	-19.6
50	Chlorpyrifos	0.9980	0.1-50	5	74.7	5.1	6.9	50	76.0	5.6	7.3	-23.6
51	Chlorpyrifos-methyl	0.9947	0.1-50	10	119.1	15.6	13.1	50	78.5	9.9	12.6	-11.7
52	Clethodim	0.9980	0.1-50	10	105.5	20.8	19.7	50	89.4	16.0	17.9	-20.9
53	Clofentezine	0.9913	0.1-50	10	81.8	6.7	8.2	50	85.2	3.6	4.2	-23.4

54	Clomazone	0.9962	0.1-50	5	88.5	3.9	4.4	50	91.4	6.5	7.1	-22.1
55	Clomeprop	0.9919	0.1-25	2.5	88.1	10.8	12.3	50	79.5	1.8	2.2	7.0
56	Clothianidin	0.9961	0.1-50	5	99.8	10.1	10.1	50	86.9	11.3	13.0	-16.7
57	Crotoxyphos	0.9962	0.1-50	5	81.5	8.5	10.4	50	73.6	13.1	17.8	-7.8
58	Cyanazine	0.9968	0.1-50	2.5	101.4	11.5	11.3	50	91.5	7.8	8.6	-15.4
59	Cyazofamid	0.9959	0.1-50	10	82.1	8.2	10.0	50	100.5	9.8	9.8	-21.0
60	Cyclosulfamuron	0.9931	0.1-50	5	119.2	10.4	8.7	50	85.9	9.4	11.0	-7.7
61	Cyenopyrafen	0.9976	0.1-50	2.5	96.7	5.8	6.0	50	86.0	5.9	6.9	-25.3
62	Cyflufenamid	0.9942	0.1-50	10	111.2	11.1	10.0	50	96.2	5.1	5.3	-14.2
63	Cyflumetofen	0.9922	0.1-50	2.5	92.4	8.6	9.3	50	95.6	12.3	12.9	-8.3
64	Cymoxanil	0.9952	0.1-50	1	106.6	13.1	12.3	50	96.2	9.5	9.8	-25.6
65	Daimuron	0.9967	0.1-50	2.5	81.4	3.9	4.8	50	97.0	0.8	0.9	-20.8
66	Demeton-O	0.9949	0.1-50	10	103.3	15.9	15.4	50	101.1	18.5	18.3	-22.6
67	Demeton-S-methyl	0.9952	0.1-50	2.5	117.5	17.7	15.1	50	100.7	16.5	16.3	-11.4
68	Demeton-S-methyl sulfoxide	0.9969	0.1-50	2.5	107.6	3.3	3.0	50	79.6	9.5	12.0	-23.6
69	Demeton-S-methyl-sulfone	0.9966	0.1-50	5	111.5	3.0	2.7	50	83.5	8.0	9.6	-20.4
70	Demeton-S-sulfone	0.9965	0.1-50	5	112.3	3.4	3.1	50	87.9	6.3	7.2	-21.4
71	Demeton-S-sulfoxide	0.9975	0.1-50	2.5	112.9	9.0	8.0	50	88.4	8.3	9.4	-20.0
72	Desmetryn	0.9972	0.1-50	1	92.2	16.1	17.5	50	85.2	7.0	8.2	-20.2

73	Diazinon	0.9922	0.1-50	5	119.8	9.8	8.2	50	83.6	6.9	8.3	-13.4
74	Diclosulam	0.9930	0.5-50	10	92.4	7.6	8.2	50	78.5	11.2	14.2	-17.0
75	Dicrotophos	0.9947	0.1-50	5	107.1	2.1	2.0	50	80.7	7.6	9.4	-20.8
76	Diethatyl-ethyl	0.9966	0.1-50	5	72.0	4.1	5.8	50	95.8	13.6	14.2	-17.9
77	Diethofencarb	0.9913	0.1-25	5	98.6	4.6	4.6	50	103.9	7.9	7.6	-4.5
78	Diflufenican	0.9980	0.1-50	5	98.7	16.4	16.6	50	93.9	9.8	10.5	-25.8
79	Dimethachlor	0.9981	0.1-50	2.5	100.5	12.1	12.0	50	102.8	12.7	12.4	-19.2
80	Dimethametryn	0.9934	0.1-50	10	100.0	11.3	11.3	50	89.0	7.4	8.3	-20.4
81	Dimethenamide	0.9976	0.1-50	2.5	78.7	14.6	18.5	50	100.1	8.2	8.2	-17.9
82	Dimethoate	0.9973	0.1-50	2.5	107.3	3.1	2.9	50	89.3	6.3	7.0	-21.6
83	Diniconazole	0.9912	0.1-50	2.5	106.1	17.7	16.7	50	86.2	15.5	18.0	-16.9
84	Diphenamid	0.9928	0.1-50	10	92.6	16.6	17.9	50	105.6	11.5	10.9	-34.4
85	Disulfoton-sulfone	0.9957	0.1-50	5	85.3	16.7	19.6	50	102.5	11.7	11.4	-23.9
86	Disulfoton-sulfoxide	0.9969	0.1-50	10	118.7	8.2	6.9	50	92.5	6.7	7.3	-8.9
87	Dithiopyr	0.9953	0.1-50	10	95.6	6.7	7.0	50	94.6	5.4	5.7	-15.4
88	Diuron	0.9960	0.1-50	10	96.8	7.8	8.1	50	96.3	2.4	2.5	-30.5
89	Edifenphos	0.9969	0.1-50	2.5	77.9	10.9	14.0	50	86.8	7.9	9.1	-14.2
90	Epoxiconazole	0.9942	0.1-50	1	119.8	21.9	18.3	50	83.6	12.1	14.5	-6.0
91	Esprocarb	0.9977	0.1-50	2.5	105.1	6.9	6.5	50	81.1	5.3	6.6	-19.3

92	Etaconazole	0.9931	0.1-50	10	93.4	16.0	17.1	50	102.1	12.9	12.6	-26.8
93	Ethaboxam	0.9949	0.1-50	2.5	90.9	7.8	8.6	50	98.8	7.9	8.0	-15.8
94	Ethirimol	0.9973	0.1-50	2.5	103.0	5.5	5.3	50	78.7	5.2	6.6	-24.6
95	Etoxazole	0.9977	0.1-50	2.5	97.0	3.5	3.6	50	84.2	6.9	8.2	-24.0
96	Etrimfos	0.9971	0.1-50	2.5	75.1	2.1	2.8	50	90.7	7.8	8.6	-14.9
97	Famoxadone	0.9968	0.1-50	10	82.1	15.1	18.4	50	95.8	8.8	9.2	-21.6
98	Fenamiphos	0.9904	0.1-25	5	93.6	7.7	8.2	50	96.5	11.4	11.9	1.1
99	Fenamiphos sulfoxide	0.9961	0.1-50	5	88.5	14.2	16.0	50	89.8	5.0	5.6	-20.3
100	Fenamiphos-sulfone	0.9979	0.1-50	1	114.2	19.2	16.8	50	84.6	14.8	17.4	-18.0
101	Fenamiphos-sulfoxide	0.9919	0.1-50	10	81.6	9.0	11.0	50	91.3	9.5	10.4	-29.6
102	Fenarimol	0.9967	0.1-50	10	111.4	16.8	15.1	50	103.0	18.6	18.1	-20.2
103	Fenazaquin	0.9971	0.1-50	2.5	102.1	1.3	1.3	50	81.4	6.4	7.9	-29.7
104	Fenbuconazole	0.9931	0.5-50	10	100.1	12.3	12.3	50	89.7	11.1	12.4	-12.7
105	Fenfuram	0.9973	0.1-50	2.5	84.7	11.7	13.8	50	87.1	12.8	14.7	-26.2
106	Fenobucarb	0.9975	0.1-50	10	89.4	11.0	12.3	50	87.2	6.2	7.1	-20.9
107	Fenothiocarb	0.9973	0.1-50	5	102.3	19.5	19.1	50	84.4	0.7	0.8	-8.5
108	Fenoxaprop-ethyl	0.9984	0.1-50	1	90.1	12.1	13.5	50	83.5	6.2	7.4	-21.1
109	Fenpyroximate	0.9978	0.1-50	2.5	100.1	2.6	2.6	50	87.5	7.4	8.5	-23.4
110	Fenquinotrione_KIH-3635-M-2	0.9964	0.1-50	5	118.1	5.2	4.4	50	72.8	11.0	15.1	5.4

111	Fensulfothion	0.9913	0.1-25	1	114.5	2.0	1.8	50	84.2	5.6	6.6	1.3
112	Fenthion oxon	0.9974	0.1-50	5	92.6	9.5	10.3	50	90.1	4.8	5.3	-18.1
113	Fenthion oxon sulfone	0.9968	0.1-50	1	117.4	4.3	3.7	50	77.5	4.2	5.4	-23.1
114	Fenthion oxon sulfoxide	0.9973	0.1-50	1	112.0	16.7	14.9	50	87.4	5.6	6.5	-39.6
115	Fenthion sulfoxide	0.9973	0.1-50	1	72.6	8.3	11.4	50	97.4	14.6	15.0	-14.5
116	Fenthion-sulfone	0.9975	0.1-50	10	82.7	16.4	19.8	50	95.5	4.8	5.0	-24.8
117	Fipronil	0.9943	0.1-50	1	105.7	16.6	15.7	50	94.5	10.6	11.2	-5.5
118	Fipronil-sulfone	0.9971	0.1-50	2.5	85.7	10.4	12.1	50	91.1	4.9	5.4	-24.1
119	Flamprop-isopropyl	0.9948	0.1-50	10	87.1	10.9	12.5	50	99.9	14.9	14.9	-10.2
120	Flonicamid	0.9971	0.1-50	2.5	118.1	12.5	10.6	50	86.2	9.6	11.1	-26.4
121	Fluacrypyrim	0.9959	0.1-50	10	101.3	15.9	15.7	50	119.7	8.0	6.6	-27.2
122	Fluazinam	0.9974	0.1-50	2.5	100.0	15.2	15.1	50	83.5	5.0	6.0	-22.4
123	Flufenoxuron	0.9920	0.1-50	10	117.8	16.4	13.9	50	82.4	0.7	0.8	-10.9
124	Fluometuron	0.9935	0.1-50	10	113.3	18.8	16.6	50	90.0	5.8	6.5	-15.4
125	Fluopicolide	0.9963	0.1-50	5	86.4	11.2	12.9	50	88.7	12.6	14.2	-11.0
126	Fluopyram	0.9973	0.1-50	5	85.9	7.6	8.8	50	101.6	7.2	7.1	-22.3
127	Flupyradifurone	0.9967	0.1-50	2.5	111.0	9.1	8.2	50	84.0	6.4	7.6	-18.8
128	Flurochloridone	0.9954	0.25-50	5	79.3	7.2	9.0	50	91.4	12.4	13.6	-22.2
129	Flurtamone	0.9947	0.1-50	5	82.7	4.8	5.8	50	93.6	10.1	10.8	-36.4

130	Flusilazole	0.9956	0.1-50	5	116.6	10.3	8.8	50	81.0	7.9	9.7	-4.9
131	Flutianil	0.9975	0.1-50	2.5	103.7	15.3	14.8	50	89.0	3.1	3.5	-10.1
132	Flutolanil	0.9959	0.1-50	5	80.7	6.0	7.4	50	118.0	11.9	10.1	-22.8
133	Flutriafol	0.9963	0.1-50	2.5	115.4	14.1	12.3	50	93.3	6.5	7.0	-19.9
134	Fluxametamide	0.9968	0.1-50	10	113.4	13.5	11.9	50	100.1	9.1	9.1	-11.4
135	Fluxapyroxad	0.9953	0.1-50	10	99.1	4.7	4.8	50	114.5	6.8	5.9	-23.4
136	Forchlorfenuron	0.9979	0.1-50	5	107.0	11.6	10.9	50	90.5	15.8	17.4	-19.5
137	Fosthiazate	0.9972	0.1-50	2.5	106.9	13.3	12.5	50	87.4	8.7	10.0	-19.5
138	Furathiocarb	0.9921	0.1-25	10	73.0	2.6	3.6	50	70.2	3.8	5.4	-8.2
139	Halosulfuron-methyl	0.9962	0.1-50	2.5	113.4	3.7	3.2	50	84.4	8.6	10.2	-4.5
140	Haloxyfop	0.9905	0.1-50	10	119.5	9.3	7.7	50	94.4	16.5	17.5	-22.6
141	Heptenophos	0.9933	0.1-50	2.5	104.4	9.3	8.9	50	89.4	5.8	6.5	-19.9
142	Hexaflumuron	0.9960	0.1-50	10	107.5	11.2	10.4	50	101.0	19.7	19.5	-18.4
143	Hexazinone	0.9969	0.1-50	1	102.8	1.9	1.9	50	95.1	5.0	5.3	-10.6
144	Hexythiazox	0.9978	0.1-50	5	89.0	7.0	7.9	50	85.1	3.5	4.1	-27.9
145	Imazalil	0.9944	0.1-50	5	107.5	21.3	19.8	50	86.3	11.2	12.9	-17.5
146	Imazethapyr	0.9976	0.1-50	2.5	95.2	7.2	7.6	50	71.4	7.0	9.9	-22.9
147	Imicyafos	0.9974	0.1-50	1	110.5	5.2	4.7	50	90.9	7.5	8.2	-24.2
148	Imidacloprid	0.9968	0.1-50	2.5	94.8	16.3	17.2	50	83.8	12.4	14.8	5.4

149	Inabenfide	0.9973	0.1-50	5	76.9	6.2	8.1	50	98.8	8.8	8.9	2.7
150	Ipconazole	0.9967	0.1-50	5	110.0	16.8	15.3	50	97.3	9.8	10.0	-7.8
151	Ipfencarbazone	0.9961	0.1-50	10	97.8	8.3	8.5	50	97.3	11.2	11.5	-20.9
152	Iprobenfos	0.9973	0.5-50	5	88.7	7.0	7.9	50	97.9	9.4	9.6	-18.7
153	Iprovalicarb	0.9974	0.1-50	1	114.5	16.2	14.2	50	95.6	18.1	19.0	-14.0
154	Isoprocarb	0.9964	0.25-50	10	93.4	9.9	10.6	50	84.6	3.0	3.6	-23.8
155	Isoprothiolane	0.9972	0.25-50	2.5	71.7	10.0	13.9	50	101.1	6.1	6.1	-19.1
156	Isoproturon	0.9975	0.1-50	2.5	70.3	12.6	17.9	50	84.6	10.0	11.8	-12.8
157	Isoxaben	0.9975	0.1-50	2.5	70.5	9.5	13.4	50	98.2	7.0	7.2	-22.1
158	Isoxathion	0.9925	0.1-50	5	70.6	0.2	0.2	50	92.9	14.4	15.5	-16.4
159	Linuron	0.9977	0.1-50	5	106.7	13.5	12.6	50	83.2	0.6	0.7	-17.3
160	Lufenuron	0.9926	0.1-50	5	84.9	13.2	15.6	50	97.9	17.0	17.3	8.2
161	Lufenuron	0.9978	0.1-50	5	92.0	17.0	18.5	50	102.8	14.4	14.0	-7.5
162	Malaoxon	0.9976	0.1-50	5	89.6	2.9	3.2	50	94.9	5.8	6.1	-19.8
163	Mandestrobin	0.9976	0.1-50	2.5	109.1	8.8	8.1	50	86.8	10.0	11.5	-16.6
164	Mandipropamid	0.9973	0.1-50	5	92.3	8.6	9.4	50	94.3	1.8	1.9	-13.7
165	Mefenacet	0.9964	0.1-50	10	97.8	7.1	7.3	50	95.7	15.7	16.4	-20.0
166	Mefentrifluconazole	0.9922	0.1-50	2.5	85.9	16.4	19.1	50	91.4	15.4	16.8	-21.4
167	Mepanipyrim	0.9926	0.1-50	5	79.2	11.0	13.9	50	94.2	4.9	5.2	-27.1

168	Mephosfolan	0.9961	0.1-50	5	110.8	8.3	7.5	50	92.1	5.4	5.9	-30.8
169	Mepronil	0.9965	0.1-50	5	98.4	6.6	6.7	50	97.2	8.5	8.7	-13.5
170	Mesotrione	0.9975	0.25-50	2.5	102.8	5.0	4.9	50	70.8	11.5	16.3	393.5
171	Metaflumizone (Z)	0.9908	0.1-25	2.5	109.1	7.6	7.0	50	100.3	11.5	11.5	-20.0
172	Metalaxyl	0.9959	0.1-50	1	79.5	4.6	5.8	50	90.2	10.4	11.5	-15.7
173	Metamifop	0.9912	0.1-50	5	102.9	4.3	4.2	50	89.6	4.2	4.7	-19.3
174	Metamitron	0.9961	0.5-50	5	110.3	0.6	0.5	50	81.2	7.6	9.3	-24.0
175	Metconazole	0.9926	0.1-50	10	84.2	13.3	15.8	50	89.4	17.7	19.8	-25.4
176	Methabenzthiazuron	0.9977	0.1-50	1	96.2	9.7	10.1	50	85.5	7.3	8.5	-22.9
177	Methiocarb-sulfone	0.9972	0.1-50	2.5	117.4	3.3	2.8	50	86.8	9.0	10.3	-24.8
178	Methiocarb-sulfoxide	0.9978	0.1-50	1	98.3	3.5	3.5	50	88.5	9.7	11.0	-20.3
179	Methoprotryne	0.9978	0.1-50	5	96.0	8.2	8.5	50	90.4	15.7	17.4	-21.1
180	Metobromuron	0.9929	0.1-25	1	73.5	5.6	7.7	50	92.7	6.1	6.5	-8.0
181	Metolcarb	0.9981	0.1-50	1	83.5	9.7	11.6	50	92.5	8.9	9.6	-27.3
182	Metominostrobin (E)	0.9973	0.1-50	5	89.7	5.6	6.2	50	97.3	9.3	9.5	-25.6
183	Metrafenon	0.9973	0.1-50	5	88.0	10.6	12.0	50	95.2	9.2	9.7	-26.9
184	Metrafenone	0.9971	0.1-50	2.5	72.9	2.5	3.4	50	96.6	10.2	10.6	-18.5
185	Mevinphos	0.9969	0.5-50	2.5	74.7	10.2	13.6	50	71.8	6.8	9.5	-28.8
186	Monocrotophos	0.9979	0.1-50	2.5	70.4	6.5	9.3	50	81.1	11.2	13.8	-28.1

187	Monolinuron	0.9973	0.1-50	2.5	98.9	15.1	15.3	50	97.0	11.6	12.0	-30.0
188	Neburon	0.9957	0.1-50	2.5	86.5	4.6	5.3	50	96.9	19.0	19.6	-14.1
189	Nitenpyram	0.9963	1-50	5	92.9	6.9	7.5	50	70.1	8.6	12.2	-20.0
190	Norflurazon	0.9980	0.1-50	1	98.7	18.2	18.5	50	93.7	5.9	6.3	-10.5
191	Noruron (Norea)	0.9957	0.1-50	5	71.4	6.6	9.3	50	91.2	9.1	9.9	-16.3
192	Nuarimol	0.9955	0.1-50	10	87.9	14.9	16.9	50	88.4	16.4	18.5	-4.8
193	Ofurace	0.9961	0.1-50	5	83.7	3.6	4.3	50	98.6	9.9	10.1	-29.2
194	Orysastrobin	0.9972	0.1-50	5	114.0	7.0	6.1	50	91.9	13.3	14.4	-19.4
195	Oxadiazon	0.9951	0.1-50	10	88.1	16.9	19.2	50	95.7	2.5	2.6	-32.9
196	Oxadixyl	0.9970	0.1-50	5	113.3	3.2	2.8	50	87.7	5.1	5.8	-20.6
197	Oxamyl	0.9956	0.1-50	2.5	113.5	4.0	3.6	50	79.5	8.2	10.3	-22.7
198	Oxathiapiprolin	0.9957	0.1-50	5	103.6	19.8	19.2	50	101.1	15.2	15.0	-7.9
199	Oxaziclomefone	0.9975	0.1-50	2.5	91.8	3.4	3.7	50	85.2	6.1	7.2	-22.8
200	Oxycarboxin	0.9977	0.1-50	1	106.1	2.3	2.2	50	88.5	8.9	10.1	-14.4
201	Oxydemeton-methyl	0.9968	0.1-50	2.5	110.7	4.3	3.9	50	80.0	9.7	12.1	-21.7
202	Pebulate	0.9951	0.1-50	10	102.2	14.5	14.2	50	79.9	10.1	12.6	-17.6
203	Pencycuron	0.9975	0.1-50	2.5	78.6	9.9	12.5	50	88.8	8.4	9.5	-22.3
204	Penoxsulam	0.9976	0.1-50	2.5	81.3	11.2	13.7	50	93.1	8.0	8.6	-13.5
205	Phenthoate	0.9945	0.1-50	5	92.3	16.8	18.2	50	114.9	11.4	9.9	-21.6

206	Phorate	0.9971	0.1-50	2.5	118.3	10.2	8.6	50	85.3	8.2	9.7	-19.8
207	Phorate-oxon	0.9965	0.1-50	1	119.1	23.7	19.9	50	94.7	14.8	15.6	-14.0
208	Phorate-oxon-sulfone	0.9979	0.1-50	2.5	98.7	8.6	8.7	50	82.5	7.5	9.1	-19.9
209	Phorate-oxon-sulfoxide	0.9970	0.1-50	2.5	117.8	16.1	13.6	50	85.2	11.5	13.5	-19.6
210	Phorate-sulfone	0.9971	0.1-50	2.5	84.0	7.1	8.5	50	91.4	3.5	3.8	-16.4
211	Phorate-sulfoxide	0.9965	0.1-50	2.5	104.3	11.0	10.6	50	93.2	12.6	13.5	-20.1
212	Phosalone	0.9952	0.1-50	10	119.4	9.9	8.3	50	98.8	5.4	5.4	-22.3
213	Phosfolan	0.9969	0.1-50	2.5	115.5	3.1	2.7	50	84.9	5.5	6.4	-21.5
214	Phosmet-oxon	0.9972	0.1-50	2.5	102.4	7.4	7.2	50	87.3	3.1	3.5	-20.2
215	Phosphamidon	0.9965	0.1-50	5	83.5	7.3	8.7	50	95.6	10.2	10.6	-29.4
216	Phoxim	0.9932	0.1-50	10	103.9	9.9	9.5	50	105.8	17.4	16.4	-16.2
217	Picolinafen	0.9947	0.1-50	10	89.0	5.8	6.5	50	92.6	10.7	11.6	-27.1
218	Picoxystrobin	0.9960	0.1-50	2.5	85.2	7.5	8.8	50	107.4	14.2	13.2	-23.0
219	Piperonyl butoxide	0.9972	0.1-50	2.5	112.2	19.8	17.7	50	87.0	12.9	14.8	-23.1
220	Piperophos	0.9966	0.1-50	5	89.5	4.8	5.4	50	92.8	16.7	18.0	-25.7
221	Pirimicarb	0.9961	0.1-50	5	103.5	0.7	0.7	50	89.6	10.0	11.1	-31.8
222	Pirimicarb-desmethyl	0.9968	0.1-50	2.5	113.1	3.7	3.3	50	86.9	7.3	8.4	-24.9
223	Pirimiphos-ethyl	0.9978	0.1-50	1	79.7	13.7	17.2	50	83.2	6.1	7.3	-23.1
224	Pirimiphos-methyl	0.9984	0.1-50	5	85.7	17.1	20.0	50	88.9	4.5	5.0	-28.6

225	Probenazole	0.9974	0.1-50	2.5	112.6	15.4	13.7	50	94.7	8.7	9.2	-19.6
226	Profenofos	0.9972	0.1-50	5	77.7	6.9	8.9	50	91.3	1.5	1.7	-22.7
227	Promecarb	0.9978	0.1-50	10	87.7	11.1	12.7	50	91.7	6.7	7.4	-18.5
228	Prometryn	0.9982	0.1-50	1	118.8	7.4	6.2	50	94.6	16.2	17.1	-18.0
229	Pronamide (Propyzamide)	0.9979	0.1-50	2.5	109.7	15.7	14.3	50	99.0	14.2	14.3	-20.7
230	Propachlor	0.9977	0.1-50	5	91.5	10.3	11.2	50	94.4	13.2	14.0	-20.9
231	Propaquizafop	0.9975	0.1-50	5	74.7	13.0	17.3	50	81.6	9.6	11.8	-19.5
232	Propargite	0.9971	0.1-50	5	70.3	2.0	2.8	50	84.9	3.2	3.8	-26.1
233	Propazine	0.9955	0.1-50	5	84.5	10.5	12.5	50	97.6	11.5	11.8	-18.5
234	Propoxur	0.9970	0.1-50	5	115.7	9.2	7.9	50	90.8	11.7	12.9	-23.9
235	Proquinazid	0.9978	0.1-50	2.5	73.9	2.5	3.4	50	71.1	2.6	3.6	-37.4
236	Prosulfocarb	0.9977	0.1-50	2.5	86.3	8.2	9.5	50	83.5	3.8	4.6	-17.7
237	Pydiflumetofen	0.9975	0.1-50	2.5	76.4	8.6	11.3	50	96.0	5.7	5.9	-26.2
238	Pyflubumide	0.9970	0.1-50	2.5	105.2	4.0	3.8	50	93.4	13.4	14.3	-8.9
239	Pyracarbolid	0.9963	0.1-50	5	71.6	7.5	10.4	50	93.0	6.2	6.7	-28.2
240	Pyraclofos	0.9962	0.1-50	10	96.3	11.2	11.7	50	99.4	3.4	3.4	-24.0
241	Pyraclonil	0.9973	0.1-50	10	97.3	9.3	9.5	50	89.4	11.4	12.8	-23.8
242	Pyraclostrobin	0.9986	0.1-50	5	89.4	17.6	19.7	50	89.4	8.6	9.6	-21.1
243	Pyraflufen-ethyl	0.9962	0.1-50	10	91.6	0.6	0.7	50	97.9	9.0	9.2	-17.9

244	Pyrazolate	0.9963	0.1-50	10	90.2	16.2	17.9	50	90.1	11.9	13.3	-3.4
245	Pyrazophos	0.9963	0.1-50	5	80.6	3.3	4.1	50	97.0	7.4	7.7	-7.8
246	Pyrazoxyfen	0.9959	0.1-50	5	88.8	5.0	5.6	50	93.8	4.3	4.6	-10.9
247	Pyribencarb	0.9974	0.1-50	5	87.6	8.1	9.3	50	87.2	7.2	8.3	14.3
248	Pyributicarb	0.9962	0.1-50	2.5	83.0	2.3	2.8	50	85.0	7.9	9.3	-23.5
249	Pyridaben	0.9975	0.1-50	2.5	100.8	2.1	2.0	50	78.1	5.5	7.1	-32.9
250	Pyriftalid	0.9981	0.1-50	5	85.7	6.5	7.6	50	97.5	7.3	7.5	-23.3
251	Pyrimethanil	0.9981	0.1-50	5	97.2	11.4	11.7	50	89.2	5.9	6.6	-26.4
252	Pyrimidifen	0.9973	0.1-50	2.5	115.6	3.3	2.8	50	85.1	5.5	6.5	-25.4
253	Pyriofenone	0.9977	0.1-50	2.5	107.0	17.4	16.2	50	94.7	8.9	9.4	-22.1
254	Pyriproxyfen	0.9981	0.1-50	2.5	91.1	2.3	2.5	50	86.8	4.7	5.4	-22.3
255	Pyroquilon	0.9968	0.1-50	2.5	111.9	3.4	3.1	50	86.3	6.0	6.9	-23.8
256	Quizalofop-ethyl	0.9978	0.1-50	2.5	82.6	5.5	6.7	50	80.0	8.6	10.7	-21.3
257	Sedaxane_cis	0.9907	0.1-50	2.5	91.7	11.1	12.1	50	91.8	10.9	11.8	-10.3
258	Sedaxane_trans	0.9912	0.1-25	1	86.6	6.4	7.4	50	94.3	3.8	4.0	5.5
259	Sethoxydim	0.9964	0.5-50	2.5	107.4	14.8	13.8	50	81.5	14.2	17.4	-17.3
260	Simetryn	0.9973	0.1-50	2.5	116.5	0.7	0.6	50	94.1	13.1	13.9	-15.9
261	Spinetoram (J)	0.9977	0.1-50	10	81.4	10.3	12.6	50	84.5	6.8	8.1	-30.4
262	Spinosyn A	0.9977	0.1-50	2.5	107.3	10.9	10.2	50	81.6	8.1	9.9	-28.5

263	Spinosyn D	0.9921	0.1-50	5	91.1	10.1	11.0	50	83.7	13.7	16.4	-24.6
264	Spirodiclofen	0.9977	0.1-50	2.5	94.6	5.7	6.0	50	74.0	2.7	3.6	-22.8
265	Sulfotep	0.9970	0.1-50	5	77.6	6.3	8.2	50	102.8	9.7	9.5	-26.7
266	Sulprofos	0.9976	0.1-50	10	85.0	6.7	7.8	50	75.2	3.5	4.6	-24.0
267	Tau-fluvalinate	0.9901	0.25-50	5	115.3	9.7	8.4	50	73.7	8.2	11.1	-25.0
268	Tebufenpyrad	0.9946	0.5-50	10	76.4	13.3	17.4	50	80.0	8.4	10.6	-21.4
269	Tebufloquin	0.9915	0.1-50	10	71.5	3.4	4.8	50	93.7	9.3	10.0	-21.6
270	Tebuthiuron	0.9956	0.1-50	10	79.1	3.6	4.6	50	77.9	7.9	10.1	-12.0
271	Tepraloxydim	0.9958	0.5-50	10	119.2	20.4	17.2	50	97.9	8.0	8.2	-22.8
272	Terbufos-oxon-sulfone	0.9975	0.1-50	1	112.4	15.4	13.7	50	76.7	4.2	5.5	-26.1
273	Terbufos-oxon-sulfoxide	0.9965	0.1-50	2.5	111.5	6.3	5.6	50	84.9	7.4	8.7	-18.3
274	Terbufos-sulfone	0.9960	0.5-50	5	102.9	7.2	7.0	50	89.4	6.0	6.7	-13.2
275	Terbufos-sulfone	0.9912	1-50	10	83.0	9.6	11.5	50	103.9	9.6	9.2	-28.3
276	Terbufos-sulfoxide	0.9965	0.1-50	10	104.2	2.7	2.6	50	97.8	7.8	7.9	-21.8
277	Terbuthylazine	0.9957	0.1-50	5	78.6	7.0	8.9	50	104.2	12.9	12.4	-26.5
278	Terbutryn	0.9971	0.1-50	2.5	80.6	13.8	17.2	50	92.0	6.6	7.2	-17.8
279	Thenylchlor	0.9960	0.1-50	5	83.6	13.2	15.8	50	96.0	6.2	6.5	-17.8
280	Thiabendazole	0.9965	0.1-50	5	101.2	0.8	0.8	50	80.4	3.2	4.0	-27.1
281	Thiacloprid	0.9968	0.1-50	2.5	104.6	3.6	3.4	50	88.0	6.3	7.2	-24.7

282	Thiamethoxam	0.9959	0.1-50	5	101.8	4.3	4.2	50	81.7	9.2	11.2	-16.3
283	Thiazopyr	0.9910	0.1-50	5	78.9	11.1	14.1	50	108.9	16.1	14.8	-15.6
284	Thidiazuron	0.9967	0.1-50	2.5	103.4	10.9	10.5	50	77.1	3.9	5.1	-4.5
285	Thidiazuron	0.9977	0.1-50	2.5	75.4	10.6	14.0	50	84.2	7.9	9.4	-9.2
286	Thiobencarb	0.9980	0.1-50	2.5	89.7	7.2	8.0	50	90.4	5.7	6.3	-18.8
287	Thionazin	0.9976	0.1-50	10	112.8	14.9	13.2	50	94.8	10.0	10.6	-24.9
288	Triafamone	0.9938	0.1-50	10	95.1	4.9	5.2	50	100.4	13.3	13.2	-31.2
289	Triazophos	0.9964	0.1-50	5	88.1	0.9	1.0	50	97.6	9.1	9.3	-14.5
290	Tribufos	0.9977	0.1-50	2.5	78.7	2.6	3.3	50	76.1	4.2	5.6	-35.9
291	Trifloxystrobin	0.9979	0.1-50	2.5	89.5	7.2	8.1	50	91.9	8.0	8.7	-15.8
292	Triflumizole	0.9976	0.1-50	5	78.5	6.7	8.6	50	92.0	0.8	0.8	-21.8
293	Triflumuron	0.9916	0.1-25	5	94.9	18.3	19.3	50	88.3	17.5	19.9	-7.5
294	Valifenalate	0.9987	0.1-50	2.5	117.2	13.3	11.4	50	95.8	9.7	10.1	-13.0
295	Vamidothion	0.9977	0.1-50	2.5	101.6	5.3	5.3	50	85.2	9.9	11.6	-20.7
296	Vernolate	0.9972	0.5-50	10	94.5	18.6	19.7	50	83.0	0.9	1.1	-6.2
297	XMC	0.9973	0.1-50	2.5	98.5	11.6	11.8	50	94.6	5.7	6.1	-31.6
298	Zoxamide	0.9974	0.1-50	2.5	88.8	16.0	18.0	50	94.9	8.1	8.5	-16.7

SD: Standard Deviation

RSD: Relative Standard Deviation
